# Metabolic profiling of *Vitex Pubescens* Vahl bark *via* UPLC-ESI-QTOF/MS/MS analysis and evaluation of its antioxidant and acetylcholinesterase inhibitory activities

**DOI:** 10.1186/s12906-024-04520-3

**Published:** 2024-06-14

**Authors:** Safa Abdelbaset, Iriny M. Ayoub, Osama G. Mohamed, Ashootosh Tripathi, Omayma A. Eldahshan, Dina M. El-Kersh

**Affiliations:** 1 Pharmacognosy Department, Faculty of Pharmacy, The British University in Egypt, Cairo, 11837 Egypt; 2 Pharmacognosy Department, Faculty of Pharmacy, Ain Shams University, Cairo, 11566 Egypt; 3 Pharmacognosy Department, Faculty of Pharmacy, Cairo University, Kasr el Aini St., Cairo, 11562 Egypt; 4 Natural Products Discovery Core, Life Sciences Institute, University of Michigan, Ann Arbor, MI 48109 USA; 5 Department of Medicinal Chemistry, College of Pharmacy, University of Michigan, Ann Arbor, MI 48109 USA; 6 Center for Drug Discovery Research and Development, Ain Shams University, Cairo, Egypt; 7 Drug Research and Development Group (DRD-G), The British University in Egypt (BUE), Cairo, 11837 Egypt

**Keywords:** *Vitex pubescens* bark, Alzheimer’s, Acetylcholinesterase, UPLC-ESI-QTOF/MS/MS, Polar fractions, Antioxidant, Ellman’s assay, AchE inhibitory activity

## Abstract

**Background:**

Alzheimer’s disease is a neurodegenerative age-related disease that primarily affects the elderly population leading to progressive memory impairments and neural deficits. It is counted as a major cause of geriatric dependency and disability. The pathogenesis of Alzheimer’s disease incidence is complex and involves various hypotheses, including the cholinergic hypothesis, deposition of *β*-amyloid plaques, neuroinflammation, oxidative stress, and apoptosis. Conventional treatments such as donepezil aim to delay the symptoms but do not affect the progression of the disease and may cause serious side effects like hepatoxicity. The use of natural candidates for Alzheimer’s disease treatment has drawn the attention of many researchers as it offers a multitargeted approach.

**Methods:**

This current study investigates the metabolic profiles of total defatted methanolic extract of *Vitex pubescens* bark and its polar fractions, viz. ethyl acetate and *n*-butanol, using ultra-performance liquid chromatography-electrospray ionization-quadrupole time-of-flight tandem mass spectrometry(UPLC-ESI-QTOF/MS/MS) technique as well as evaluate the antioxidant using free radical scavenging assays, viz. DPPH and ABTS assays and in-vitro acetylcholinesterase inhibitory activities using Ellman’s microplate assay.

**Results:**

Metabolic profiling revealed a total of 71, 43, and 55 metabolites tentatively identified in the defatted methanolic extract, ethyl acetate, and *n*-butanol fractions, respectively. Phenolic acids were the most abundant class, viz. benzoic acids, and acyl quinic acid derivatives followed by flavonoids exemplified mainly by luteolin-*C*-glycosides and apigenin-*C*-glycosides. Quantification of the total phenolic and flavonoid contents in the total defatted methanolic extract confirmed its enrichment with phenolics and flavonoids equivalent to 138.61 ± 9.39 µg gallic acid/mg extract and 119.63 ± 4.62 µg rutin/mg extract, respectively. Moreover, the total defatted methanolic extract exhibited promising antioxidant activity confirmed through DPPH and ABTS assays with a 50% inhibitory concentration (IC_50_) value equivalent to 52.79 ± 2.16 µg/mL and 10.02 ± µg/mL, respectively. The inhibitory activity of acetylcholine esterase (AchE) was assessed using in-vitro Ellman’s colorimetric assay, the total defatted methanolic extract, ethyl acetate, and *n*-butanol fractions exhibited IC_50_ values of 52.9, 15.1 and 108.8 µg/mL that they proved the significant inhibition of AchE activity.

**Conclusion:**

The results obtained herein unraveled the potential use of the total methanolic extract of *Vitex pubescens* bark and its polar fractions as natural candidates for controlling Alzheimer’s disease progression.

**Supplementary Information:**

The online version contains supplementary material available at 10.1186/s12906-024-04520-3.

## Introduction

Alzheimer’s disease (AD) is an irreversible neurodegenerative disorder marked by gradual and progressive memory loss, compromised cognitive and neuronal dysfunction, with subsequent deteriorated behavior-related skills [1]. It mainly affects the elderly population over 65 years [2]. The World Health Organization [3] recorded Alzheimer’s disease as the seventh leading cause of mortality worldwide. It is estimated that more than 55 million people are currently diagnosed with AD, and the prevalence is speculated to surge to 78 million by 2030 [3]. AD is regarded as one of the major contributors to geriatric dependency, which globally increases healthcare’s economic burden [4]. The pathophysiology of AD is sophisticated with multifactorial hypotheses encompassing the cholinergic hypothesis and deposition of extracellular amyloid *β* (A*β*) plaques alongside other causative factors such as oxidative stress, neuroinflammation, and apoptosis [2, 5]. Firstly, the cholinergic hypothesis is explained by the progressive decline in the acetylcholine (Ach) neurotransmitter which is responsible for neuronal activity, plasticity, and network connectivity. This major concern in acetylcholine level occurs due to the degeneration of cholinergic neurons, appearance of cholinergic lesions, or overactivity of acetylcholinesterase enzyme (AchE) that subsequently leads to the depletion of the level of Ach [6, 7]. Secondly, the deposition of A*β* plaques is one of the hallmarks of Alzheimer’s disease pathogenesis that causes brain structure abnormalities [2, 8]. The main triggering factor of neurodegenerative diseases is increasing oxidative stress [9]. The brain has a plentiful amount of polyunsaturated fatty acids besides the presence of aerobic media, transition metals, and the reduction in antioxidant enzymatic activity that leads to an imbalance in the redox system, accumulation of oxygen reactive species (ROS), increased lipid peroxidation and DNA oxidation and eventually oxidative damage [1, 10]. Many predisposing factors as the accumulation of ROS and nitrogen-reactive species (NRS) impair the activity of mitochondria and activate the apoptotic mediators that contribute to the progressive degeneration of neurons [1]. Conventional therapies improve the symptoms but have not shown any effect on delaying the disease progression [2]. The current conventional therapeutic class commonly prescribed is acetylcholinesterase inhibitors as donepezil, galantamine, and rivastigmine which depend on restoring Ach level by inhibiting the AchE degradable enzyme [5]. Unfortunately, detrimental adverse effects such as gastrointestinal illnesses and hepatotoxicity are considered a driving cause for discovering new entities for the prevention or treatment of AD [11]. Owing to the complexity of AD, some modern therapeutic strategies offer a multitargeted approach for treating neurodegenerative disorders that could be achieved through natural herbal products that act on various mechanisms of pathogenesis [5, 12–14]. The potent antioxidant activity of some phytochemical classes, viz. polyphenolics and the anti-inflammatory properties of natural products serve in the prevention of neuron inflammation, formation and aggregation of A*β* plaques [5, 15, 16]. Moreover, some natural products also act on the Ach hypothesis by exhibiting inhibitory activity of the acetylcholinesterase enzyme that help ameliorate the cognitive dysfunction induced by AD [6].

*Vitex pubescens* Vahl (syn. *Vitex pinnata* Linn), a member of family Lamiaceae [17], is a medium-sized tree with a height ranging from 25 to 30 m [18]. It is widely distributed in tropical regions of Asia including Malaysia, Indonesia, the Philippines and Pakistan [19]. *Vitex pubescens* (*V. pubescens*) is commonly known in Malaysia as “Halban” with ethnopharmacological uses as anti-pyretic, anti-hypertensive, analgesic, wound healing, and for the treatment of gastrointestinal ailments [20, 21]. Many phytochemical studies were undergone to investigate the phytochemical profile as well as evaluate the biological activities of *V. pubescens* leaf extracts [20, 22–24]. Prior studies investigating *V. pubescens* leaves reported that the abundance of phytochemical classes namely, ecdysteroids, triterpenoids, iridoid glycosides and flavonoid compounds [22, 23]. Unfortunately, chemical profiling to explore the bioactive metabolites of *V. pubescens* bark remains insufficient and has not been fully identified despite the long history of its traditional uses. Therefore, this study aimed to investigate the metabolic profile of the total defatted methanol extract of *V. pubescens* Vahl bark as well as its polar fractions, viz. ethyl acetate and *n*-butanol fractions for the first time using Ultra-performance liquid chromatography-electrospray ionization-quadrupole time-of-flight-tendem mass spectrometry (UPLC-ESI-QTOF-MS/MS). Moreover, the anti-Alzheimer’s disease potential of the total defatted methanolic extract was evaluated in-vitro using Ellman’s microplate assay.

## Materials and methods

### General chemical and solvents

*n*-hexane, methanol, dichloromethane (DCM), ethyl acetate, *n*-butanol, formic acid, acetonitrileand ethanol were provided by Fisher Scientific., Loughborough, United Kingdom. DPPH (2,2-diphenyl-1-picryl-hydrazyl-hydrate), DTNB Ellman’s reagent, acetylthiocholine iodide substrate and ABTS reagent were purchased from Sigma-Aldrich, St. Louis, United States. Anticholinesterase enzyme was obtained from *Electrophorus electricus*, purchased from Sigma Aldrich, St. Louis, United States, Cat No. 3389.

### Plant materials

*V. pubescens* Vahl bark was purchased and authenticated from the herbal company, ETHNO Resources Sdn. Bhd., Malaysia. A voucher specimen (No. PHG-P-VP-302) was deposited in the herbarium of the Pharmacognosy Department, Faculty of Pharmacy, Ain Shams University, Cairo, Egypt.

### Extraction and fractionation procedures

Four kilograms of *V. pubescens* Vahl bark powder were firstly defatted with *n*-hexane (3 × 10 L) using cold maceration method for seven days till exhausting, filtereted and concentrated *in-vacuo* using a rotatory evaporator (Büchi Labortechnik GmbH, Essen, Germany). The defatted powder was then exhaustively extracted with absolute methanol (7 × 15 L) by cold maceration at ambient temperature for three days, concentrated *in-vacuo* at 40 °C to yield 185 g of total defatted methanol extract (VT). The VT (180 g) was subjected to successive liquid-liquid fractionation using solvents of increasing polarity, viz. *n*-hexane (4 × 1 L) followed by DCM (6 × 1 L), ethyl acetate (3 × 1 L) and *n*-butanol saturated with water (10 × 1 L), concentrated *in-vacuo* at a temperature below 55 °C till dryness to obtain four different fractions, viz. *n*-hexane, DCM, ethyl acetate and *n*-butanol fractions.

### Characterization of phytochemicals using UPLC–ESI-QTOF-MS/MS analysis

Metabolites characterization was performed using high-resolution Agilent LC-MS system consisting of the Agilent 1290 Infinity II Ultra Performance Liquid Chromatography (UPLC) coupled with Agilent 6545 Electrospray Ionization-quadrupole time-of-flight MS/MS (ESI-QTOF-MS/MS) using both negative and positive ionization modes. For chromatographic analysis, Kinetex phenyl-hexyl column (1.7 μm, 2.1 × 50 mm) was used. ESI parameters were settled as follows: source voltage at 3.5 kV, capillary temperature at 320 ºC and a sheath gas flow rate of 11 L/ min. Aliquots (1µL) ofthe defatted total methanol extract (VT), ethyl acetate (VE) and *n*-butanol (VB) fractions were prepared individually as (1 mg/mL MeOH) and each of them was injected on the selected column eluted with a flow rate of 0.4 mL/min. Firstly, the elution was isocratic for one minute with 100% of solvent A (100% H_2_O + 0.1% formic acid) followed by a linear gradient elution for 6 min till 100% solvent B (95% acetonitrile + 5% H_2_O + 0.1% formic acid). The full scan of ions detection was set as following: an isolation width 1.3 ~ *m/z*, an intensity above 1000 counts at 6 scans/s with 9 selected precursors per cycle and ramped collision energy (5 × *m/z/*100 + 10 eV) was used. The internal lock masses used for positive mode were purine [(M + H)^+^ at *m/z* 121.050873, C_5_H_4_N_4_] and 1 H,1 H,3 H-tetrafluoropropoxy phosphazene [(M + H]^+^) at *m/z* 922.009798, C_18_H_18_F_24_N_3_O_6_P_3_] whereas, the internal lock masses used for negative mode were trifluoroacetic acid (TFA) [(M-H)^−^ at *m/z* 112.985587, C_2_HF_3_O_2_] and 1 H,1 H,3 H-tetrafluoropropoxy phosphazene [(M + TFA-H)^−^ at *m/z* 1033.988109, C_18_H_18_F_24_N_3_O_6_P_3_]. Data acquisition was performed using Agilent Mass Hunter workstation software v B.06.00.

### Total phenolic content determination (TPC)

The total phenolic content for VT was determined spectrophotometrically using the Folin-Ciocalteu method as described by Attard [25, 26]. The absorbance of the blue complex color was measured at λ_max_ 630 nm spectrophotometrically using a microplate reader FluoStar Omega. The samples were prepared in triplicate, and the mean absorbance value was calculated. The results were represented as mean ± SD. The results were expressed in terms of gallic acid equivalent [27] per milligram of extract (µg GAE/mg extract). The same procedure was repeated for the standard, gallic acid and the calibration curve was constructed as the concentration of 25–100 µg/mL. The mean absorbance of the gallic acid was calculated according to the following equation:

$$\mathrm{Absorbance}=0.0027\;\mathrm{gallic}\;\mathrm{acid}\;\mathrm{concentration}-0.0421$$

$$\mathrm R^2=0.9994$$

### Total flavonoids content determination (TFC)

The total flavonoid content was measured and quantified for VT using aluminum chloride assay which depends on spectrophotometric analysis [28, 29]. The reaction mixture was prepared and incubated at room temperature for 5 min, after which the yellow color formed was measured at λ_max_ = 420 nm. The same procedure was repeated for the standard, rutin, and the calibration curve was constructed at the concentration range of (7.5–1000 µg/mL). The samples were prepared in triplicate, and the mean absorbance value was calculated and represented as mean ± SD. The results were expressed in terms of rutin equivalent (RE) per milligram of extract (µg RE/mg extract). The mean absorbance of rutin is calculated according to the following equation:

$$\mathrm{Absorbance}=0.0019\;\mathrm{rutin}\;\mathrm{concentration}-0.0127$$

$$\mathrm R^2=0.9985$$

### Evaluation of antioxidant activity

#### DPPH free radical scavenging assay

The DPPH (2,2-diphenyl-1-picryl-hydrazyl-hydrate) assay was employed to assess the antioxidant properties of the plant extract. In a 96-well microplate, 100 µL of a freshly prepared 0.1% DPPH solution in methanol was added and mixed with 100 µL of VT in ethanol at different concentrations (15.625, 31.25, 62.5, 125 and 250 µg/mL). The reaction mixture was incubated at room temperature for 30 min then the absorbance of the yellow color intensity was measured at λ_max_ 540 nm spectrophotometrically using a microplate reader FluoStar Omega. Trolox was used as a standard solution prepared with five concentrations ranging from (1.25–12.5 µg/mL). The percentage of DPPH activity inhibition was calculated according to the following equation.

$$\mathrm{Percentage}\;\mathrm{inhibiton}=\frac{\mathrm{Average}\;\mathrm{absorbance}\;\mathrm{of}\;\mathrm{blank}-\mathrm{average}\;\mathrm{absorbance}\;\mathrm{of}\;\mathrm{the}\;\mathrm{test}}{\mathrm{Average}\;\mathrm{absorbance}\;\mathrm{of}\;\mathrm{blank}}\times100$$

The percent inhibition is plotted against the concentrations and 50% inhibition concentration (IC_50_) was calculated using Graph pad Prism 6.

### Free radical scavenging activity (ABTS) assay

ABTS (2,20 -azinobis (3-ethylbenzthiazoline-6-sulphonic acid)) assay was conducted according to the method of [30]. Firstly, the ABTS solution was prepared by dissolving ABTS (192 mg) in 50 mL distilled water,1 mL of solution was added to 17 µL of 140 mM potassium persulphate and incubated for 24 h. 1 mL of the reaction mixture was completed with 50 mL methanol to obtain the final ABTS working concentration. In 96 well plate, 190 µL of freshly prepared ABTS reagent was mixed with 10 µL of VT sample (prepared at a concentration of 14 mg/mL in 70% ethanol) or standard stock solution of Trolox (prepared at a concentration of 2 mg/mL). The mixture was then incubated in the dark for 30 min at room temperature. At the end of the incubation period, the reduction of ABTS color intensity was measured at 734 nm using a microplate reader FluoStar Omega. The data was represented as mean ± SD and the percentage inhibition of ABTS radical cation by the extract was calculated using the following equation:

$$\mathrm{Percantage}\;\mathrm{inhibition}=\frac{\mathrm{Average}\;\mathrm{absorbance}\;\mathrm{of}\;\mathrm{blank}-\mathrm{average}\;\mathrm{absorbance}\;\mathrm{of}\;\mathrm{the}\;\mathrm{test}}{\mathrm{Average}\;\mathrm{absorbance}\;\mathrm{of}\;\mathrm{blank}}\times100$$

### Acetylcholinesterase inhibitory activity using Ellman’s microplate assay

The in-vitro assessment of AchE inhibitory activity was evaluated according to Ellman’s microplate assay [31]. The samples of VT, VE and VB as well as the standard drug, donepezil were prepared in a set of 8 concentrations (500 –3.9 µg/mL). In a 96 microplate, an aliquot (10 µL) of DTNB (5,5´-Dithiobis [2-Nitrobenzoic Acid]) (0.4mM diluted in buffer:100 mM tris buffer, pH = 7.5) was added to mixture solutions of 20 µL acetylcholinesterase enzyme (0.02 U/mL: 50 Mm tris buffer in 1% bovine serum albumin, pH = 7.5) and 20 µL of different concentrations of sample or standard and 140 µL of 0.1 M sodium phosphate buffer. Consequently, 20 µL of acetylthiocholine iodide (ACTI) (0.4mM diluted in buffer) substrate was added. Each sample was conducted in triplicate. The absorbance was measured using microplate reader FluoStar Omega at 412 nm. The data was presented as mean ± SD and was analyzed using Microsoft Excel®. The IC_50_ value was calculated using GraphPad Prism software.

### Statistical analysis

Data were presented as mean ± SD of the performed triplet experiments. Results of AchE inhibitory activity was performed using dose response nonlinear regression test. The calculated IC_50_ (95% confidence interval) was also compared using one way ANOVA followed by Dunnett’s test with significance *P* value (< 0.05) All Data presentation was via GraphPad Prism Software (Inc. San Diego, CA, version 8.0).

## Results and discussion

### Extraction and fractionation procedures

The extraction procedure yielded 185 g (4.6% *w/w*) of total defatted methanol extract. The dried solvent-free fractions were weighed yielding, *n*-hexane fraction 10 g (5.4% *w/w*), dichloromethane fraction 21.1 g (11.7%), ethyl acetate fraction 5.5 g (2.97%), and *n*-butanol fraction 110 g (59.4%). Dried extract and fractions were kept at -20 °C for further analysis.

### Characterization of phytochemicals using UPLC-ESI-QTOF/MS/MS analysis

**Fig. 1:**
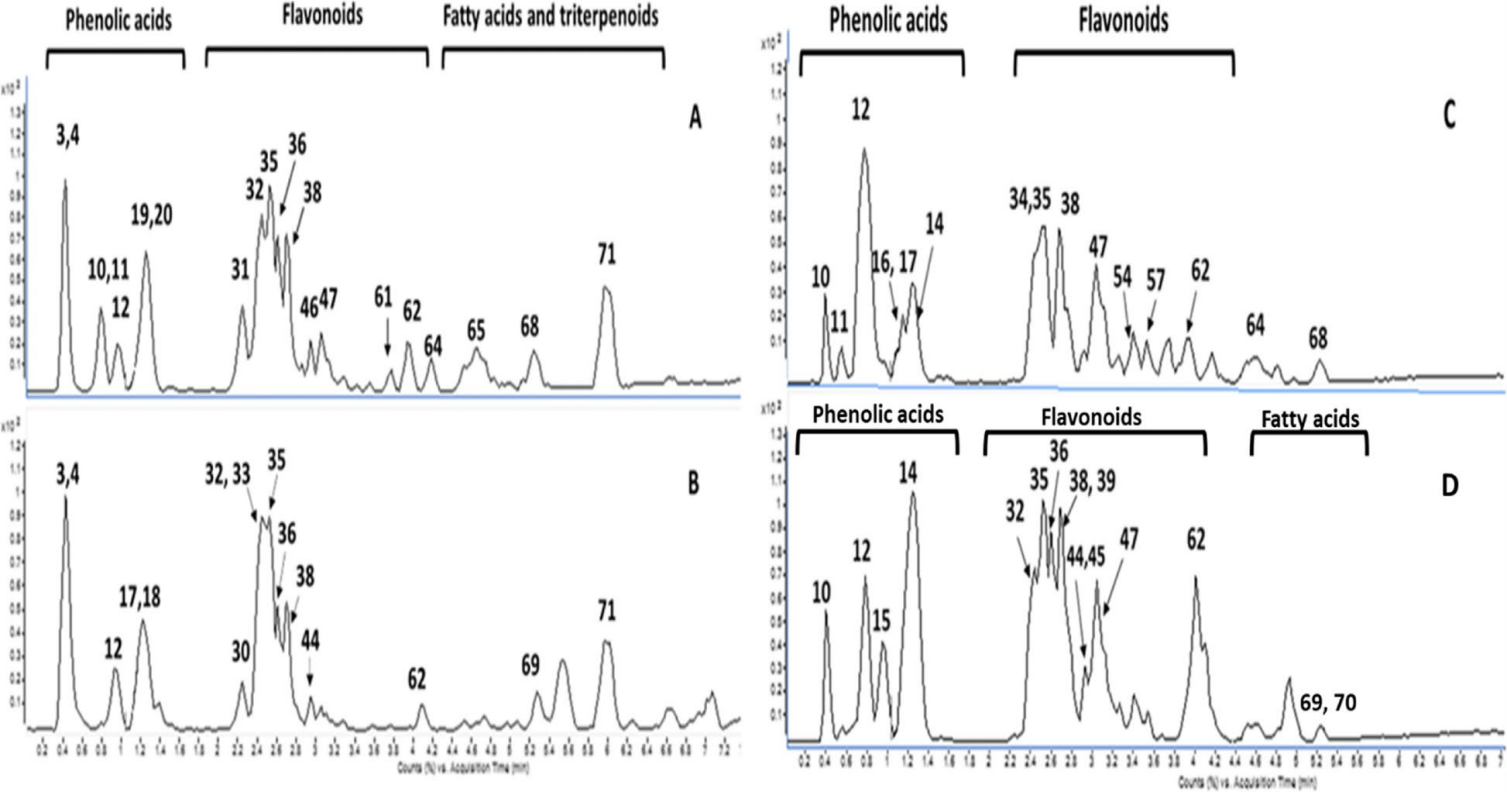
UPLC-ESI-QTOF/MS/MS chromatograms of *V. pubescens* methanol extract (**A**) total defatted methanol extract in the negative ion mode, **B** defatted methanol extract in the positive ion mode, **C** ethyl acetate fraction in the negative ion mode and **D** *n*-butanol fraction. The peaks are annotated as listed in Table 1

UPLC-ESI/MS/MS offered a robust and reproducible analytical technique that was employed to tentatively identify and characterize different bioactive secondary metabolites [32]. The identification of metabolites was based on comparing the deprotonated/protonated molecular ions and the fragmentation pattern, including the base peak and the major high intensity peaks with those reported in the literature. The UPLC-ESI-QTOF/MS/MS analysis of the defatted methanol extract of *V. pubescens* bark and its polar fractions, viz. ethyl acetate and *n*-butanol fractions revealed the presence of bioactive metabolites belonging to the various mermaid phytochemical classes that were exemplified in polyphenolics such as simple organic acids, phenolic acids, and flavonoids besides the presence of fatty acids and triterpenoids. The order of elution of various chromatographic peaks occurred with decreasing polarity starting with organic acids, and simple phenolics, followed by flavonoid glycosides then aglycones and finally fatty acids and triterpenoids as depicted in Fig. 1. Considering the identified phytochemical metabolites, phenolic acids and flavonoids ionized with higher sensitivity in the negative ion mode, whereas triterpenoids were ionized preferentially in the positive ion mode [33, 34].

**Fig. 2:**
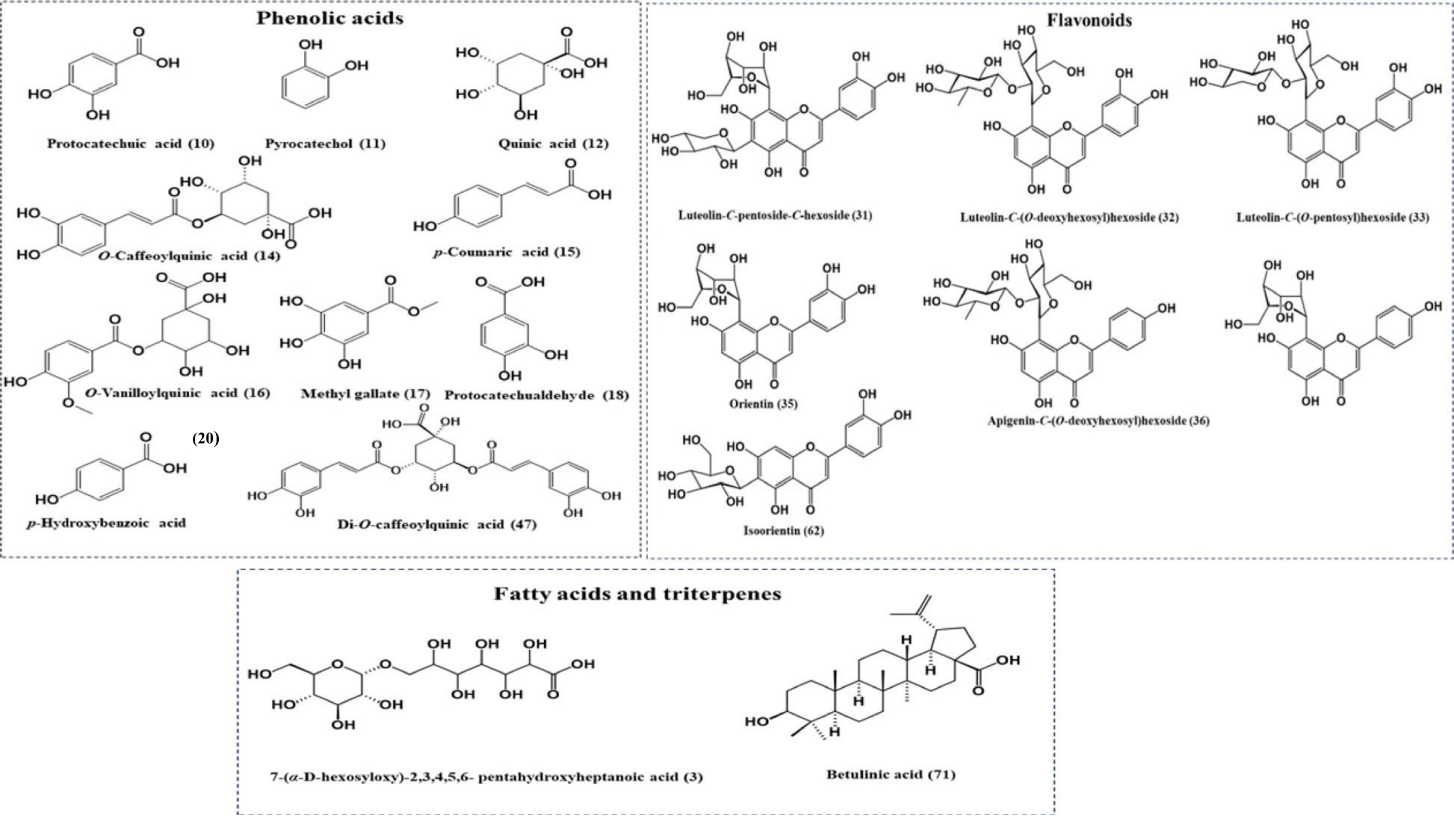
The structures of major identified peaks using UPLC-ESI-QTOF/MS/MS from the defatted methanol extract, ethyl acetate and *n*-butanol fractions of *V. pubescens *bark

The detailed identification of the total defatted methanol extract and its ethyl acetate and *n*-butanol fractions are listed in Table 1. Results revealed a total of 71 metabolites identified in the total defatted methanolic extract. Upon comparing the total defatted methanolic extract with its ethyl acetate and *n*-butanol fractions, a total of 43 and 55 metabolites have been identified, respectively. Regarding the phytochemical classes of the identified metabolites from total defatted methanol extract and its fractions, it would be conducted that the total defatted methanol extract was enriched with phenolic compounds, viz. organic, phenolic acids (39 metabolites) and flavonoids (21 metabolites) as well as triterpenes (6 metabolites) and fatty acid derivatives (5 metabolites). Whereas for polar fractions, the ethyl acetate fraction was enriched with phenolic compounds, viz. organic, phenolic acids (28 metabolites) and flavonoids (15 metabolites) and *n*-butanol fraction was enriched with phenolic compounds, viz. organic, phenolic acid (29 metabolites) and flavonoids (21 metabolites), also fatty acid derivatives (5 metabolites). The major identified metabolites were illustrated in Fig. 2. A detailed description of the MS/MS spectra of identified metabolities are displayed as Supplementary material in Fig. S1.

**Table 1:** Metabolites identified by UPLC-ESI-QTOF/MS/MS analysis of total defatted methanol extract, ethyl acetate, and *n*-butanol fractions of *V. pubescens* bark

Peak no.	tR(min)	[M-H]^−^	[M-H] ^+^	Formula	MS^*n*^ (m/z) negative mode	MS^*n*^ (m/z) positive mode	Metabolite	Class	Total defatted methanolic extract	Ethyl acetate fraction	*n*-Butanol Faction	Reference
1.	0.185	-	116.9753	C_4_H_4_O_4_	-	72.9381	Fumaric acid	Organic acid	√	-	-	[32]
2.	0.225	149.9068	-	C_4_H_6_O_6_	131	-	Tartaric acid	Organic acid	√	-	-	[35]
3.	0.412	387.1140	-	C_13_H_24_O_13_	341, 221, 179 161, 89	-	7-(*α*-D-hexosyloxy)-2,3,4,5,6- pentahydroxyheptanoic acid	Fatty acid	√	-	-	[36]
4.	0.470	533.1719	-		191, 173, 127, 85	-	Quinic acid derivative	Phenolic acid	√	√	√	[37]
5.	0.586	169.0133	-	C_7_H_6_O_5_	125	-	Gallic acid	Phenolic acid	√	√	√	[38]
6.	0.586	125.0235	-	C_6_ H_6_O_3_	97, 79	-	Pyrogallol	Polyphenol	√	√	√	[39]
7.	0.665	311.0767	-	C_14_ H_16_O_8_	191, 173, 93	-	*O*-*p*-Hydroxybenzoyl quinic acid	Phenolic acid	√	-	-	[40]
8.	0.679	299.0764	-	C_13_H_16_O_8_	137	-	*p*-Hydroxybenzoic acid-*O*- hexoside	Phenolic acid	√	√	√	[38]
9.	0.700	167.0341	169.0485	C_8_H_8_O_4_	152, 123, 108, 91	125, 93 65	Hydroxy-methoxy benzoic acid	Phenolic acid	√	√	√	[41]
10.	0.712	153.0185	-	C_7_H_6_O_4_	109	-	Protocatechuic acid	Phenolic acid	√	√	√	[38]
11.	0.739	109.0289	-	C_6_H_6_O_2_	108, 91	-	Pyrocatechol	Polyphenol	√	√	√	[32]
12.	0.769	191.0553	-	C_7_H_12_O_6_	173, 127, 93, 85	-	Quinic acid	Phenolic acid	√	√	√	[42]
13.	0.818	329.0875	-	C_14_H_18_O_9_	167, 152	-	Vanillic acid-*O*- hexoside	Phenolic acid	√	√	√	[38]
14.	0.831	353.0873	-	C_16_H_18_O_9_	191, 179, 135	-	*O*-Caffeoylquinic acid	Phenolic acid	√	√	√	[43]
15.	0.962	163.0404	-		93	-	*p*-Coumaric acid	Phenolic acid	√	-	√	[44]
16.	1.194	341.0881	-	C_15_H_17_O_9_	191, 173, 167	-	*O*-Vanilloylquinic acid	Phenolic acid	√	-	-	[45]
17.	1.197	183.0291	185.0798	C_8_H_8_O_5_	124	153, 139, 125, 110	Methyl gallate	Phenolic acid	√	√	√	[46]
18.	1.226	-	139.0383	C_7_H_6_O_3_	-	121, 111, 95	Protocatechualdehyde	Phenolic acid	√	√	√	[47]
19.	1.252	629.2085	-		137, 93	-	Hydroxybenzoic Acid derivative	Phenolic acid	√	√	√	[36, 37]
20.	1.297	137.0237	139.0382	C_7_H_6_O_3_	122, 93	95, 77	*p*-Hydroxybenzoic acid	Phenolic acid	√	√	√	[38]
21.	1.384	-	163.0383	C_9_H_7_O_3_	-	135, 117, 107, 89	Umbelliferone	Coumarins	√	-	-	[48]
22.	1.390	353.0873	-	C_16_H_18_O_9_	191	-	*O*-Caffeoylquinic acid	Phenolic acid	√	√	√	[43]
23.	1.532	152.0109	-	C_7_H_6_O_4_	108	-	Methyl hydroxybenzoic acid	Phenolic acid	√	√	√	[36, 37]
24.	1.535	167.0341	-	C_8_H_8_O_4_	152, 123, 108 91	-	Vanillic acid	Phenolic acid	√	√	√	[38]
25.	1.649	609.1455	-	C_27_H_29_ O_16_	519, 489, 429 399, 369	-	Luteolin-di-*C*-hexoside	Flavones	√	-	√	[49]
26.	1.784	197.0441	-	C_9_H_10_O_5_	167, 153, 123	-	Syringic acid	Phenolic acid	√	√	√	[38]
27.	2.081	593.1510	-	C_27_H_29_ O_15_	503, 473, 383 353	505, 355, 349 377, 325	Apigenin-di-*C*-hexoside	Flavones	√	-	√	[38]
28.	2.150	755.2029	-	C_33_H_39_O_20_	593, 473, 429, 357, 327, 309	-	Luteolin-*C*-hexoside-*O*-hexoside-*O*-deoxyhexoside	Flavones	√	√	√	[50]
29.	2.206	563.1742	-	C_26_H_27_O_14_	473, 443, 383 353	-	Apigenin-*C*-hexoside-*C*-pentoside	Flavones	√	√	√	[38]
30.	2.208	609.1455	611.1612	C_27_H_30_O_16_	489, 357, 339 327, 309	491, 359, 329 311, 299	Luteolin-*C*-hexoside-*O*-hexoside	Flavones	√	-	√	[51]
31.	2.257	579.1353	-	C_26_H_27_O_15_	519, 489, 459 429, 399, 369	-	Luteolin-*C*-pentoside-*C*-hexoside	Flavones	√	-	√	[49]
32.	2.353	593.1510	595.1645	C_27_H_30_O_15_	357, 327, 309	499, 413, 329 299	Luteolin-*C*-(*O*-deoxyhexosyl) hexoside	Flavones	√	√	√	[38, 49]
33.	2.480	579.1353	581.1504	C_26_H_27_O_15_	459, 429 357, 327, 309	353, 329, 299	Luteolin-*C*-(*O*-pentosyl) hexoside	Flavones	√	√	√	[49]
34.	2.503	895.1937	-	C_42_H_39_O_22_	447, 357, 327 285	-	Luteolin-*C*-hexoside dimer	Flavones	√	√	√	[37]
35.	2.650	447.0936	449.1076	C_21_H_20_O_11_	357, 339, 327 311, 297, 285	377, 329, 299	Luteolin-*C*-hexoside (Luteolin-8-*C*-glucoside, orientin)	Flavones	√	√	√	[37]
36.	2.685	577.1564	579.1702	C_27_H_30_O_14_	413, 311, 293	433, 379, 337 313, 295	Apigenin-*C-*(*O*-deoxyhexosyl)hexoside	Flavones	√	√	√	[49]
37.	2.745	607.1669	-	C_28_H_32_O_15_	487, 443, 353 341, 323	-	Chrysoeriol-*C-*(*O*-deoxyhexosyl)hexoside	Flavones	√	-	√	[49]
38.	2.750	431.0983	433.1124	C_21_H_20_O_10_	341, 311, 283 269	397, 343, 313 283	Apigenin-*C*-hexoside (Apigenin-8-*C*-glucoside, vitexin)	Flavones	√	√	√	[37]
39.	2.782	729.1676	731.1800	C_34_ H_33_ O_18_	609, 561, 429 357, 339, 327 309	353, 329, 299	Vanilloyl-*C-(O*-pentosyl) hexosyl-luteolin	Flavones	√	√	√	[49]
40.	2.799	461.1086	-	C_22_H_22_O_11_	371, 341, 326 313, 298	-	Methoxy luteolin-*C*-hexoside	Flavones	√	√	√	[52]
41.	2.844	593.1510	595.1645	C_27_H_30_O_11_	285	343.0798 313.0687 287.0537	Luteolin-*O*-(*O*-deoxyhexosyl) hexoside	Flavones	√	-	√	[42]
42.	2.880	187.0971	-	C_9_H_16_O_4_	125	-	Azelaic acid	Organic acid derivatives	√	-	-	[53]
43.	2.934	533.1293	-		353, 335, 197 191 179, 173, 161, 135	-	*O*-Caffeoyl-*O*-syringoylquinic acid	Phenolic acid	√	√	-	[45]
44.	2.948	743.1830	745.1987	C_35_ H_35_ O_18_	623, 593, 575 443, 371, 353 341, 323	-	Vanilloyl-*C*-(*O*-pentosyl) hexosyl- chrysoeriol	Flavones	√	√	√	[38]
45.	2.987	713.1729	-	C_34_ H_33_ O_17_	563, 413, 341 323, 311, 293	-	Vanilloyl-*C*-(*O*-pentosyl) hexosyl-apigenin	Flavones	√	√	√	[54]
46.	3.041	503.1194	-	C_24_H_23_O_12_	341, 191, 179	-	*O*-Caffeoyl-*O*-vanilloylquinic acid	Phenolic acid	√	-	√	[45]
47.	3.055	515.1194	-	C_25_H_24_O_12_	191, 179, 173 135	-	Di-*O*-caffeoylquinic acid	Phenolic acid	√	√	√	[42]
48.	3.081	179.0344	181.0121	C_9_H_8_O_4_	135	153, 137, 107 97, 79	Caffeic acid	Phenolic acid	√	√	√	[38]
49.	3.165	173.0449	-	C_7_H_10_O_5_	173, 111, 93	-	Shikimic acid	Phenolic acid	√	√	-	[38]
50.	3.192	473.1085	-	C_20_H_26_O_13_	335, 311, 173 137	-	*O*-*p*-Hydroxybenzoyl-*O*-caffeoylquinic acid	Phenolic acid	√	-	√	[55]
51.	3.221	515.1194	-	C_25_H_24_O_12_	353, 191, 179, 173, 135	-	Di-*O*-caffeoylquinic acid isomer	Phenolic acid	√	√	√	[41]
52.	3.295	431.0983	433.1124	C_21_H_20_O_10_	341, 311, 283	397, 343,313 283	Apigenin-*C*-hexoside (Apigenin-6-*C*-glucoside, isoovitexin)	Flavones	√	√	√	[37]
53.	3.329	499.1247	-	C_22_H_28_O_13_	191, 179, 173 163	-	*O*-*p*‐Coumaroyl‐*O*‐caffeoylquinic acid	Phenolic acid	√	√	√	[56]
54.	3.449	529.1353	-	C_26_H_26_O_12_	367, 179, 161, 135	-	Methyl dicaffeoyl quinate	Phenolic acid	√	√	√	[41]
55.	3.497	497.1093	499.1228	C_25_H_22_O_11_	335, 179, 161 135	163	Dicaffeoylshikimic acid	Phenolic acid	√	√	√	[56]
56.	3.509	501.3216	-	C_30_H_46_O_6_	483, 457, 439 379	-	Pomaceic acid	Triterpene	√	-	-	[55]
57.	3.572	285.0397	287.0542	C_15_H_10_O_6_	151, 133	153, 135	Luteolin	Flavones	√	√	√	[57]
58.	3.623	327.2167	-	C_18_H_32_O_5_	229, 211, 171	-	9,12,13-Trihydroxyoctadeca-10(*E)*,15(*Z*)- dienoic acid	Hydroxy fatty acids	√	-	√	[41]
59.	3.633	707.1985	-	C_32_H_36_O_18_	353, 191, 179 173, 93	-	*O*-Caffeoylquinic acid dimer	Phenolic acid	√	-	√	[37]
60.	3.644	543.1507	-	C_27_H_28_O_12_	381, 179, 161, 135	-	Ethyl-di caffeoyl quinate	Phenolics	√	√	√	[41]
61.	3.811	329.2326	-	C_18_H_34_O_5_	229, 211, 171	-	9,12,13-Trihydroxyoctadec-10-enoic acid	Hydroxy fatty acids	√	-	√	[41]
62.	3.916	447.0930	449.1076	C_21_H_20_O_11_	357, 339, 327 311, 297, 285	377 329 299	Luteolin-*C*-hexoside (luteolin-6-*C*-glucoside, isoorientin).	Flavones	√	√	√	[37]
63.	3.963	-	473.3244	C_30_H_48_O_4_	-	455, 413, 409	Maslinic acid	Triterpenes	√	-	-	[58]
64.	4.460	487.3424	-	C_30_H_48_O_5_	469, 443, 407	-	Euscaphic acid	Triterpenes	√	-	-	[59]
65.	4.654	313.2376	-	C_17_H_14_O_6_	313, 295, 201 183	-	Dihydroxy-octadecenoic acid I	Hydroxy fatty acids	√	-	√	[60]
66.	4.923	-	457.3295	C_30_H_48_O_3_	-	411, 393	Ursolic acid	Triterpene	√	-	-	[58]
67.	5.049	116.9278	-	C_4_H_6_O_4_	99	-	Succinic acid	Phenolic acid	√	√	-	[61]
68.	5.104	471.3473	-	C_30_H_48_O_4_	453, 423, 407	-	Pomolic acid	Triterpene	√	-	-	[55]
69.	5.182	295.2268	297.3470	C18H32O3	277, 171	-	Hydroxy linoleic acid	Hydroxy fatty acids	√	-	√	[62]
70.	5.412	293.2116	-	C_18_H_30_O_3_	293, 275, 195 96	-	Hydroxy linolenic acid	Hydroxy fatty acids	√	-	√	[61]
71.	5.990	-	457.3301	C_30_H_48_O_3_	-	439, 411, 189, 175	Betulinic acid	Triterpene	√	-	-	[63]

(√) present and (-) absent

### Identification of organic acids

A total of six organic acids (peaks 1, 2,, 42, 49 and 67) were tentatively identified as fumaric acid, tartaric acid,, azelaic acid, shikimic acid and succinic acid. The identification relies on the fragmentation pattern characterized by decarboxylation (-44 Da) and/or dehydration (-18 Da) of the molecular ion peak [64]. Peak (1) [tR0.185 min, (M + H)^+^ at *m/z* 116.9753 (C_4_H_5_O_4_)^+^] displayed a base peak fragment ion at *m/z* 72.9381 [M + H-COO]^+^ which represents decarboxylation (-44 Da) of the molecular ion. Thus, peak (1) was assigned as fumaric acid [32]. Peak (2) [tR 0.225 min, (M-H)^−^ at *m/z* 149.9068, (C_4_H_5_O_6_)^−^] displayed a base peak fragment ion at *m/z* 131.8971 [M-H-H_2_O]^−^ which represents the loss of a water molecule (-18 Da) from the molecular ion peak. Thus, peak (2) was assigned as tartaric acid [35]. Peak (42) [tR 2.880 min, (M-H)^−^ at *m/z* 187.0971, (C_9_H_15_O_4_)^−^] displayed a characteristic base peak ion at *m/z* 125.0967 [M-H-COO-H_2_O]^−^. Thus, peak (42) was assigned as azelaic acid [53]. Peak (49) [tR 3.165 min, (M-H)^−^ at *m/z* 173.0449, (C_7_H_9_O_5_)^−^] displayed a characteristic fragment ion peak at *m/z* 111.0439 [M-H-COO-H_2_O]^−^ and a fragment ion at *m/z* 93.0339 [M-H-COO-2H_2_O]^−^ as a base peak which suggests the decarboxylation and successive dehydration of the molecular ion peak. Thus, peak (49) was assigned as shikimic acid [38]. Peak (67) [tR 5.049 min, (M-H)^−^ at *m/z* 116.9278, (C_4_H_5_O_4_)^−^] displayed a characteristic base peak at *m/z* 99.9253 [M-H-H_2_O]^−^. Thus, peak (67) was assigned as succinic acid [61].

From the previous literature, it has been recognized that the only pyrogallol (peak 6) was previously identified from *V. negundo* leaves and stem [39]. Meanwhile, fumaric acid (peak 1), tartaric acid (peak 2), azelaic acid (peak 42), shikimic acid (peak 49) and succinic acid (peak 67) were recognized for the first time in the genus *Vitex*.

### Identification of phenolic acid derivatives

Phenolic acids and their derivatives are one of the most abundant classes of phytochemical metabolites, which account for about a third of all polyphenolic peaks [65]. They are organic acids that basically contain a carboxyl group, hydroxyl or methoxy substituent attached directly to an aromatic ring in their chemical structure [66]. Phenolic acids are classified into benzoic acid derivatives and cinnamic acid derivatives [67]. In this study, a total of 39 metabolites in the total defatted methanolic extract, 28 metabolites in the ethyl acetate fraction, and 29 metabolites in the *n*-butanol fraction were tentatively identified as phenolic acids and/or their derivatives.

### Benzoic acid derivatives

A total of thirteen benzoic acid derivatives (peaks 5, 8, 9, 10,, 13, 17, 18, 19, 20, 23, 24, and 26) were tentatively identified as either phenolic acids or phenolic acid glycosides from *V. pubescens* bark. Phenolic acids commonly display a characteristic loss of COO (-44 Da) either in negative or positive ionization modes. Besides, the fragmentation pattern of phenolic acid glycosides could be explained through the loss of intact sugar residue resulting in a base peak fragment ion corresponding to the aglycone part [68].

Peak (5) [tR 0.586 min, (M-H)^-^ at *m/z* 169.0133 (C_7_H_5_O_5_)^-^] exhibited a fragment ion at *m/z* 125.0236 [M-H-COO]^-^ as the base peak which suggests the decarboxylation of the molecular ion (-44 Da). Therefore, peak (5) was tentatively identified as gallic acid [38]. Peak (9) [tR 0.700 min, (M-H)^-^ at *m/z* 167.0341 (C_8_H_7_O_4_)^-^] showed a base peak fragment ion at *m/z* 152.0110 [M-H-CH_3_]^-^ indicating the loss of one methyl group (-15 Da) along with an intense fragment ion at *m/z* 108.0221[M-H-CH_3_-COO]^-^ representing successive decarboxylation of the base peak ion. Therefore, peak (9) was tentatively identified as hydroxy-methoxy benzoic acid [69]. Peak (10) [tR 0.712 min, (M-H)^-^ at *m/z* 153.0185 (C_7_H_5_O_4_)^-^] showed a characteristic base peak at *m/z* 109.0295 [M-H-COO]^-^. Therefore, peak (10) was tentatively identified as protocatechuic acid [36]. Peak (17) [tR 1.197 min, (M-H)^-^ at *m/z* 183.0291 (C_8_H_7_O_5_)^-^)] showed a characteristic fragment at *m/z* 168.0064 [M-H-CH_3_]^-^ representing the loss of a methyl group and yielding a gallic acid residue [gallic acid-H]^-^. Therefore, peak (17) was assigned as methyl gallate [46]. Peak (18) [tR 1.226 min, (M + H)^+^ at *m/z* 139.0383 (C_7_H_7_O_3_)^+^] displayed an intense fragment ion at *m/z* 121.0281 [M + H − H_2_O]^+^ and a base peak ion at *m/z* 95.0485 [M + H − COO]^+^.Therefore, peak (18) was tentatively identified as protocatechualdehyde [47]. Peaks (19) [tR 1.252 min, (M-H)^-^ at *m/z* 629.2085] and (20) [tR 1.297 min, (M-H)^-^ at *m/z* 137.0237 (C_7_H_5_O_3_)^-^ and (M + H)^+^ at *m/z* 139.0382 (C_7_H_7_O_3_)^+^] both showed an intense fragment ion at *m/z* 93.0343 representing the decarboxylation of peak (20) molecular ion [M-H-COO]. Peak (19) showed a fragment ion at *m/z* 137.0242 as the base peak. Thus, peaks (19) and (20) were assigned as *p*-hydroxybenzoic acid derivative [40] and *p*-hydroxybenzoic acid [38]; respectively. Peak (23) [tR 1.532 min, (M-H)^-^ at *m/z* 152.0109 (C_8_H_8_O_3_)^-^] showed an indicative fragment ion at *m/z* 137.0236 [M-H-CH_3_]^-^ representing *p*-hydroxybenzoic acid moiety also demethylation (-15 Da) of molecular ion peak and a base peak fragment ion at *m/z* 108.0214 [M-H-COO]^-^. Therefore, peak (23) was identified as methyl hydroxybenzoic acid [70, 71]. Peak (24) [tR1.535 min, (M-H)^-^ at *m/z* 167.0341 (C_8_H_7_O_4_)^-^] showed characteristic fragments at *m/z* 152.0118[M-H-CH_3_]^-^ and *m/z* 123.0448 [M-H-COO]^-^ indicating the demethylation (-15 Da) and decarboxylation (-44 Da) of the molecular ion peak, respectively. Moreover, an intense fragment at *m/z* 108.0221[M-H-CH_3_-COO]^-^ as the base peak. Therefore, peak (24) was tentatively identified as vanillic acid [72]. Peak (26) [tR 1.784 min, (M-H)^-^ at *m/z* 197.0441 (C_9_H_9_O_5_)^-^] exhibited an intense fragment ion at *m/z* 167.0002[M-H-2CH_3_]^-^ that indicates the loss of two methyl residues, besides a base peak ion at *m/z* 123.0084 [M-H-2CH_3_-COO]. Therefore, peak (26) was tentatively identified as syringic acid [38]. Phenolic acid glycosides were also annotated at peak (8) [tR 0.679 min, (M-H)^-^ at *m/z* 299.0764 (C_13_H_15_O_8_)^-^] and peak (13) [tR0.818 min, (M-H)^-^ at *m/z* 329.0875 (C_14_H_17_O_9_)^-^ showing characteristic fragment ions at *m/z* 137.0240 and *m/z* 167.0348; respectively corresponding to [M-H-hexoside]^-^ revealing the natural cleavage of intact hexoside residue (-162 Da) attached *via O*-glycosylation to yield the aglycone parts which are [hydroxybenzoic acid-H]^-^ and [vanillic acid-H]^-^. Therefore, peak (8) and peak (13) were tentatively identified as *p*-hydroxybenzoic acid-*O*-hexoside [38] and vanillic acid-*O*-hexoside [38].

### Cinnamic acid derivatives

Two peaks **(**15) and (48) were classified as cinnamic acid derivatives which were tentatively identified as *p*-coumaric acid and caffeic acid. Peak (15) [tR 0.962 min, (M-H)^−^ at *m/z* 163.0404 (C_9_H_7_O_3_)^−^] showed a characteristic intense fragment at *m/z* 135.0444 [M-H-CO]^−^ representing the loss of CO^−^ group (-28 Da) also the base peak ion at *m/z* 93.0346 [M-H-COO-C_2_H_2_]^−^. Therefore, peak (15) was tentatively identified as *p*-coumaric acid [44]. Peak (48) [tR 3.081 min, (M-H)^−^ at *m/z* 179.0344 (C_9_H_7_O_4_)^−^ and (M + H)^+^ at *m/z*181.0121 (C_9_H_9_O_4_)+] showed a characteristic base peak at *m/z* 135.0441 [M-H-COO]^−^ and *m/z* 117.0346 [M-H-COO-H_2_O]^−^. Therefore, peak (48) was tentatively identified as caffeic acid [38].

Phenolic acid derivatives identified herein for the first time in genus *Vitex* include gallic acid (peak 5), *p*-hydroxybenzoic acid-*O*-hexoside (peak 8), pyrocatechol (peak 11), vanillic acid-*O*-hexoside (peak 13), methyl gallate (peak 17), methyl hydroxybenzoic acid (Peak 23) and syringic acid (peak 26). Besides, some phenolic acids were previously reported in different *Vitex* species, viz. hydroxy-methoxy benzoic acid (peak 9) [69], protocatechuic acid (peak 10) [36], protocatechualdehyde (peak 18) [39], *p*-hydroxybenzoic acid derivative (peak 19) [40], *p*-hydroxybenzoic acid (peak 20) [36, 37], vanillic acid (peak 24) [69], *p*-coumaric acid (peak 15) [55] and caffeic acid (peak 48) [40, 73, 74].

### Acyl quinic acid derivatives

In the current study, quinic acid and quinic acid derivatives were annotated in *V. pubescens* bark. Peak (4) [ tR 0.470 min, (M-H)^−^ at *m/z* 533.1719] was tentatively identified as quinic acid derivative, besides, peak (12) [tR 0.769 min, (M-H)^−^ at *m/z* 191.0553 (C_7_H_11_O_6)_^−^] was identified as quinic acid. The common fragmentation pathway of quinic acid and its derivatives showed the ion at *m/z* 191 as a parent peak, indicating the presence of quinic acid moiety in the negative ion mode [quinic acid-H]^−^. Also, the intense fragment ion corresponding to the base peak at *m/z* 173.0450 [M-H-H_2_O]^−^indicating the loss of a water molecule [42]. Predominantly, quinic acid conjugates with esterified acyl moieties to form di- or tri-acyl quinic acid derivatives were annotated herein. Fourteen acyl quinic acid derivatives (peaks 7, 14, 16, 22, 43, 46, 47, 50, 51, 53, 54, 55, 59, and 60) were tentatively identified in the *V. pubescens* bark.

Peak (7) [tR 0.665 min, (M-H)^-^ at *m/z* 311.0767, (C_14_H_15_O_8_)^-^] exhibited a characteristic fragment ion at *m/z* 191.0553 [quinic acid-H]^-^ and a base peak ion at *m/z* 137.0242 [hydroxybenzoic acid-H]^-^ also an intense peak at *m/z* 93.0350 [hydroxybenzoic acid-H-COO]^-^ in the MS/MS spectrum .Therefore, peak (7) was tentatively identified as *O*-*p*-hydroxybenzoyl quinic acid [40]. The isomeric peaks (14) [tR 0.831 min, (M-H)^-^ at *m/z* 353.0873 (C_16_H_17_O_9_)^-^] and (22) [tR0.831 min, (M-H)^-^ at *m/z* 353.0873 (C_16_H_17_O_9_)^-^] exhibited a base peak fragment ion at *m/z* 191.0562 [quinic acid-H]^-^, besides, an intense fragment ion at *m/z* 179.0344 [caffeic acid-H]^-^ could be noticed in case of peak (14), while only a small undetectable fragment ion in peak (22) could be observed along with the presence of a fragment ion at *m/z* 135.0448 [caffeic acid-H-COO]^-^ [43]. According to the literature [75], the high intensity of caffeic acid fragment ion at *m/z* 179 was used to distinguish between different isomers of caffeoylquinic acid. Therefore, peaks (14) and (22) were tentatively identified as 3-*O*-caffeoylquinic acid (neochlorogenic acid) and 5-*O*-caffeoylquinic acid (chlorogenic acid), respectively. Peak (16) [tR 1.194 min, (M-H)^-^ at *m/z* 341.0881 (C_15_H_16_O_9_)^-^] showed characteristic fragments of quinic acid at *m/z* 191.0562 as base peak, *m/z* 173.0445 [quinic acid-H-H_2_O]^-^ as well as fragment ion at *m/z* 167.0339 attributed to the presence of vanilloyl residue [vanillic acid-H]^-^ and *m/z* 152.0084 [vanillic acid-H-CH_3_]. Thus, peak (16) was tentatively identified as *O*-vanilloylquinic acid [45]. Peaks (43) [tR 2.934 min, (M-H)^-^ at *m/z* 533.1293 (C_25_H_25_O_13_)^-^], (47) [tR3.055 min, (M-H)^-^ at *m/z* 515.1194 (C_25_H_23_O_12_)^-^], (50) [tR 3.192 min, (M-H)^-^ at *m/z* 473.1085 (C_20_H_25_O_13_)^-^], (51) [tR 3.221 min, (M-H)^-^ at *m/z* 515.1194 (C_25_H_23_O_12_)^-^], (53) [tR 3.329 min, (M-H)^-^ at *m/z* 499.1247 (C_22_H_27_O_13_)^-^], (54) [tR 3.449 min, (M-H)^-^ at *m/z* 529.1353 (C_26_H_25_O_12_)^-^], (59) [tR 3.633 min, (2 M-H)^-^ at *m/z* 707.1985 (C_32_H_36_O_18_)^-^] and (60) [tR 3.644 min, (M-H)^-^ at *m/z* 543.1507 (C_27_H_27_O_12_)^-^] were characterized with abundant fragment ion at *m/z* 353 which relay on the presence caffeoyl quinic acid moiety conjugated with other indicative phenolic acid fragments in acylated form [75]. Therefore, these peaks were tentatively identified as *O*-caffeoyl-*O*-syringoylquinic acid (peak 43) [45], di-*O*-caffeoylquinic acid (peak 47) [42], *O*-*p*-hydroxybenzoyl-*O*-caffeoylquinic acid (peak 50) [76], di-*O*-caffeoylquinic acid isomer (peak 51) [41], *O*-*p*‐coumaroyl‐*O*‐caffeoylquinic acid (peak 53) [77], methyl-dicaffeoyl quinate (peak 54) [41], *O*-caffeoylquinic acid dimer (peak 59) [37] and ethyl-di caffeoyl quinate (peak 60) [41]; respectively.

Therefore, the present study explored promising acyl quinic acid derivatives, including eight newly identified metabolites in genus *Vitex, viz. O*-vanilloylquinic acid (peak 16), *O*-caffeoyl-*O*-syringoylquinic acid (peak 43), *O*-caffeoyl-*O*-vanilloylquinic acid (peak 46), *O*-*p*-hydroxybenzoyl-*O*-caffeoylquinic acid (peak 50), *O*-*p*‐coumaroyl‐*O*‐caffeoylquinic acid (peak 53), methyl-dicaffeoyl quinate (peak 54), dicaffeoylshikimic acid (peak 55) and ethyl-di-caffeoyl quinate (peak 60). As well as, eight quinic acid derivatives were previously reported from different *Vitex* species, viz. quinic acid derivative (peak 4) [37], *O*-*p*-hydroxy benzoyl quinic acid (peak 7) [40], quinic acid (peak 12) [36], neochlorogenic acid (peak 14) [55], chlorogenic acid (peak 22) [55], di-*O*-caffeoylquinic acid (peak 47) [49], di-*O*-caffeoylquinic acid isomer (peak 51) [37], and *O*-caffeoylquinic acid dimer (peak 59) [37].

### Identification of flavonoid derivatives

Flavonoids are mainly composed of three-ring diphenyl propane (C_6_C_3_C_6_) [78]. They would present either in aglycone or mostly in glycoside form attached to sugar moiety through a hydroxyl group (flavonoid-*O*-glycosides) or the anomeric carbon of sugar part attached directly to aglycone part commonly *C*-6 or *C*-8 position (flavonoid-*C*-glycosides) [51].

LC-ESI-MS/MS fragmentation patterns would help in identifying the nature and position of sugar attachment in *O*- and *C*-glycosides. The fragmentation of *O*-glycosides would be easily characterized by the loss of the sugar moiety through cleavage of glycosidic bond yielding the aglycone and sugar parts as product ions. The loss of *O*-sugar moiety *viz. O*-hexoside, *O*-pentoside, *O*-deoxyhexoside would be revealed through the loss of 162, 132 and 146 Da, respectively [51]. Meanwhile, *C*- glycosides would show interglycosidic cleavage of the sugar part [51]. The diagnostic fragmentation pathway of *C*-glycosides commonly includes the loss of water (-18 Da) besides the cross- ring cleavages ^0–2^X_º_ [(O-C1 and C2-C3)] and ^0–3^X_º_ [(O-C1 and C3-C4)] of sugar units. Hence, the fragmentation of *C*-glycosides showed *C*-hexosides (X_ºH_) [M-120/90]^+/−^, *C*- deoxyhexosides (X_ºdH_) [M-104/74]^+/−^ and *C*-pentosides (X_ºP_) [M-90/60]^+/−^ [33]. In the present study of *V. pubescens* bark, mainly the flavone class was dominant. The identified metabolites included three main aglycones viz. luteolin, apigenin and chrysoeriol with their derivatives. Predominantly, the identified peaks were flavonoid-*C*-glycosides either mono-*C*-glycosides, di-*C*-glycosides or their derivatives which showed inter glycosidic cleavage of sugar part as the common fragmentation pattern as explained previously. The dominant fragmentation pattern in the case of mono-*C*-glycosides was [Ag + 41/71]^+/−^ and di-*C*-glycosides were [Ag + 83/113]^+/−^ representing the aglycone part (Ag) plus the remaining parts of the linked sugars [51].

### Luteolin derivatives

Thirteen peaks were tentatively identified as luteolin derivatives (peaks 25, 28, 30, 31, 32, 33, 34, 35, 39, 40, 41, 57, and 62) in *V. pubescens* bark. The observed diagnostic fragments for luteolin-mono- or di-*C*-glycosides were [327/357]^−^ and [369/399]^−^; respectively besides the presence of luteolin aglycone [Ag]^−^ fragment at *m/z* 285 [luteolin-H]^−^ [49]. Peak (57) displayed the fragmentation pathway of luteolin aglycone as [tR3.572 min, (M-H)^−^ at *m/z* 285.0397 (C_15_H_9_O_6_)^−^ and (M + H)^+^ at *m/z* 287.0542 (C_15_H_11_O_6_)^+^] showing characteristic fragment ions at *m/z* 151.0035 [M-H-C_8_H_6_O_2_]^−^ and 133.0294[M-H-C_7_H_4_O_4_]^−^. It was supposed that the two fragment ions were produced through cross-ring cleavage of ring B of luteolin aglycone [57].

### Luteolin mono-*C*-glycoside

Peak (34) [tR 2.503 min, (M-H)^−^ at *m/z* (2 M-H)^−^ at *m/z* 895.1937 (C_42_H_38_O_22_)^−^] as well as the isomeric peaks (35) [tR 2.799 min, (M-H)^−^ at *m/z* 447.0930 (C_21_H_19_O_11_)^−^ and (M + H)^+^ at *m/z* 449.1076 (C_21_H_21_O_11_)^+^] and (62) [tR 3.196 min, (M-H)^−^ at *m/z* 447.0930 (C_21_H_19_O_11_)^−^ and (M + H)^+^ at *m/z* 449.1076 (C_21_H_21_O_11_)^+^] displayed the common fragmentation pattern of luteolin-mono-*C*-glycoside exhibiting indicative fragments at *m/z* 357.0616 [M-H-90]^−^[Ag + 71]^−^ and *m/z* 327.0511[M-H-120]^−^ [Ag + 41]^−^ as base peak indicating internal cleavage of ^0–3^ X_ºH_ and ^0–2^ X_ºH_ of *C*-hexose residue attached to the aglycone part. Therefore, peak (34) was annotated as luteolin-*C*-hexoside dimer [37] and the isomeric peaks (35) and (62) were tentatively identified as luteolin-*C*-hexoside but the position of sugar moiety remains unclear. According to Ferreres et al., [M-H-90]^−^ fragment ion is more abundant in 6-*C*-hexosyl than 8-*C*-hexosyl luteolin, furthermore, the elution of 8-*C*-hexosyl occurs before 6-*C*-hexosyl [49]. Therefore, peak (35) was tentatively identified as luteolin-8-*C*-hexoside (orientin) and peak (62) was identified as luteolin-6-*C*-hexoside (isoorientin). Peak (40) [tR 2.799 min, (M-H)^−^ at *m/z* 461.1086 (C_22_H_21_O_11)_^−^] showed fragment ions at *m/z* 429.0835 [M-H- OCH_3_]^−^ representing the cleavage of a methoxy group (-32Da), a fragment ion at *m/z* 371.0774 [M-H-90]^−^ and an intense peak at *m/z* 341.0670 [M-H-120]^−^ indicating the internal cleavage ^0–3^ X_ºH_ and ^0–2^ X_ºH_ of hexose residue; respectively. Therefore, peak (40) was tentatively identified as methoxy luteolin-*C*-hexoside [52].

### Luteolin di-*C/O*-glycoside

Peak (25) [tR 1.649 min, (M-H)^−^ at *m/z* 609.1455 (C_27_H_28_O_16_)^−^ and (M + H)^+^ at *m/z* 611.1612 (C_27_H_30_O_16_)^+^], peak (30) [tR 2.208 min, (M-H)^−^ at *m/z* 609.1455 (C_27_H_28_O_16_)^−^ and (M + H)^+^ at *m/z* 611.1612 (C_27_H_30_O_16_)^+^], peak (31) [tR 2.257 min, (M-H)^−^ at *m/z* 579.1353 (C_26_H_26_O_15_)^−^] and peak (32) [tR 2.353, (M-H)^−^ at *m/z* 593.1510 (C_27_H_29_O_15_)^−^ and (M + H)^+^ at *m/z* 595.1645 (C_27_H_31_O_15_)^+^] exhibited the common fragmentation pattern of luteolin-di-*C*-glycoside i.e.[Ag + 83/113]^+/−^ corresponding to [369/399]^+/−^ fragments. Peak (25) displayed MS/MS spectrum showing significant peaks at *m/z* 519.1132 [M-H-90]^−^ and *m/z* 489.1039 [M-H-120]^−^ produced by ^0–3^ X_ºH_ and ^0–2^ X_ºH_ cleavage of *C*-hexose residue; respectively, besides the indicative peaks at *m/z* 399.0726 [Ag + 113]^−^ and m*/z* 369.0619 [Ag + 83]^−^ as base peak. Therefore, peak (25) was tentatively identified as luteolin-di-*C*-hexoside [49]. Furthermore, peak (30) exhibited the same molecular ion of peak (25) as well as displaying fragment ions at *m/z* 519.1132 [M-H-90]^−^ and *m/z* 489.1039 [M-H-120]^−^ along with [357/327]^−^ fragments which exclusively indicate the presence of mono-*C*-hexoside. Besides, the fragment ion at *m/z* 447.0935 [M-H-162]^−^ indicates the natural loss of terminal *O*-hexoside moiety. Therefore, peak (30) was tentatively identified as luteolin*-C*-hexoside-*O*-hexoside [49, 52]. Peak (31) displayed characteristic fragment ions at *m/z* 519.1144 [M-H-60]^−^ and *m/z* 489.1037 [M-H-90]^−^ represent the cross-link cleavage ^0–3^ X_º P_ and ^0–2^ X_º P_ of *C*- pentose moiety; respectively as well as a fragment ion at *m/z* 459.0933 [M-H-120]^−^ representing interglycosidic cleavage (^0–2^ X_º H_) of *C*-hexose moiety. Consequently, the fragmentation pattern indicates the presence of luteolin-di-*C*-glycosides which was confirmed by the existence of the base peak fragment ion at *m/z* 399.0723 [Ag + 113]^−^ and an intense fragment at *m/z* 369.0616 [Ag + 83]^−^. Therefore, peak (31) was tentatively identified as luteolin-*C*-pentoside-*C-*hexoside (Ferreres et al., 2017). Peak (32) showed fragment ions at *m/z* 503.1191 [M-H-90]^−^ and 473.1089 [M-H-120]^−^ the cross-link cleavage of *C*-hexose moiety. Also, characteristic peaks at *m/z* 357.0619 [ M-H-146-90]^−^ [Y_º_+71]^−^, *m/z* 327.0510 [ M-H-146-120]^−^[Y_º_+41]^−^ as base peak also an intense fragment ion at *m/z* 309.0404 [ M-H-146-120-18]^−^ indicate the terminal cleavage of *O*-deoxyhexoside moiety (-146 Da) with internal cleavage of hexoside moiety attached to the aglycone part with *C*-glycosidic bond. Therefore, peak (32) was tentatively identified as luteolin-*C*-(*O*-deoxyhexosyl)hexoside [49]. Peak (33) [tR 2.480, (M-H)^−^ at *m/z* 579.1353 (C_26_H_26_O_15_)^−^ and (M + H)^+^ at *m/z* 581.1504 (C_26_H_28_O_15_)^+^] exhibited characteristic fragment ions at *m/z* 489.1003[M-H-90]^−^ and *m/z* 459.0924 [M-H-120]^−^. Besides, the differential ions at *m/z* 357.0619 [M-H-132-90]^−^ [Ag + 71]^−^, *m/z* 327.0510 [M-H-132-120]^−^ [Ag + 41]^−^ as base peak and an intense peak at *m/z* 309.0404 [M-H-132-120-18]^−^ [Ag + 41 − 18]^−^ which are considered as diagnostic fragment ions for luteolin-mono-*C*-hexoside and suggesting the terminal cleavage of *O*-pentoside moiety alongside with internal fragmentation of *C*-hexoside moiety. Therefore, peak (33) was tentatively identified as luteolin*-C*-(*O*-pentosyl)hexoside [49]. Peak (39) displayed the same fragmentation pathway of peak (33) besides the presence of a characteristic fragment ion at *m/z* 167.0345 representing the *O*-vanilloyl moiety. Therefore, peak (39) was tentatively identified as vanilloyl-*C*-(*O*-pentosyl)hexosyl luteolin [49]. Peak (41) was tentatively identified as luteolin-*O*-(*O*-deoxyhexosyl)hexoside [42].

### Luteolin tri-glycosides

Peak (28) [tR2.150 min, (M-H)^-^ at *m/z* 755.2029 (C_33_H_38_O_20_)^-^] exhibited fragment ions at *m/z* 635.1636 [M-H-120]^-^ indicating the cleavage of ^0–2^ X_ºH_ of a *C*- hexose residue, *m/z* 593.1502 [M-H-162]^-^ indicating the natural loss of *O*-hexoside residue, *m/z* 519.1117 [M-H-146-90]^-^ corresponding to the natural loss of *O*-deoxyhexoside moiety (-146 Da) and ^0–3^ X_ºH_ cleavage of *C*-hexosyl moiety. Besides, the indicative fragment ions at *m/z* [357/327]^-^ for mono-*C*-luteolin glycoside. Therefore, peak (28) was tentatively identified as luteolin-*C*-hexoside-*O*-hexoside-*O*-deoxyhexoside.

Interestingly, the present study introduced various luteolin-*C*-glycosides and their derivatives, including three compounds identified in genus *Vitex* for the first time, viz. luteolin-*C*-hexoside-*O*-hexoside-*O*-deoxyhexoside (peak 28), methoxy luteolin-*C*-hexoside (peak 40) and luteolin-*O*-(*O*-deoxyhexosyl)hexoside (peak 41). In addition to previously isolated or identified compounds from various *Vitex* species, viz. luteolin-di-*C*-hexoside (peak 25) [49], luteolin-*C*-hexoside-*O*-hexoside (peak 30) [49], luteolin-*C*-pentoside-*C-*hexoside (peak 31) [49], luteolin-*C*-(*O*-deoxyhexosyl) hexoside (peak 32) [49], luteolin*-C*-(*O*-pentosyl)hexoside (peak 33) [49], luteolin-*C*-hexoside dimer (peak 34) [37], luteolin-8-*C*-hexoside (orientin) [49] (peak 35), vanilloyl-*C*-(*O*-pentosyl)hexosyl luteolin (peak 39) [49], luteolin (peak 57) [40] and luteolin-6-*C*-hexoside (isoorientin) (peak 62) [49].

### Apigenin derivatives

Six peaks were tentatively identified as apigenin derivatives **(**peaks 27, 29, 36, 38, 45, and 52) from *V. pubescens* bark and were classified as apigenin-*C*-glycosides with ^0–3^ X_º_ and ^0–2^ X_º_ cleavage of the sugar moiety as previously mentioned. According to Farag et al., fragment ions at *m/z* [311/341]^−^ and [353/383]^−^ were regarded as diagnostic fragments for the apigenin-mono-*C*-glycoside [Ag + 41/81]^−^ or apigenin-di-*C*-glycoside [Ag + 83/113]^−^, respectively [33] .

### Apigenin-mono-*C* -glycosides

The isomeric peaks (38) [tR 2.750, (M-H)^-^ 431.0983 (C_21_H_19_O_10_)^-^ and (M + H)^+^ at *m/z* 433.1124 (C_21_H_21_O_10_)^-^] and (52) [tR2.750, (M-H)^-^ 431.0983 (C_21_H_19_O_10_)^-^ and (M + H)^+^ at *m/z* 433.1124 (C_21_H_21_O_10_)^-^], showed distinctive fragment ions the MS/MS spectrum at *m/z* 341.0662 [M-H-90]^-^ [Ag + 81]^-^ and *m/z* 311.0555 [M-H-120]^-^ [Ag + 41]^-^ as base peak indicating the presence of apigenin-mono-*C*-glycoside, besides the internal ^0–3^ X_ºH_ and ^0–2^ X_ºH_ cleavage of the hexose moiety as well as the fragment ion at *m/z* 269.0452 corresponding to apigenin aglycone [Ag]^-^. Thus, these peaks were tentatively identified as apigenin-*C*-hexoside. The elution of 8-*C*-hexoside usually occurs before 6-*C*-hexoside [49], consequently, peak (38) and peak (52) were tentatively identified as apigenin-8-*C*-hexoside (vitexin) and apigenin-6-*C*-hexoside (isovitexin), respectively [36, 49].

### Apigenin-di-C/O glycosides

Peak (27) [tR 2.081 min, (M-H)^−^ at *m/z* 593.1510 (C_27_H_28_O_15_)^−^ and (M + H)^+^*m/z* 595.1645 (C_27_H_30_O_15_)^+^] exhibited fragment ions at *m/z* 383.0775 [M-H-120-90]^−^ [Ag + 113]^−^and a base peak at *m/z* 353.0668 [M-H-120-120]^−^ [Ag + 83]^−^ indicating the presence of a di-*C*-glycoside as well as fragment ions at *m/z* 503.1165[M-H-90]^−^, *m/z* 473.1091[M-H-120]^−^ produced by ^0–3^ X_ºH_ and ^0–2^ X_º H_ cleavage of the *C*-hexose residue; respectively. Therefore, peak (27) was tentatively identified as apigenin-di-*C*-hexoside [49]. Similarly, peak (29) [tR 2.206 min, (M-H)^−^ at *m/z* 563.1742 (C_26_H_26_O_14_)^−^] showed intense peaks at *m/z* [383.0772/353.0660]^−^ which confirmed the existence of apigenin-di-*C*-glycoside in addition to fragment ions at *m/z* 503.1142 [M-H-60]^−^ and *m/z* 473.1071 [M-H-90]^−^ representing ^0–3^X_ºP_ and ^0–2^X_ºP_ cleavage of a *C*-pentose moiety, respectively, along with a fragment ion at *m/z* 443.0971 [M-H-120]^−^ indicating ^0–2^X_ºH_ cleavage of *C*-hexose residue. Therefore, peak (29) was tentatively identified as apigenin-*C*-hexoside-*C*-pentoside [49]. Peak (36) [tR 2.685 min, (M-H)^−^ at *m/z* 577.1564 (C_27_H_29_O_14_)^−^ and (M + H)^+^*m/z* 579.1702 (C_27_H_31_O_14_)^+^], the MS/MS spectrum showed characteristic peaks at *m/z* 487.1249 [M-H-90]^−^, *m/z* 457.1139 [M-H-120]^−^ besides, the fragment ion at *m/z* 431.0958 [M-H-146]^−^ indicating the cleavage of *O*-deoxyhexose radical. Additionally, the presence of fragment ions at *m/z* [341.0662/ 311.056]^−^ confirmed the presence of apigenin mono-*C-*glycoside. Therefore, Peak (36) was tentatively identified as apigenin-*C*-(*O*-deoxyhexosyl)hexoside [49]. Peak (45) [tR 2.987 min, (M-H)^−^ at *m/z* 713.1729 (C_34_H_32_O_18_)^−^] showed the fragment ion at *m/z* 593.1327 [M-H-120]^−^ representing the internal cleavage of ^0–2^ X_ºH_ of a *C*-hexose moiety. Besides, the diagnostic fragment ions at *m/z* 341.0664 [M-H-150-132-90]^−^ [Ag + 81]^−^, an intense peak at *m/z* 311.0556 [M-H-150-132-120]^−^ [Ag + 41]^−^ and *m/z* 293.0450[M-H-150-132-120-18]^−^ [Ag + 41 − 18]^−^ as a base peak indicating the presence of acylated apigenin mono-*C*-glycoside which is hexoside moiety along with terminal loss of a pentoside (-132 Da) and a vanilloyl moiety. Therefore, peak (45) was tentatively identified as vanilloyl-*C*-(*O*-pentosyl)hexosyl apigenin [49].

Therefore, the present study resulted in the identification of six apigenin-*C*-glycosides and their derivatives which were previously isolated or identified from various *Vitex* species [36, 49], viz. apigenin-di-*C*-hexoside (peak 27), apigenin-*C*-hexoside-*C*-pentoside (peak 29), apigenin-*C*-(*O*-deoxyhexosyl) hexoside (peak 36), apigenin-8-*C*-hexoside (vitexin) (peak 38), vanilloyl-*C*-(*O*-pentosyl)hexosyl apigenin (peak45) and apigenin-6-*C*-hexoside (isovitexin) (peak 52).

### Chrysoeriol derivatives

Peak (37) [tR 2.745 min, (M-H)^-^ at *m/z* 607.1699 (C_28_H_31_O_15_)^-^] showed fragment ions at *m/z* 517.0843 [M-H-90]^-^, *m/z* 487.1267[M-H-120]^-^ corresponding to ^0–3^X_ºH_ and ^0–2^X_ºH_ cleavage of *C*-hexose moiety, also, a fragment ion at *m/z* 461.1070 [M-H-146]^-^ indicated the cleavage of a deoxyhexosyl radical along with the presence of an ion at *m/z* 299.0618 corresponding to chrysoeriol as the aglycone part [Ag]^-^. Therefore, peak (37) was tentatively identified as chrysoeriol-*C*-(*O*-deoxyhexosyl) hexoside [49]. Peak (44) [tR2.948 min, (M-H)^-^ at *m/z* 743.1830 (C_35_H_34_O_18_)^-^] exhibited the same fragmentation pathway as peak (45); meanwhile, the main difference was the presence of a chrysoeriol peak at *m/z* 299.0618 instead of apigenin peak. Therefore, peak (44) was tentatively identified as vanilloyl-6-*C*-(*O*-pentosyl)hexosyl chrysoeriol [49]. Both peaks (37) and (44) were previously identified in *Vitex peduncularis* bark [49].

### Other polyphenolics

Peak (6) [tR 0.586 min, (M-H)^−^ at *m/z* 125.0235, (C_6_H_5_O_3_)^−^] displayed a base peak fragment ion at *m/z* 79.0181 [M-H-CO-H_2_O]^−^. Thus, peak (6) was assigned as pyrogallol [39]. Peak (11) [tR 0.739 min, (M-H)^−^ at *m/z* 109.0289 (C_6_H_5_O_2_)^−^] showed characteristic peaks at *m/z* 91.0189 [M-H-H_2_O]^−^, *m/z* 81.0349 [M-H-CO]^−^ and peak at *m/z* 65.0034 [M-H-COO]^−^. Therefore, peak (11) was tentatively identified as pyrocatechol [32]. Peak (21) [tR1.384 min, (M + H)^+^ at *m/z* 163.0383 (C_9_H_8_O_3_)^+^] exhibited characteristic fragments at *m/z* 135.0439 [M + H-CO]^+^, 117.0334 [M + H-CO-H_2_O] ^+^, 107.0487[M + H-2CO]^+^ and 89.0386 [M + H-2CO-H_2_O] ^+^. Therefore, peak (21) was tentatively identified as umbelliferone [48]. Interestingly, this is the first time to report its identification in genus *Vitex*.

### Triterpenoids

Peak (56) [tR 3.509 min, (M-H)^-^ at *m/z* 501.3216 (C_30_H_45_O_6_)^-^] showed the fragmentation pathway of triterpenes characterized with the loss of water molecule at *m/z* 483.3102 [M-H-H_2_O]^-^, a characteristic intense peak at *m/z* 455.3157 [M-2 H-COOH]^-^ indicating decarboxylation (-46 Da), also, an ion at *m/z* 439.3218 [M-H-COO-H_2_O]^-^. Therefore, peak (56) was tentatively identified as pomaceic acid [58]. Peak (63) [tR3.963 min, (M + H)^+^ at *m/z* 473.3244 (C_30_H_49_O_4_)^+^ showed characteristic fragment ions at *m/z* 455.3216 [M + H-H_2_O]^+^ and *m/z* 427.2074 [M + H-COOH]^+^, also, a fragment ion at *m/z* 413.2340 [M + H-COO-CH_4_]^+^ indicating the cleavage of a water and a ketene molecule, besides, a characteristic base peak ion at *m/z* 409.3064 [M + H-HCOOH-H_2_O]^+^ indicating decarboxylation and dehydration (-64 Da) of the molecular ion. Therefore, peak (63) was tentatively identified as maslinic acid [58]. Peak (64) [tR4.460 min, (M-H)^-^ at *m/z* 487.3424 (C_30_H_47_O_5_)^-^] exhibited a fragment ion at *m/z* 469.3328 [M-H-18]^-^ as the base peak, also, fragment ions at *m/z* 443.3533 [M-H-COO]^-^, *m/z* 425.3421 [M-H-COO-H_2_O]^-^, 407.3317 [M-H-COO-2H_2_O]^-^ indicating decarboxylation and successive dehydration Therefore, peak (64) was tentatively identified as euscaphic acid [59]. Peak (66) [tR4923 min, (M + H)^+^ at *m/z* 457.3295 (C_30_H_49_O_3_)^+^] exhibited fragment ions at *m/z* 439.3149 [M + H- H_2_O]^+^, *m/z* 411.3270 [M + 2 H-COO]^+^ and the base peak at *m/z* 393.3122 [M + 2 H-COO-H_2_O]^+^ suggesting the successive loss of carboxylic acid (-46 Da) and water molecule (-18 Da). Therefore, peak (66) was tentatively identified as ursolic acid [58]. Peak (68) [tR 5.104 min, (M-H)^-^ at *m/z* 471.3473) exhibited the characteristic fragments at *m/z* 453.3365 [M-H-H_2_O]^-^ and *m/z* 411.0197 [M-H-COO-CH_4_]^-^ attributed to decarboxylation and cleavage of a ketene molecule along with a related fragment ion at *m/z* 409.0375 [M-H-C_2_H_6_O_2_]^-^ and an intense peak at *m/z* 407.3304 [M-H-CH_4_O_3_]^-^, and a fragment ion at *m/z* 390.9869 [M-H-COO-2H_2_O]^-^. Therefore, peak (68) was tentatively identified as pomolic acid [58]. Peak (71) [tR5.990 min, (M + H)^+^ at *m/z* 457.3618 (C_30_H_49_O_3_)^+^] showed fragment ions at *m/z* 439.3587 [M + H-H_2_O]^+^ and *m/z* 411.359 [M + H- HCOOH]^+^ corresponding to the loss of a carboxylic acid group (-46 Da). Other fragments at *m/z* 247.2397 [C_16_H_24_O_2_]^+^, *m/z* 219.1735 [C_15_H_24_O]^+,^ and *m/z* 207.1700 [C_14_H_23_O]^+^ representing the internal fragmentation pattern of lupane-type triterpenes. Also, a fragment ion at *m/z* 203.1778 as the base peak produced through decarboxylation of *m/z* 247.2397. An intense peak at *m/z* 189.1612 was attributed to the loss of a water molecule from *m/z* 207.1700 fragment ion. Therefore, peak (71) was tentatively identified as betulinic acid [58, 63].

Thus, promising triterpenoids were identified in the current study, mainly pentacyclic type triterpenoids for the first time in genus *Vitex, viz.*. pomaceic acid (peak 56) and pomolic acid (peak 68) as well as previously identified triterpenoids from genus *Vitex* including maslinic acid (peak 63), euscaphic acid (peak 64), ursolic acid (peak 66) and betulinic acid (peak 71).

### Fatty acid derivatives

Peak (3) [tR at 0.412 min, (M-H)^-^ at *m/z* 387.1140 (C_13_H_23_O_13_)^-^] showed fragment ions at *m/z* 341 [M-2 H-COOH]^-^ corresponding to decarboxylation (-46 Da) of the molecular ion and the other two fragment ions at *m/z* 221 and 179 resulting from the successive natural loss of hexosyl moiety and CH_2_O. Peak (3) was tentatively identified as 7-(*α*-D-hexosyloxy)-2,3,4,5,6-pentahydroxyheptanoic acid [36]. Peak (58) [tR at 3.623, (M-H)^-^ at *m/z* 327.2167 (C_18_H_31_O_5_)^-^] and peak (61) [tR at 3.811, (M-H)^-^ at *m/z* 329.2326 (C_18_H_33_O_5_)^-^] the MS/MS spectrum of peak (58) showed fragment ions at *m/z* 309.1982[M-H-H_2_O]^-^ and *m/z* 291.1942 [M-H-2H_2_O]^-^ indicating the successive loss of two water molecules. Casaletto et al. [79] reported that the intense fragments at *m/z* 229.1437, 211.1337, and 171.1031 were explained by the presence of three hydroxyl group at C9, C12, and C13. Also, the molecular ion of peak 58 exhibited 2Da less than peak 61 indicating the presence of an additional double bond in compound 58. Therefore, peaks 58 and 61 were tentatively identified as 9,12,13-trihydroxyoctadeca-10(*E)*,15(*Z*)-dienoic acid, and 9,12,13-trihydroxyoctadec-10-enoic acid. Peak (65) [tR at 4.654 min, (M-H)^-^ at *m/z* 313.2376 (C_17_H_13_O_6_)^-^] showed the typical fragmentation pathway of hydroxy fatty acids that starts with successive loss of water molecules at *m/z* 295.2271[M-H-H_2_O]^-^ and *m/z* 277.2151[M-H-2H_2_O]^-^. The base peak at *m/z* 201.1126 [M-H-112]^-^ indicated the loss of the aliphatic group and an intense peak at *m/z* 183.1390 represented the loss of water from the base peak ion [60]. Therefore, peak (65) was tentatively identified as dihydroxy-octadecenoic acid I. Peak (69) [tR at 5.182 min, (M-H)^-^ at *m/z* 295.2268 (C_18_H_31_O_3_)^-^] showed an intense base peak at *m/z* 277.2172 [M-H-H_2_O]^-^. The characteristic fragment ions at *m/z* 195.1384, 183.1024, and 171.1019 showed successive loss of aliphatic groups which were originally related to the fragmentation pattern of hydroxy fatty acid residues. The fragmentation pattern of peak (70) [tR at 5.412 min, (M-H)^-^ at *m/z* 293.2116 (C_18_H_29_O_3_)^-^] is comparatively similar to peak (69) with 2Da less indicating the presence of an extra double bond [62]. Therefore, peaks (69) and (70) were tentatively identified as hydroxylinoleic acid and hydroxylinolenic acid, respectively.

Interestingly, this study assigned promising fatty acid derivatives for the first time in genus *Vitex, viz.* 9,12,13-trihydroxyoctadeca-10(*E)*,15(*Z*)-dienoic acid (peak 58), 9,12,13-trihydroxyoctadec-10-enoic acid (peak 61), dihydroxy-octadecenoic acid I (peak 65), hydroxylinoleic acid (peak 66) and hydroxylinolenic acid (peak 70). In addition to previously identified compound from *V. negundo* leaves [36] identified as 7-(*α*-D-glucopyranosyloxy)-2,3,4,5,6- pentahydroxyheptanoic acid (peak 3).

### Determination of total phenolic (TPC) and total flavonoid contents (TFC)

The total phenolic and flavonoid contents of the defatted methanol extract of *V. pubescens* bark were quantified spectrophotometrically as gallic acid and rutin equivalents, respectively, as illustrated in Fig. S2. The total phenolic and flavonoid contents were quantified as 138.61 ± 9.39 µg GAE/mg extract and 119.63 ± 4.62 µg RE/mg extract, respectively. in comparison with the methanol extract of *Vitex agnus castus* fruits, the TPC and TFC were found to be 46.50 ± 1.39 µg GAE/mg extract and 10.80 ± 0.26 µg quercetin equivalent /mg extract, respectively [80]. Moreover, the ethanol extract of *Vitex negundo* and *Vitex trifolia* leaves exhibited TPC of 89.71 mg GAE/g and 77.20 mg GAE/g, respectively, besides, TFC 63.11 mg QE/g and 57.41 mg QE/g [81], . The total defatted methanol extract of *V. pubescens* exhibited comparatively higher values of TPC and TFC among other *Vitex* species. These significant values of the total defatted methanol extract would rely on the nature of the identified phytochemicals using UPLC-ESI-MS/MS which revealed the richness of VT with polyphenolic compounds, viz. phenolic acids and flavonoids, especially, di-*O*-caffeoylquinic acid and flavone-*C*-glycosides, viz. orientin − 2``-*O*-rhamnoside (peak 32), orientin (peak 35) and vitexin (peak 38).

## Evaluation of antioxidant activity

### DPPH free radical scavenging assay

**Fig. 3:**
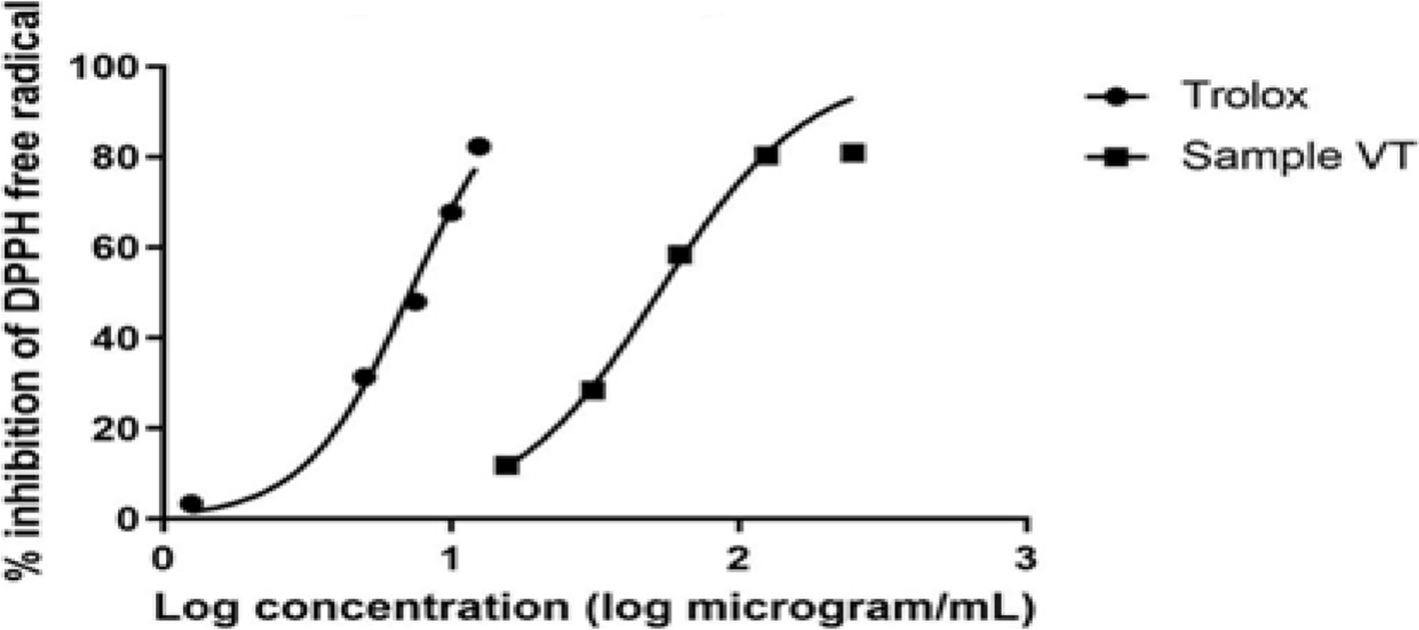
DPPH percent inhibition of *V. pubescens* extract *versus *Trolox standard

Antioxidant activity was assessed using DPPH method on the total defatted methanol extract of *V. pubescens* bark. The VT exhibited promising antioxidant activity with IC_50_ value of 52.79 ± 2.16 µg/mL) compared to standard, Trolox (IC_50_ 7.27 ± 0.309 µg/mL) as illustrated in Fig. 3. In comparison with reported antioxidant activity of genus *Vitex*, the ethanol extract of the leaves of *Vitex negundo* and *Vitex trifolia* exhibited IC_50_ values 40.00 and 70.20 µg/mL, respectively [81]. The promising antioxidant activity of *V. pubescens* methanol extract may induce free radical scavenging, neutralization of the lipid-free radicals, and hindering the decomposition of hydroperoxides into free radicals [81, 82]. Consequently, the detrimental damage induced by oxidative stress would be diminished and potentially delay the progression of Alzheimer’s disease [83]. Herein, from this approach, *V. pubescens* extract might be a prominent effector in the treatment of Alzheimer’s disease.

### Free radical scavenging activity (ABTS) assay

ABTS is one of the commonly applied assays for the determination of radical scavenging activity of the plant extract. It measured the ability of antioxidant compounds to scavenge the ABTS radical cation (ABTS•+), which is generated by the oxidation of ABTS with a strong oxidizing agent [84]. The VT exhibited strong free radical scavenging activity (IC_50_ value of 10.02 ± 1.039 µg/mL) compared to standard, Trolox (IC_50_ 5.721 ± 1.023 µg/mL). In comparison with other reported medicinal plants that exhibited antioxidant and anticholinesterase activities, the ethanolic extract of *Vitex agnus castus* seed exhibited an IC_50_ value 12.66 ± 1.25 µg/mL [85].

There are various therapeutic lines for limiting the progression of Alzheimer’s disease including the conventional line as AchE inhibitory activity strategy and the recent line as antioxidant treatment [86]. Numerous previous studies have linked the use of antioxidant compounds to the reduction of Alzheimer’s disease progression, attributing their efficacy to the prevention of oxidative brain damage as well as their anti-amylogenic action [86, 87]. Based on the aforementioned results, the total defatted methanol extract of *V. pubescens* recorded high contents of total phenolics and flavonoids (138.61 ± 9.39 µg GAE/mg extract and 119.63 ± 4.62 µg RE/mg extract, respectively, which may be related to its promising antioxidant activity assessed using DPPH (IC_50_ 52.79 ± 2.16 µg/mL) and ABTS (IC_50_10.02 ± 1.039 µg/mL).

### Acetylcholinesterase inhibitory activity using Ellman’s microplate assay

**Fig. 4:**
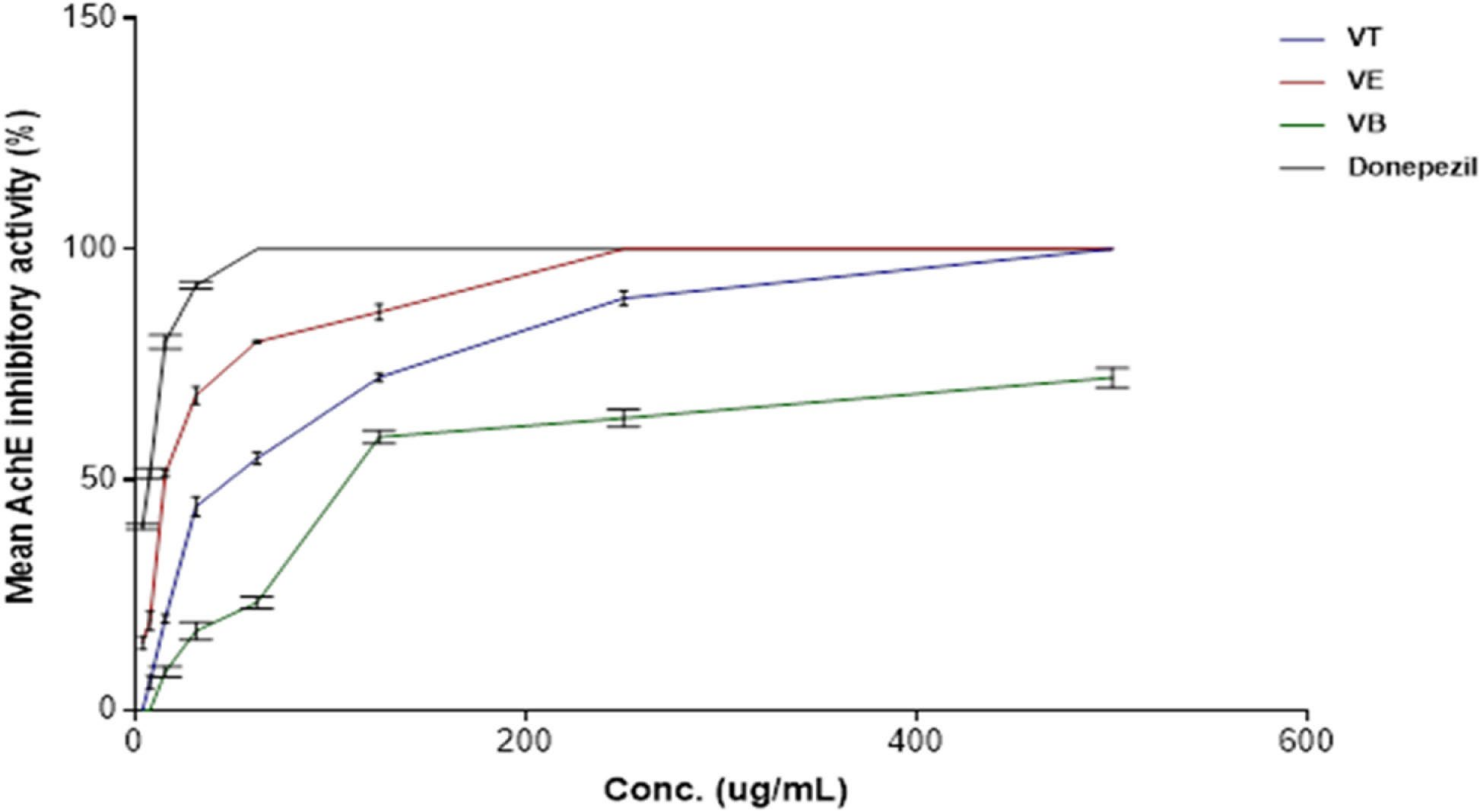
Acetylcholinesterase inhibitory activity of *V. pubescens* total defatted methanol extract and its polar fractions at different concentrations using dose response nonlinear regression test. VT: total defatted methanol extract, VE: polar ethyl acetate fraction and VB: polar *n*-butanol fraction of *V. pubescens *barksversus donepezil control

**Fig. 5:**
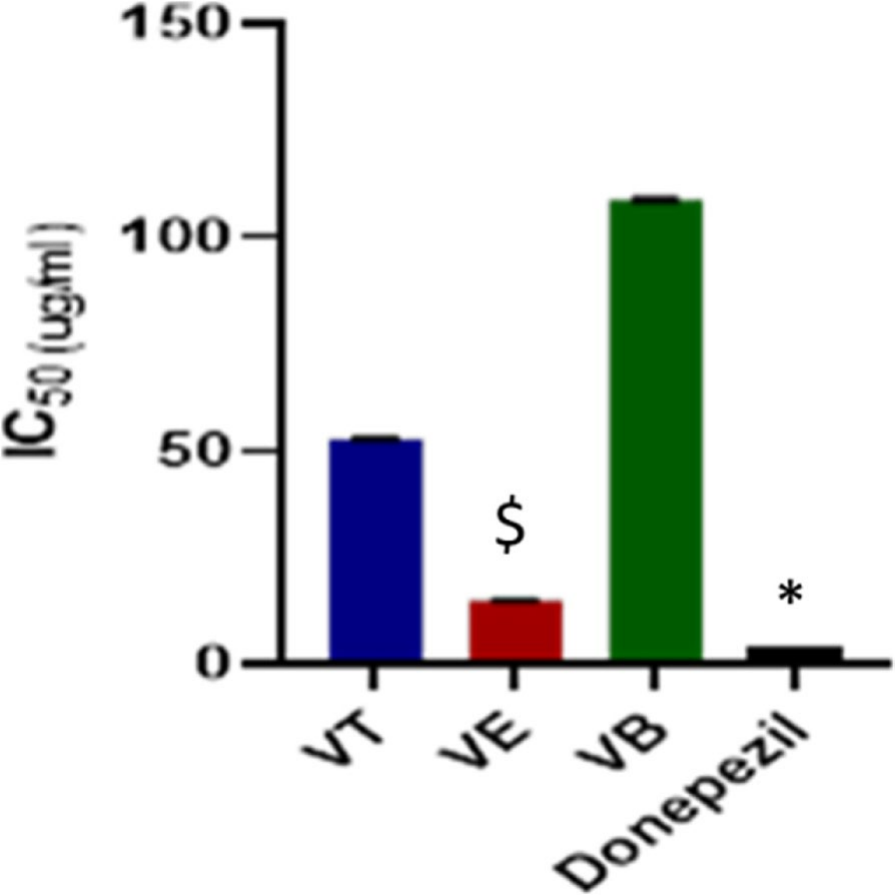
Acetylcholinesterase inhibitory calculated IC_50_ of*V. pubescens* total defatted methanol extract and its polar fractions at different concentrations versus donepezil control using one way ANOVA followed by Tukey’s post hoc test. VT: total defatted methanol extract, VE: polar ethyl acetate fraction and VB: polar *n*-butanol fraction of *V. pubescens *bark.^*,$^: significant different with *P*<0.05

Ach is a vital neurotransmitter for cognitive function and memory [88, 89]. The decline in the level of Ach in the brain cells, attributed to the overactivity of the regulatory AchE enzyme, is considered one of the hallmarks of Alzheimer’s disease pathogenesis [90, 91]. Consequently, the elevation of Ach at the synapses of the brain neurons by inhibiting the activity of AchE would be regarded as one of the agreed therapeutic strategies for the treatment of Alzheimer’s disease [2]. Therefore, screening of the inhibitory activity of the plant extract on AchE level using in-vitro colorimetric Ellman’s assay would aid in the discovery of new natural entities for the treatment of Alzheimer’s disease [92]. The present study assessed the inhibitory activity of the total defatted methanolic extract (VT) of *V. pubescens* bark as well as its polar fractions, viz. the ethyl acetate (VE) and *n*-butanol fractions (VB) for the first time as shown in Table 2; Fig. 4. The calculated IC_50_ of the total defatted methanolic extract (VT) and its polar fractions, VE and VB were represented in Fig. 5 which exhibited IC_50_ values of 52.9, 15.1 and 108.8 µg/Ml, respectively compared to the standard drug, donepezil (IC_50_ = 3.89 µg/mL). The results highlighted the ability of the total defatted extract of *V. pubescens* bark and its polar fractions to exert significant inhibitory activity on AchE. Furthermore, VE (IC_50_ = 15.1 µg/mL) exhibited the strongest inhibitory activity which were statistically different than standard drug, donepezil (*P* value < 0.05). Upon comparing to a previous report on other *Vitex* species, the hydroalcoholic extract of *Vitex negundo* leaves exhibited IC_50_ = 116.00 µg/mL displaying lower AchE inhibitory activity than the defatted methanol extract of *V. pubescens*, whereas it would be in consistence with the inhibitory activity of the *n*-butanol fraction of *V. pubescens* bark [93]. This promising inhibitory activity could be ascribed to the richness of *V. pubescens* defatted methanol extract and its polar fractions with diverse polyphenolic compounds with reported AchE inhibitory activity. These compounds exemplified in phenolic acid derivatives viz. chlorogenic acid (peak 14) [94, 95], *p*-coumaric acid (peak 15) [96], caffeic acid (peak 48) [94, 96], di-*O*-caffeoylquinic acid derivatives (peaks 47 and 51) [95], and flavonoids such as luteolin-*C*-glycosides [97], viz. orientin (peak 35 ) [98] and iso-orientin (peak 62 ) [99] and apigenin-*C*-glycosides, viz. vitexin (peak 38) [100] and isovitexin (peak 52) [101].

**Table 2:** The inhibitory activity of AchE of the total defatted methanolic extract of *V. pubescens* bark and its polar fractions using dose response nonlinear regression test. ^*,$^ IC_50_ significance (*P* value < 0.05)

Concentration (µg/mL)	Mean percentage inhibition of AchE ± SD			
	VT	VE	VB	Donepezil
3.9	0	14.63 ± 1.37	0	39.88 ± 0.58
7.81	6.17 ± 1.4	19.35 ± 2.12	0	51.32 ± 1.2
15.63	19.87 ± 0.92	51.36 ± 0.72	8.32 ± 1.2	79.85 ± 1.5
31.25	44.28 ± 2.1	68.25 ± 1.9	17.24 ± 1.9	92.15 ± 0.72
62.5	54.63 ± 1.3	79.85 ± 0.34	23.38 ± 1.3	100
125	72.15 ± 0.92	86.35 ± 1.7	59.32 ± 1.5	100
250	89.32 ± 1.6	100	63.34 ± 1.8	100
500	100	100	72.08 ± 2.1	100
Calculated IC_50_ (µg/mL) 95% Confidence interval “CI”	52.9 ± 0.40 38.39 to 58.08	15.1^$^±0.20 10.85 to 19.94	108.8 ± 0.52 63.45 to 137.6	3.89^*^±0.11 2.440 to 6.767

Data expressed as mean ±SD, the assay was carried out in triplicate

*VT *total defatted methanol extract, *VE *ethyl acetate fraction, *VB* n-butanol fraction

## Conclusion

In this study, a comprehensive characterization of the metabolic profile of the defatted methanol extract of *V. pubescens* bark as well as its polar fractions, viz. ethyl acetate and *n*-butanol fractions were performed using UPLC-ESI-QTOF-MS/MS for the first time. A total of 71 metabolites were tentatively identified in the defatted methanol extract. Besides, 43 metabolites were annotated in ethyl acetate fraction and 55 metabolites in the *n*-butanol fraction. Polyphenolics including phenolic acids, viz. benzoic acid derivatives and acyl quinic acid derivatives, in addition to flavonoids, viz. luteolin-*C*-glycosides and apigenin-*C*-glycosides were predominant in *V. pubescens* defatted methanol extract, ethyl acetate and *n*-butanol fractions. The metabolic profile shed the light on the potential use of *V. pubescens* bark for the treatment of Alzheimer’s disease as evidenced by the promising in-vitro antioxidant and in-vitro acetylcholinesterase inhibitory activity assessed herein. The current findings provide valuable insights on utilizing *V. pubescens* total defatted methanol extract and its polar fractions as a natural candidate for the treatment of Alzheimer’s disease.

### Supplementary Information

Supplementary Material 1.

## Data Availability

Data are available upon request from the first author, Safa Abdelbaset; safa.abdelbaset@bue.edu.eg.

## Abbreviations

Ach: Acetylcholine
AchE: Acetylcholinesterase sterase
ACTI: Acetylthiocholine iodide
AD: Alzheimer’s disease
VB: *n*-butanol fraction of *V. pubescens*
DCM: Dichloromethane
DPPH: 2,2-Diphenyl-1-picryl-hydrazyl-hydrate
DTNB: 5,5´-Dithiobis [2-Nitrobenzoic Acid])
VE: Ethyl acetate of *V. pubescens*
GAE: Gallic acid equivalent
IC_50_: Half maximal inhibition concentration
NRS: Nitrogen reactive species
ORS: Oxygen reactive species
RE: Rutin equivalent
SD: Standard deviation
TFC: Total flavonoids content
TPC: Total phenolic content
VT: Total defatted methanol extract
UPLC-ESI-QTOF/MS/MS: Ultra-performance liquid chromatography-electrospray ionization-quadrupole time-of-flight MS/MS
