# Supplementary material for: Metabolic profiling of Vitex Pubescens Vahl bark via UPLC-ESI-QTOF/MS/MS analysis and evaluation of its antioxidant and acetylcholinesterase inhibitory activities
Source: BMC Complement Med Ther. 2024 Jun 14;24:232. doi: 10.1186/s12906-024-04520-3 (PMC11177471; doi:10.1186/s12906-024-04520-3)
Supplement: Supplementary file 1 — Supplementary Material 1. [file 12906_2024_4520_MOESM1_ESM.docx]

**SUPPLEMENTARY MATERIAL**

**Metabolic profiling of *Vitex pubescens* Vahl bark *via* UPLC-ESI-QTOF/MS/MS analysis and evaluation of its antioxidant and acetylcholinesterase inhibitory activities.**

Safa Abdelbaset ^1^, Iriny M. Ayoub^2^, Osama G. Mohamed^3,4^, Ashootosh Tripathi^4,5^, Omayma A. Eldahshan^2,6*^,

Dina M. El-kersh^1,7^

^1^ Pharmacognosy Department, Faculty of Pharmacy, The British University in Egypt (BUE), Cairo 11837, Egypt; [safa.abdelbaset@bue.edu.eg](mailto:safa.abdelbaset@bue.edu.eg) orcid.org/0009-0000-0548-7776 and [dina.elkersh@bue.edu.eg](mailto:dina.elkersh@bue.edu.eg) orcid.org/0000-0002-4782-8396.

^2^ Pharmacognosy Department, Faculty of Pharmacy, Ain Shams University, Cairo Governorate 11566, Egypt; irinyayoub@pharma.asu.edu.eg orcid.org/0000-0003-2382-8241 and [oeldahshan@pharma.asu.edu.eg](mailto:oeldahshan@pharma.asu.edu.eg) orcid.org/0000-0002-0972-0560.

^3^ Pharmacognosy Department, Faculty of Pharmacy, Cairo University, Kasr el Aini St., Cairo 11562, Egypt.

^4^ Natural Products Discovery Core, Life Sciences Institute, University of Michigan, Ann Arbor, MI 48109, USA.

^5^ Department of Medicinal Chemistry, College of Pharmacy, University of Michigan, Ann Arbor, MI 48109, USA.

^6^ Center for Drug Discovery Research and Development, Ain Shams University, Egypt

^7^ Drug Research and Development Group (DRD-G), The British University in Egypt (BUE), Cairo 11837, Egypt.

#### ^*^Correspondence:

#### Omayma A. Eldahshan: e-mail address: oeldahshan@pharma.asu.edu.eg

| ESI-MS/MS spectrum of peak (1) *via* positive ionization mode showing fumaric acid. |
| --- |
| 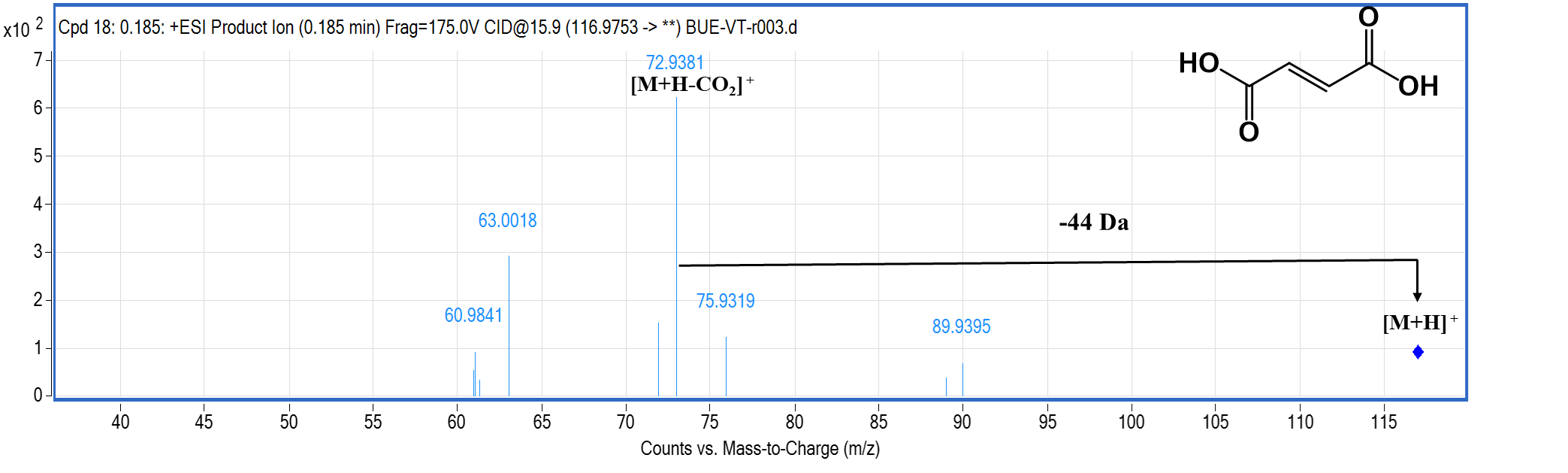 |
| ESI-MS/MS spectrum of peak (2) *via* negative ionization mode showing tartaric acid. |
| 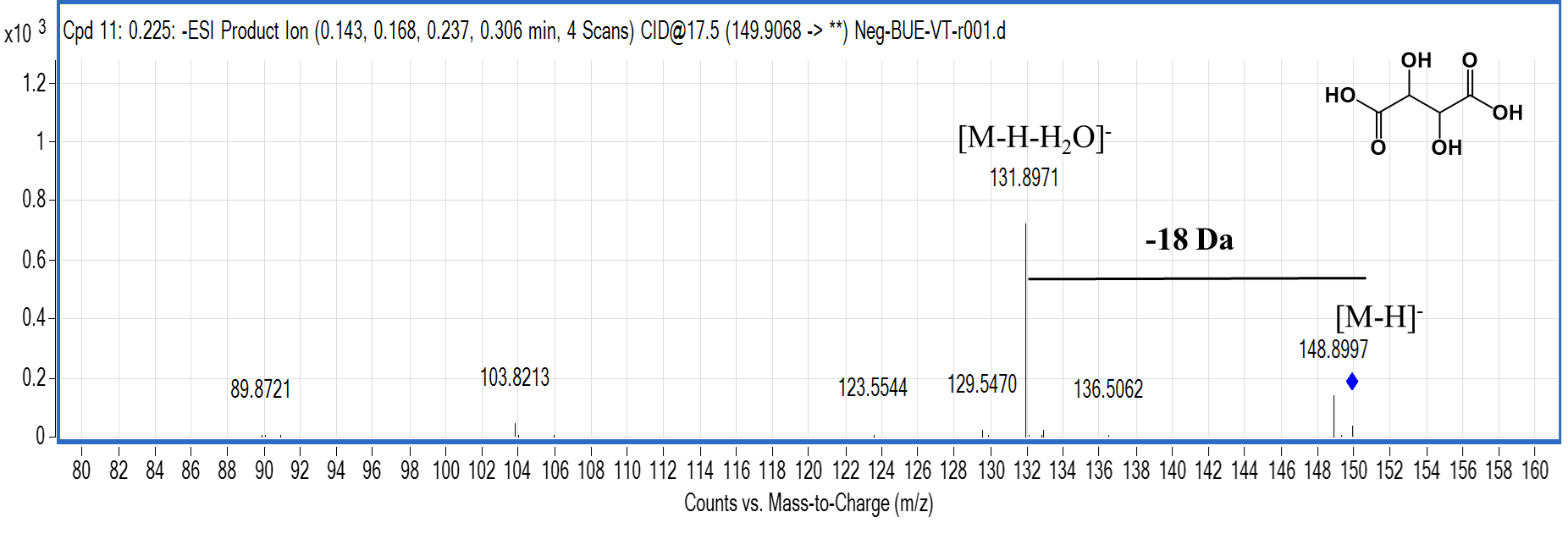 |
| 1. ESI-MS/MS spectrum of peak (3) *via* negative ionization mode showing 7-(α-D-hexosyloxy)-2,3,4,5,6- pentahydroxyheptanoic acid. |
| 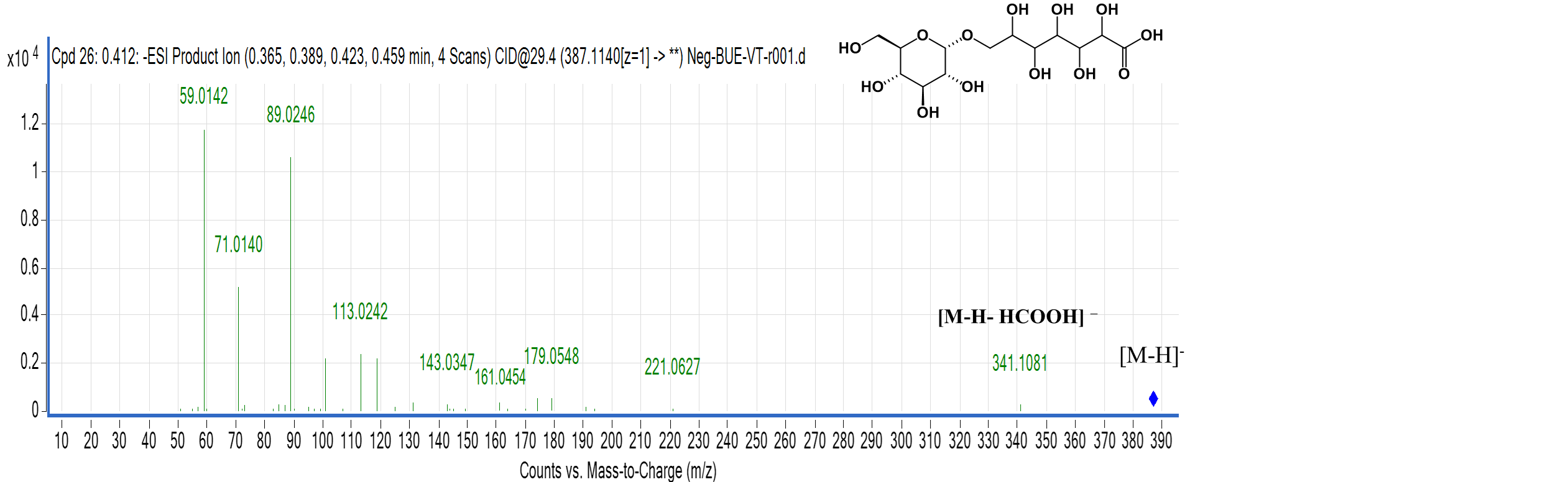 |
| ESI-MS/MS spectrum of peak (4) *via* negative ionization mode showing quinic acid derivative. |
| 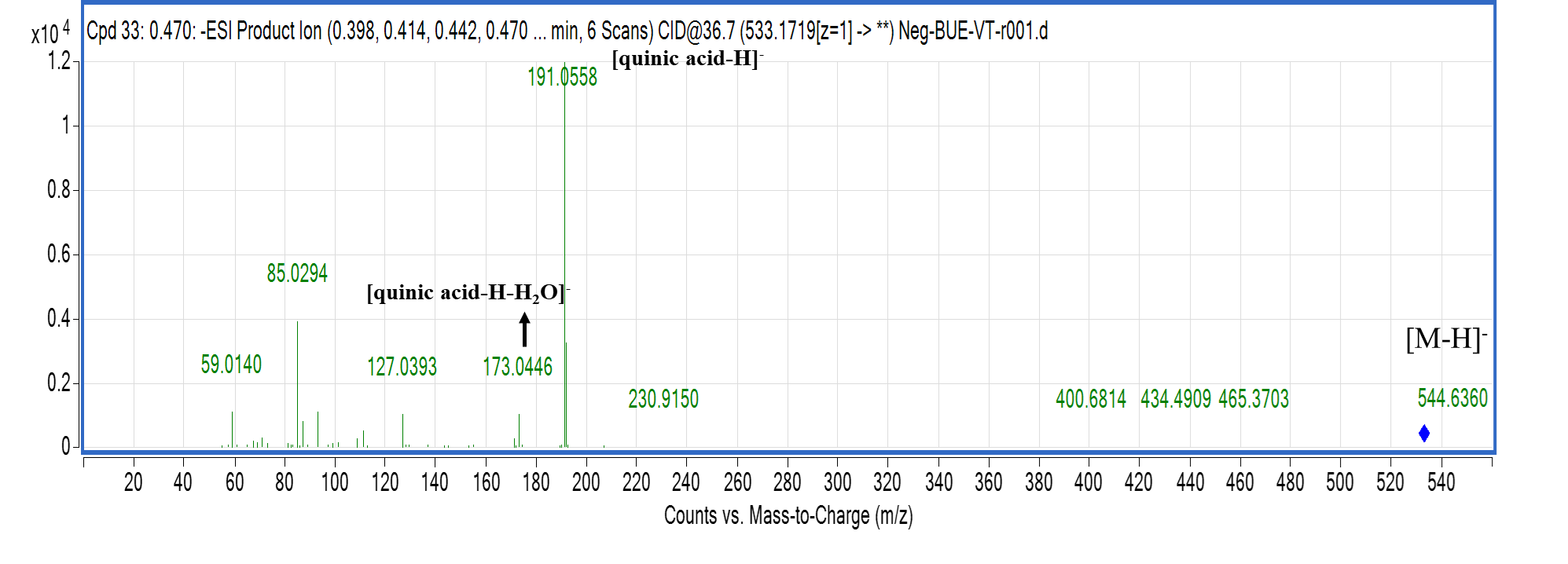 |
| ESI-MS/MS spectrum of peak (5) *via* negative ionization mode showing gallic acid. |
| 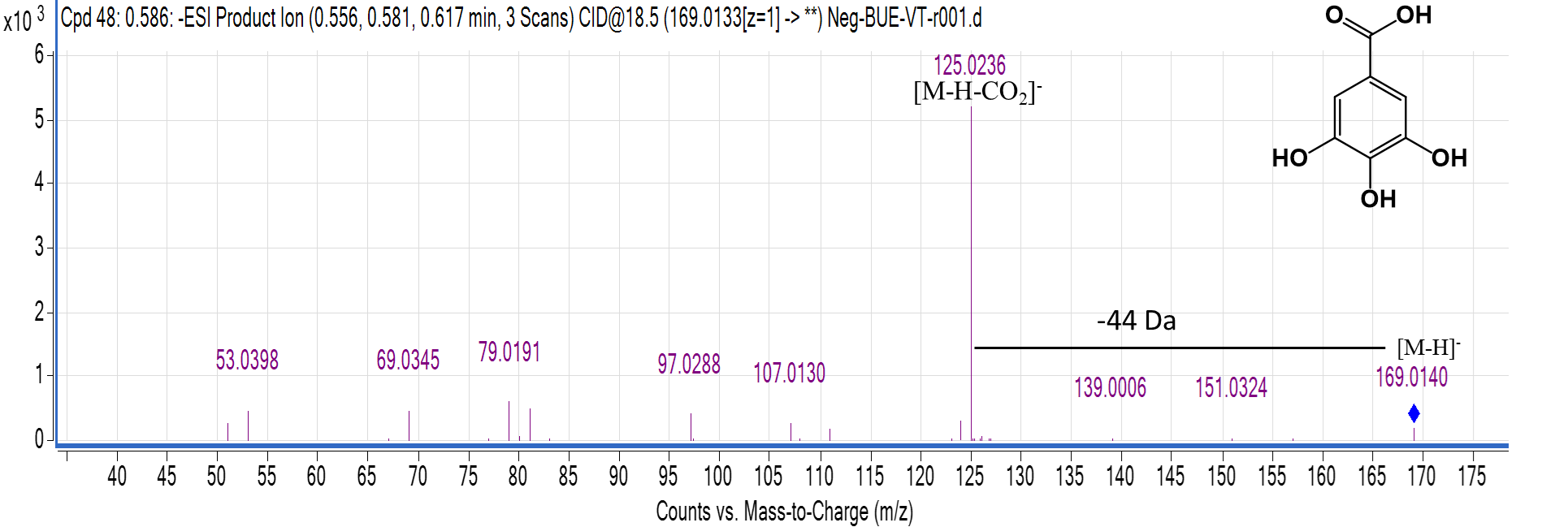 |
| ESI-MS/MS spectrum of peak (6) *via* negative ionization mode showing pyrogallol. |
| 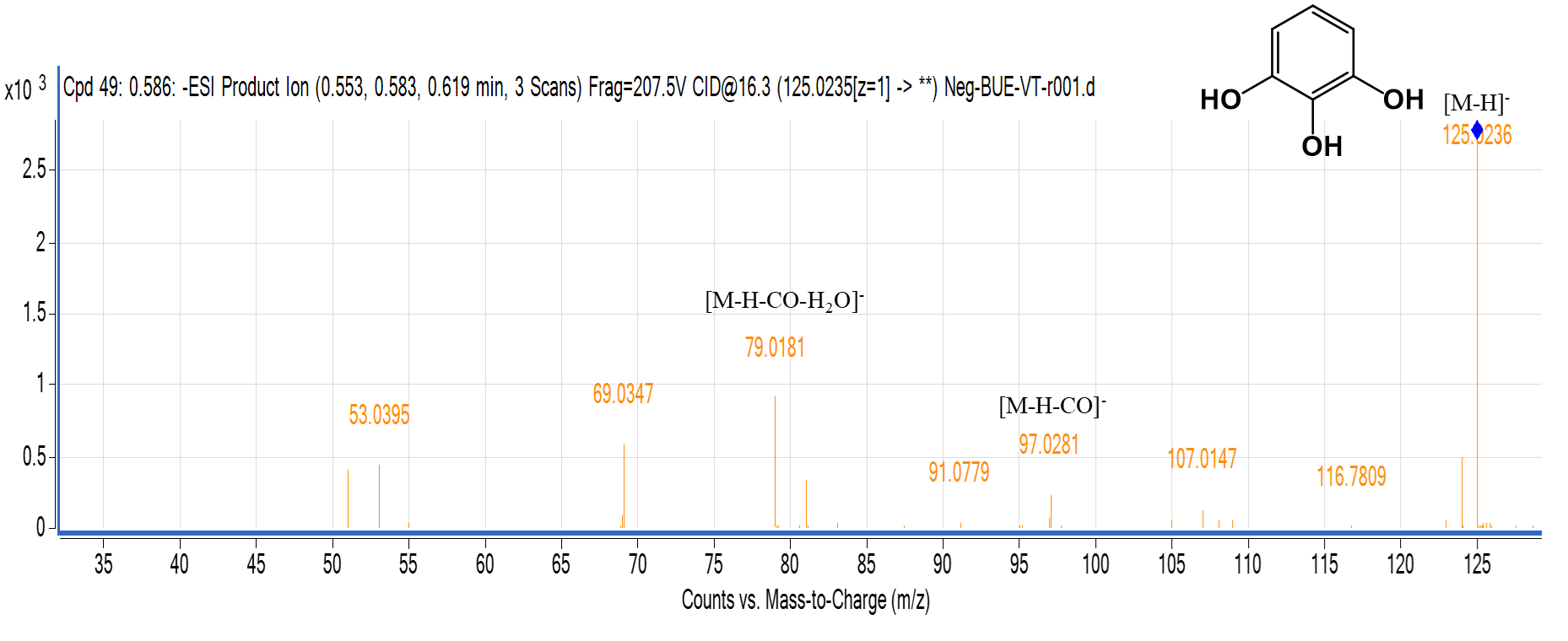 |
| ESI-MS/MS spectrum of peak (7) *via* negative ionization mode showing *O*-*p*-hydroxybenzoyl quinic acid. |
| 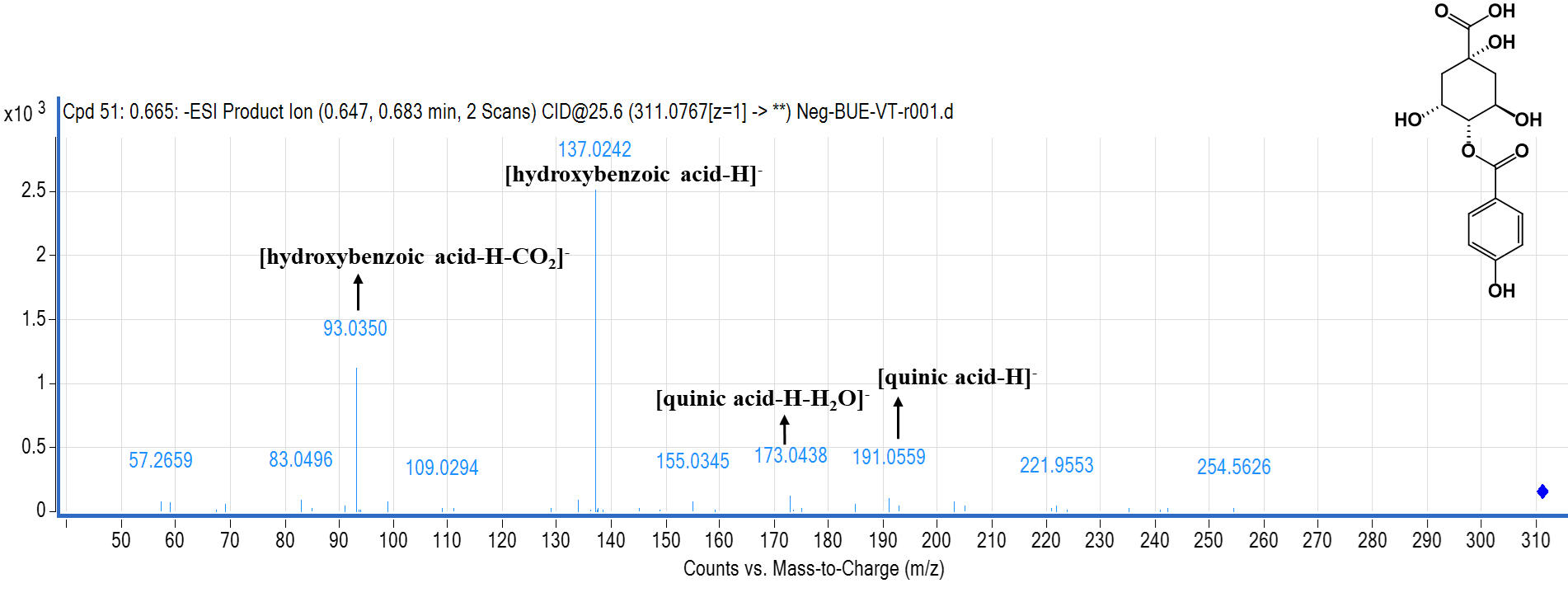 |
| ESI-MS/MS spectrum of peak (8) *via* negative ionization mode showing *p*-hydroxybenzoic acid-*O*- hexoside. |
| 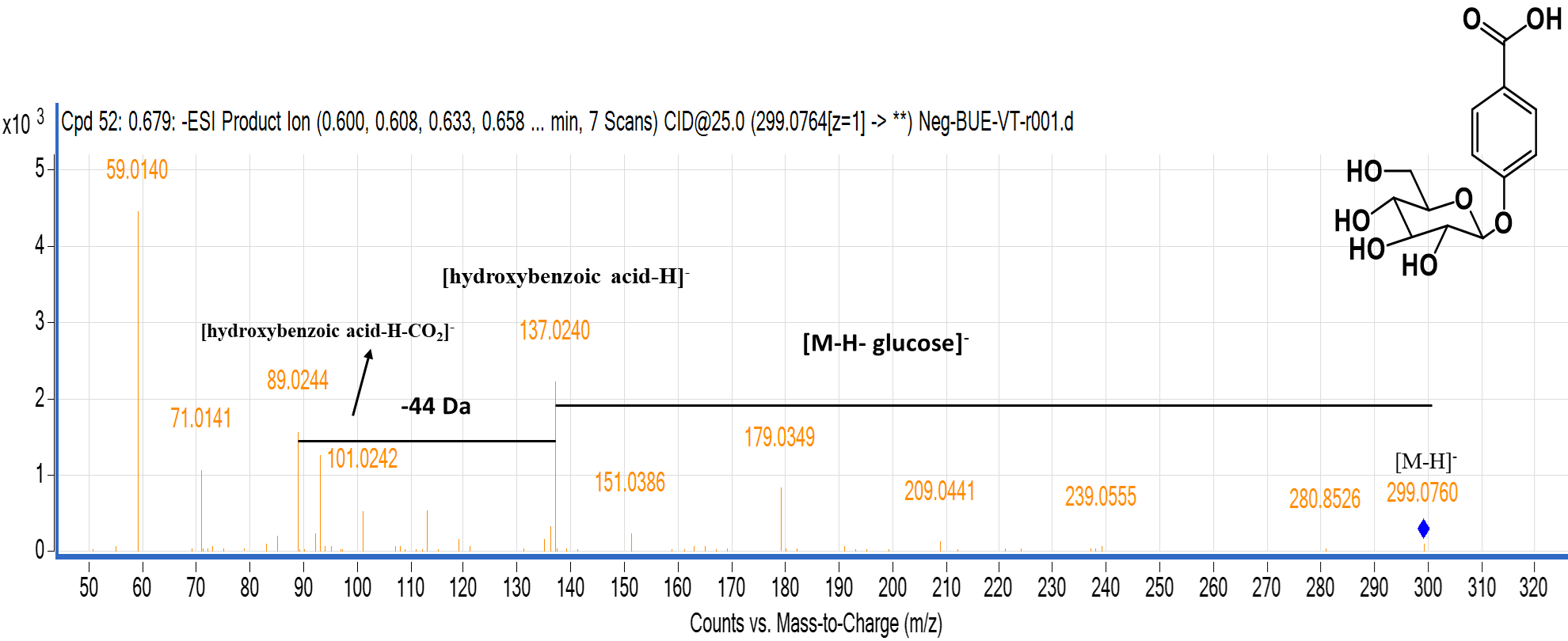 |
| ESI-MS/MS spectrum of peak (9) *via* negative and ionization mode showing hydroxy-methoxy-benzoic acid. |
| 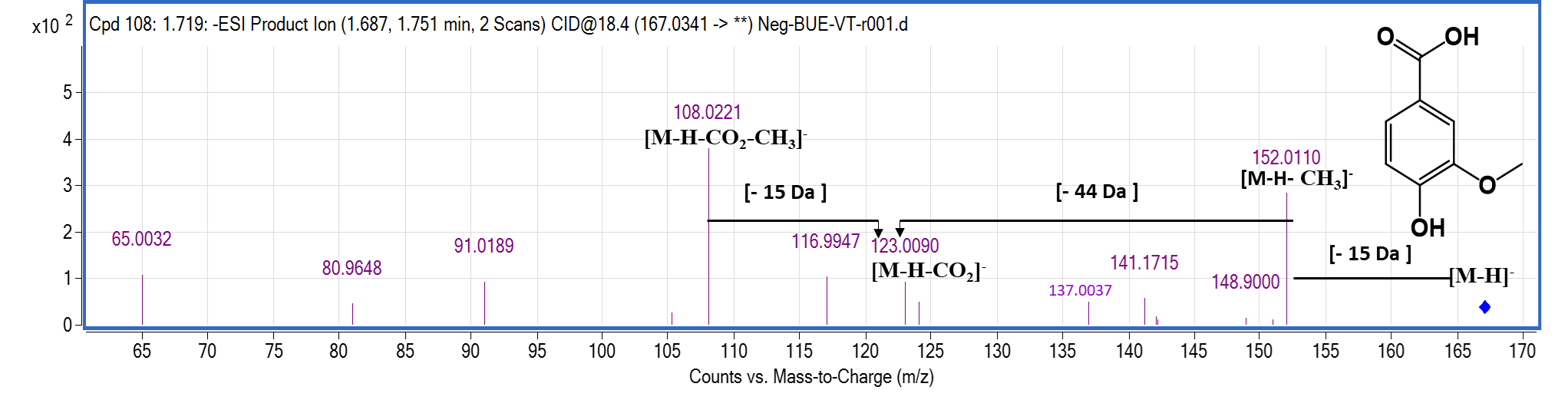 |
| ESI-MS/MS spectrum of peak (10) *via* negative ionization mode showing protocatechuic acid. |
| 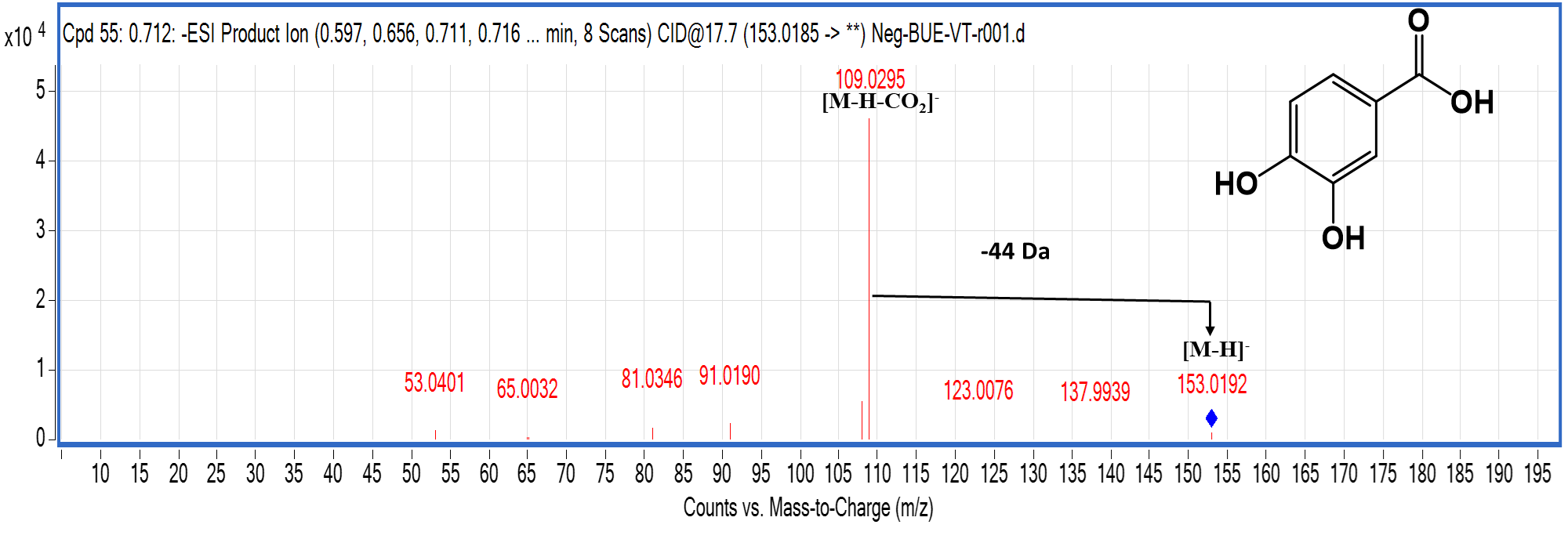 |
| ESI-MS/MS spectrum of peak (11) *via* negative ionization mode showing pyrocatechol. |
| 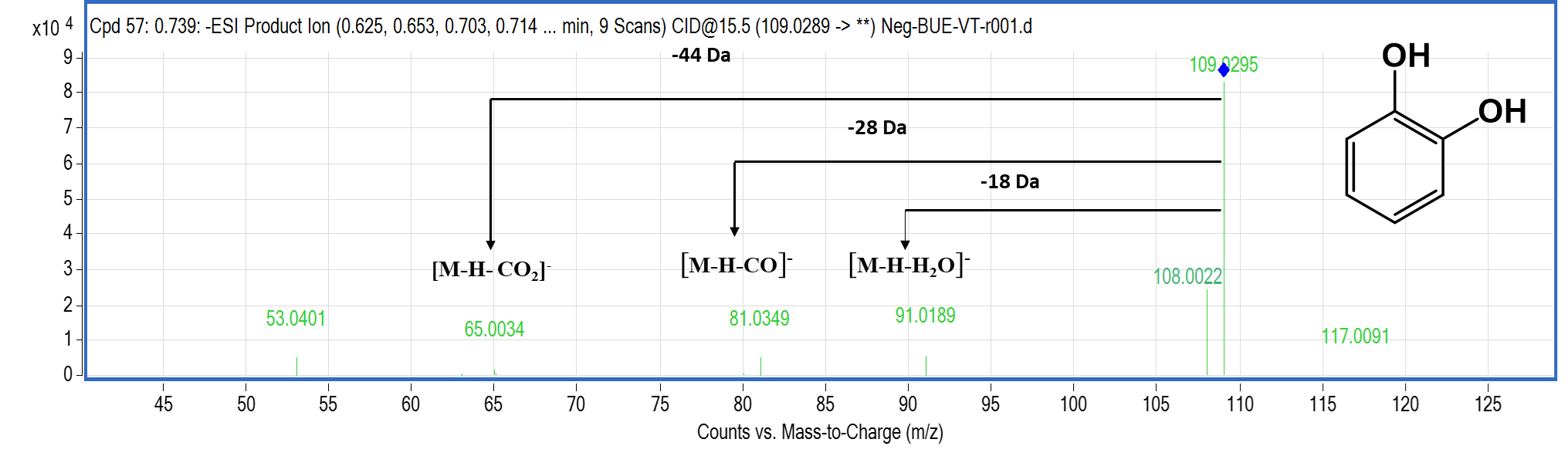 |
| ESI-MS/MS spectrum of peak (12) *via* negative ionization mode showing quinic acid. |
| 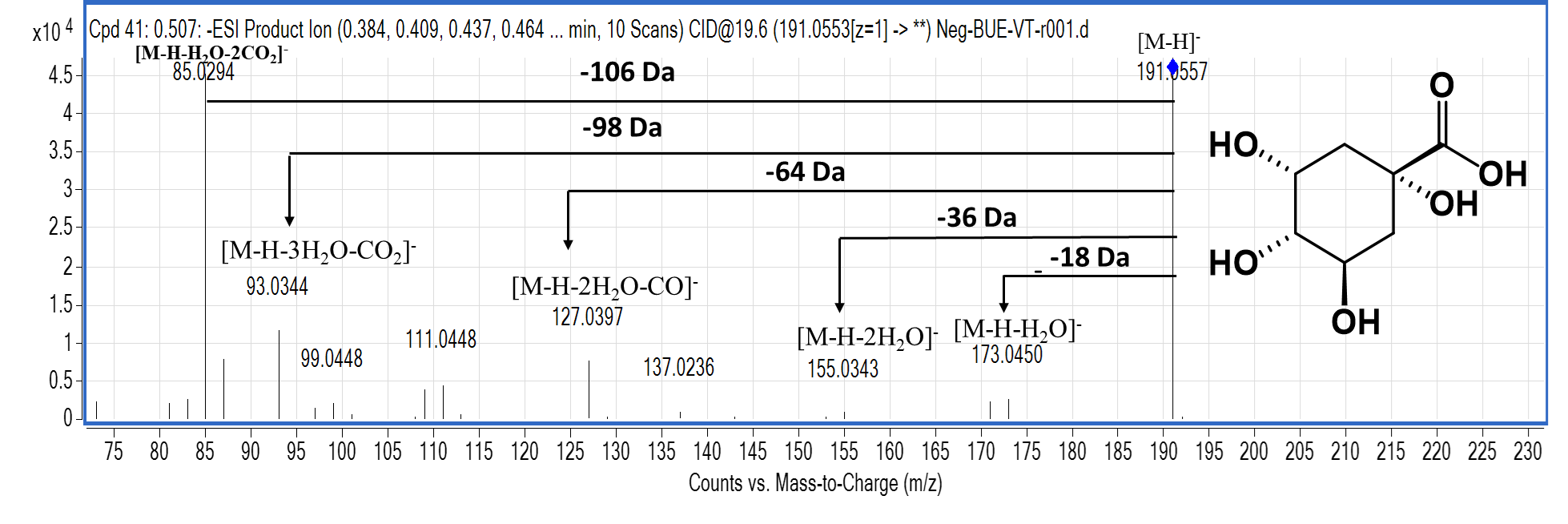 |
| ESI-MS/MS spectrum of peak (13) *via* negative ionization mode showing vanillic acid-*O*- hexoside. |
| 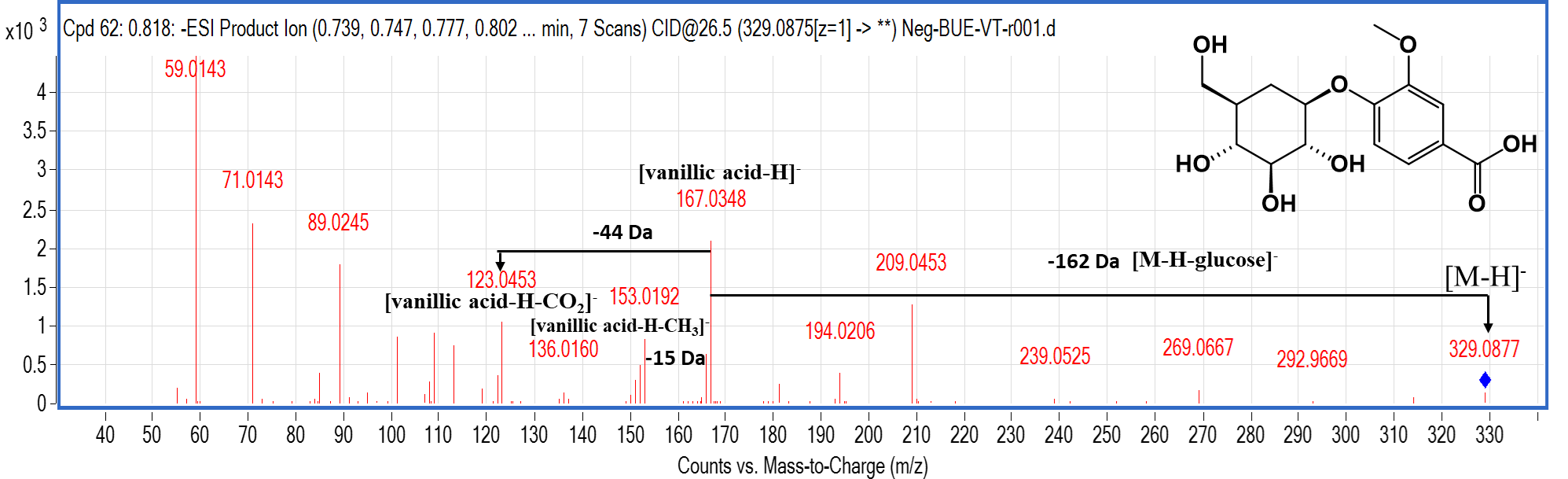 |
| ESI-MS/MS spectrum of peak (14) *via* negative ionization mode showing *O*-Caffeoylquinic acid. |
| 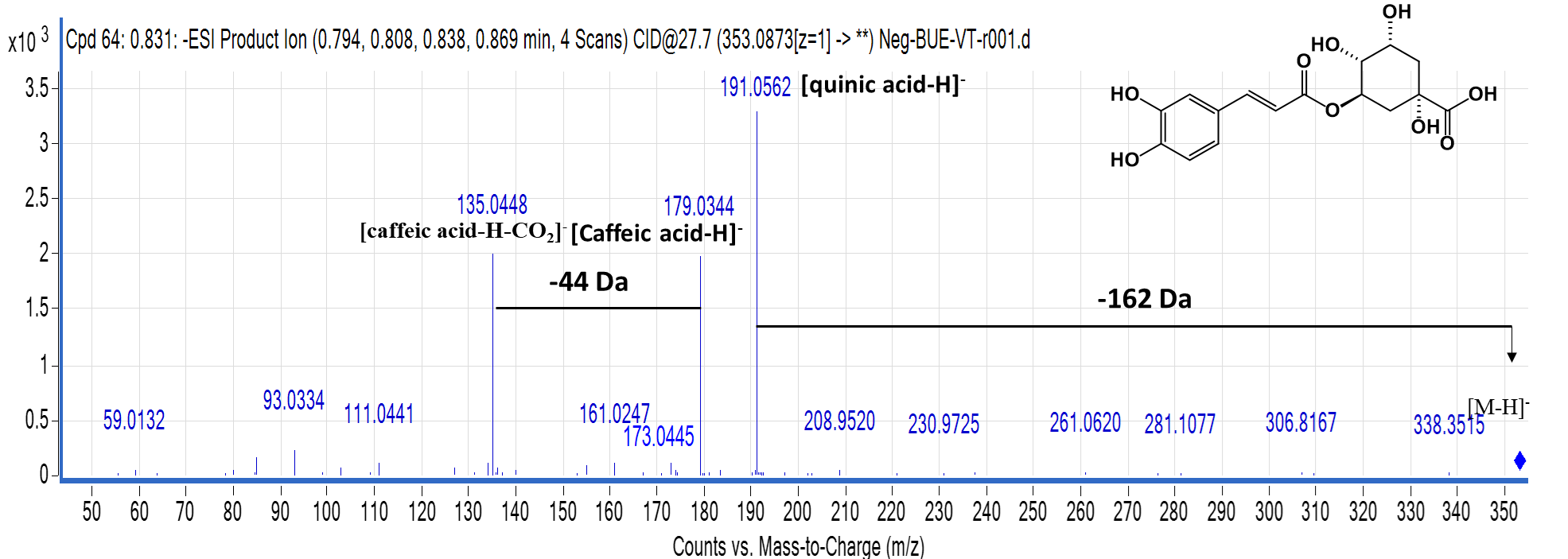 |
| ESI-MS/MS spectrum of peak (15) *via* negative ionization mode showing *p*-Coumaric acid. |
| 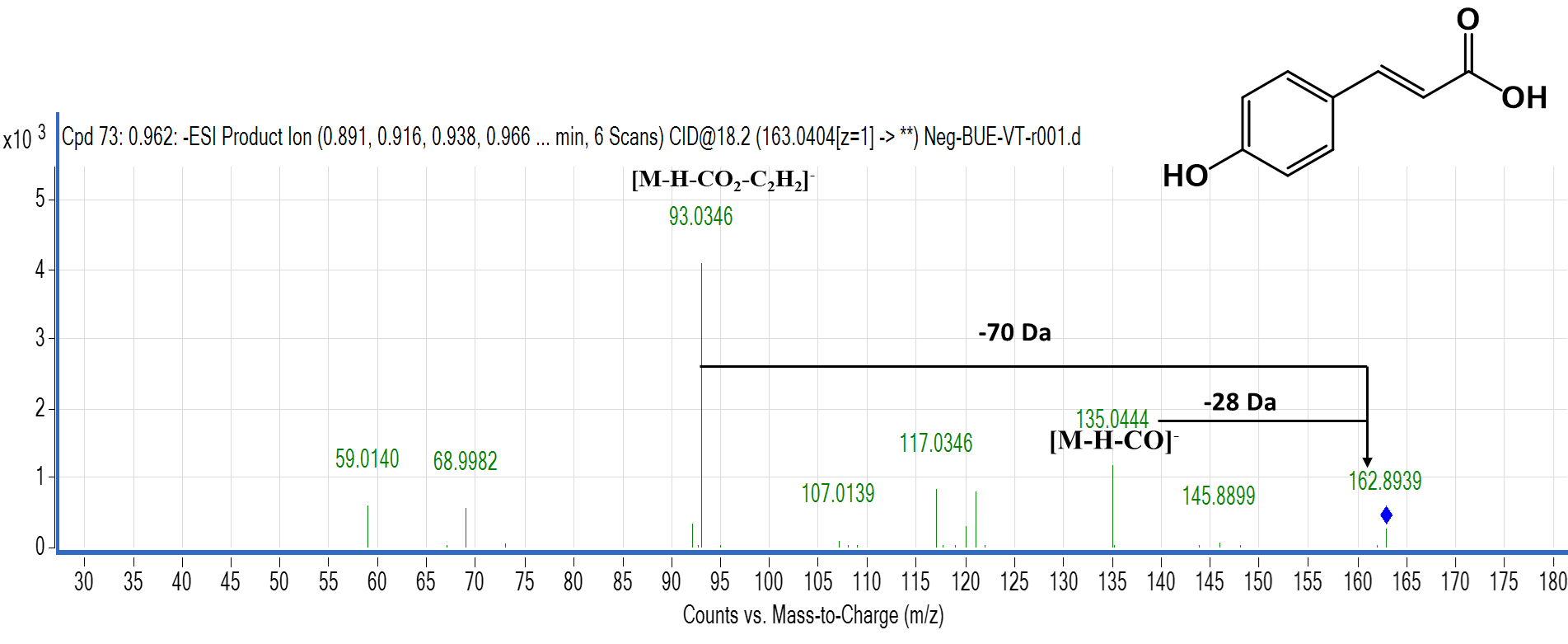 |
| ESI-MS/MS spectrum of peak (16) *via* negative ionization mode showing *O*-vanilloylquinic acid. |
| 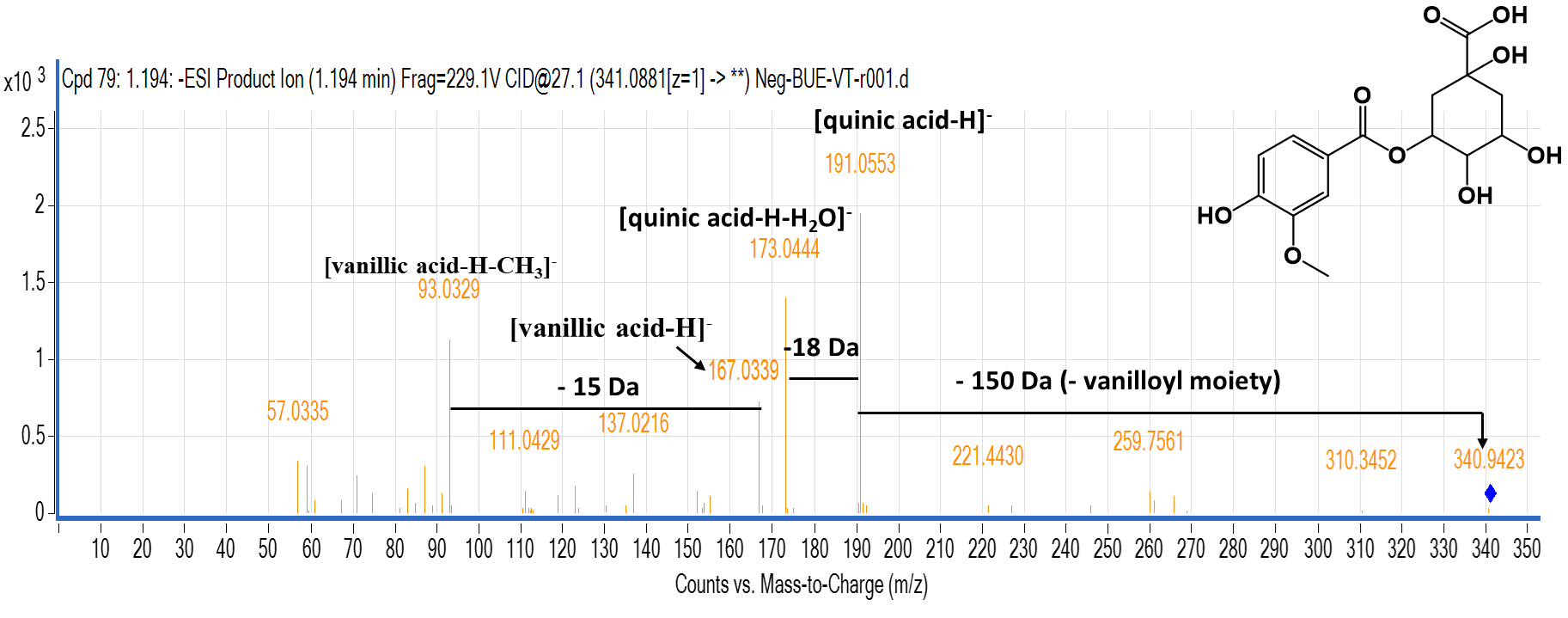 |
| ESI-MS/MS spectrum of peak (17) *via* negative and positive ionization modes showing methyl gallate. |
| 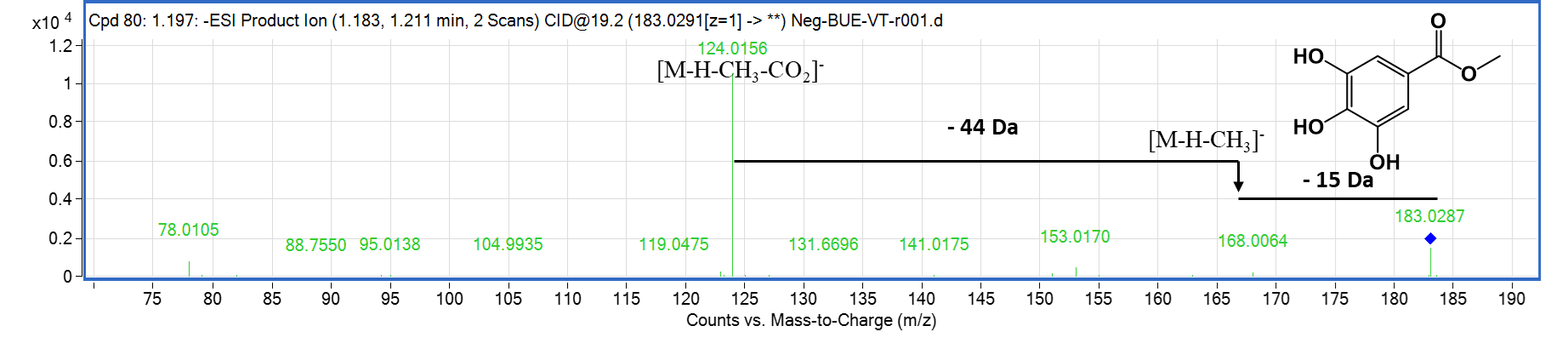 |
| ESI-MS/MS spectrum of peak (18) *via* negative ionization mode showing protocatechualdehyde. |
| 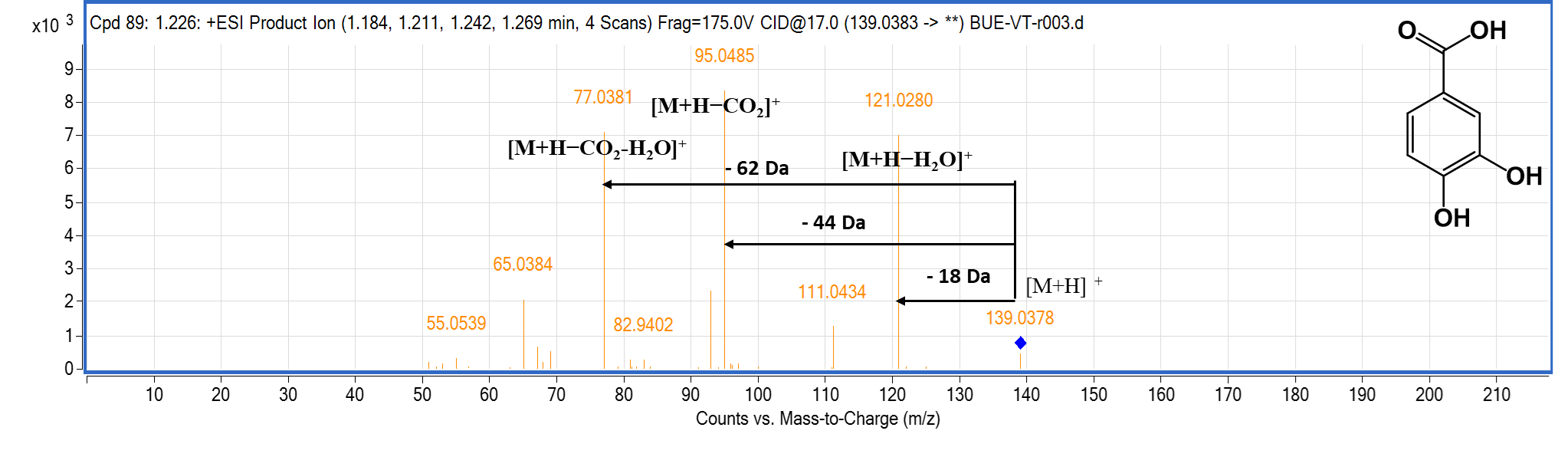 |
| ESI-MS/MS spectrum of peak (19) *via* negative ionization mode showing hydroxybenzoic acid derivatives. |
| 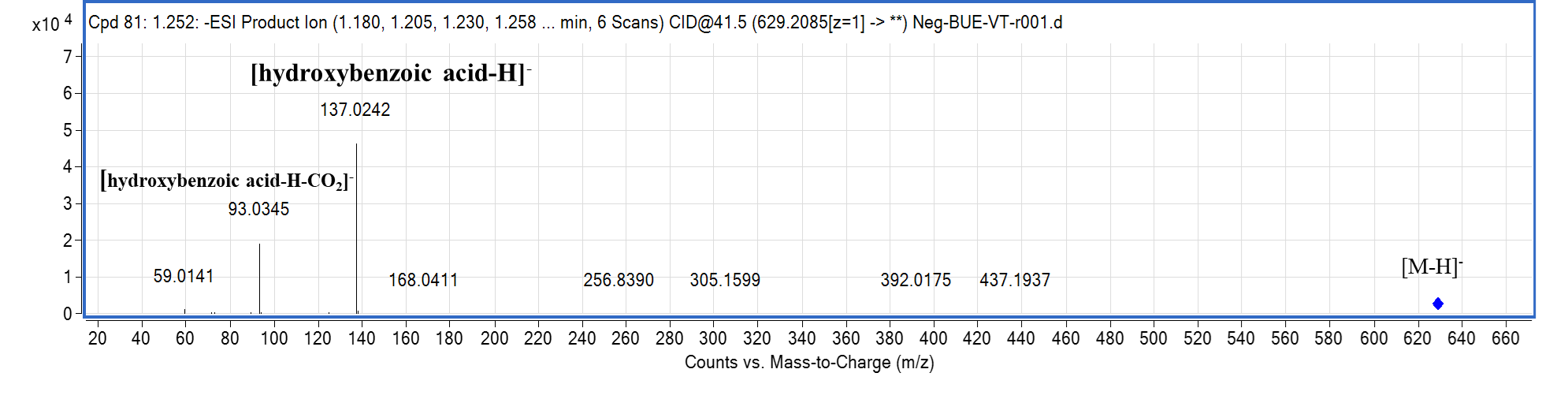 |
| ESI-MS/MS spectrum of peak (20) *via* negative ionization mode showing *p*-hydroxybenzoic acid. |
| 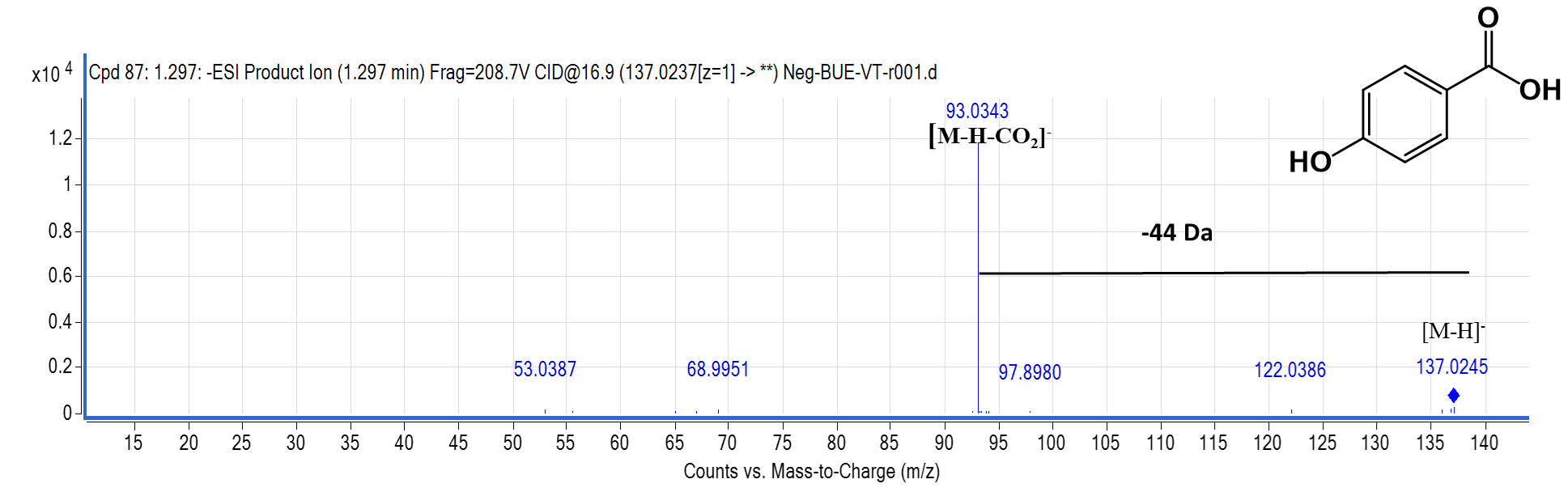 |
| ESI-MS/MS spectrum of peak (21) *via* positive ionization mode showing umbelliferone. |
| 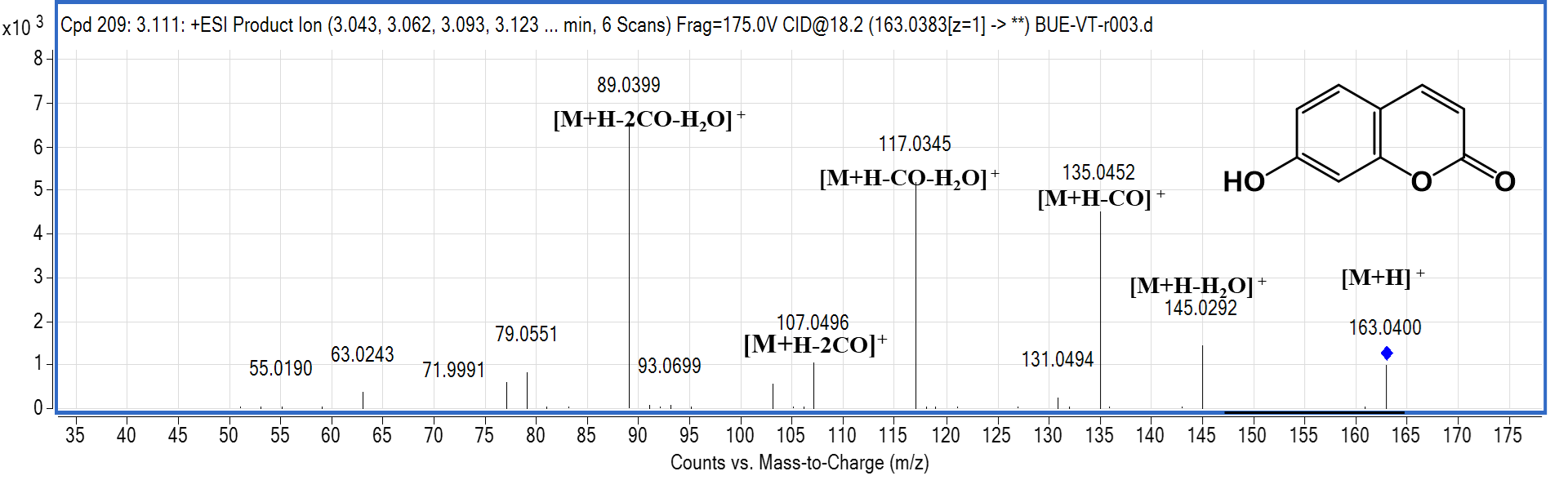 |
| ESI-MS/MS spectrum of peak (22) *via* negative ionization mode showing *O*-Caffeoylquinic acid. |
| 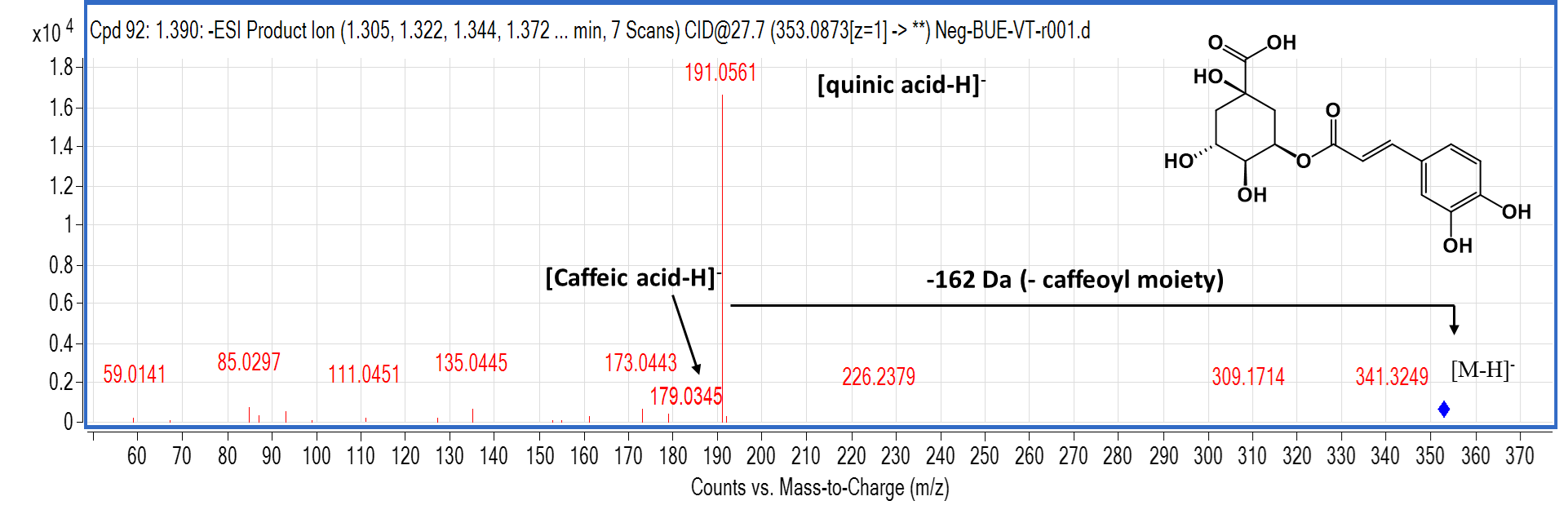 |
| ESI-MS/MS spectrum of peak (23) *via* negative ionization mode showing methyl hydroxybenzoic acid. |
| 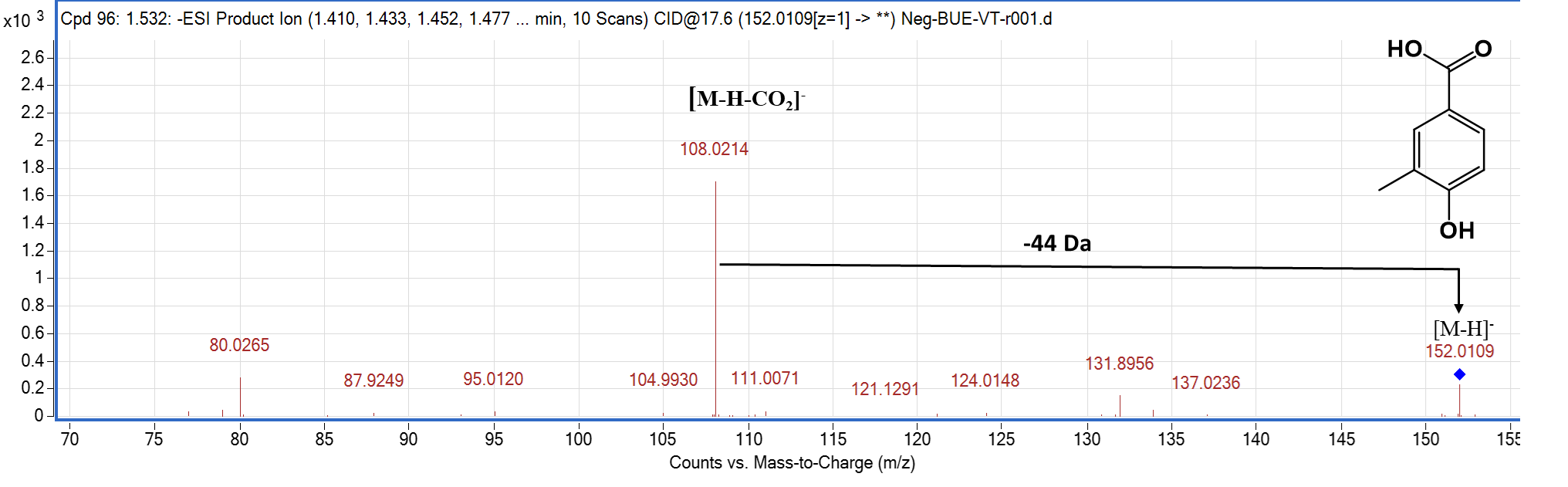 |
| ESI-MS/MS spectrum of peak (24) *via* negative ionization mode showing vanillic acid. |
| 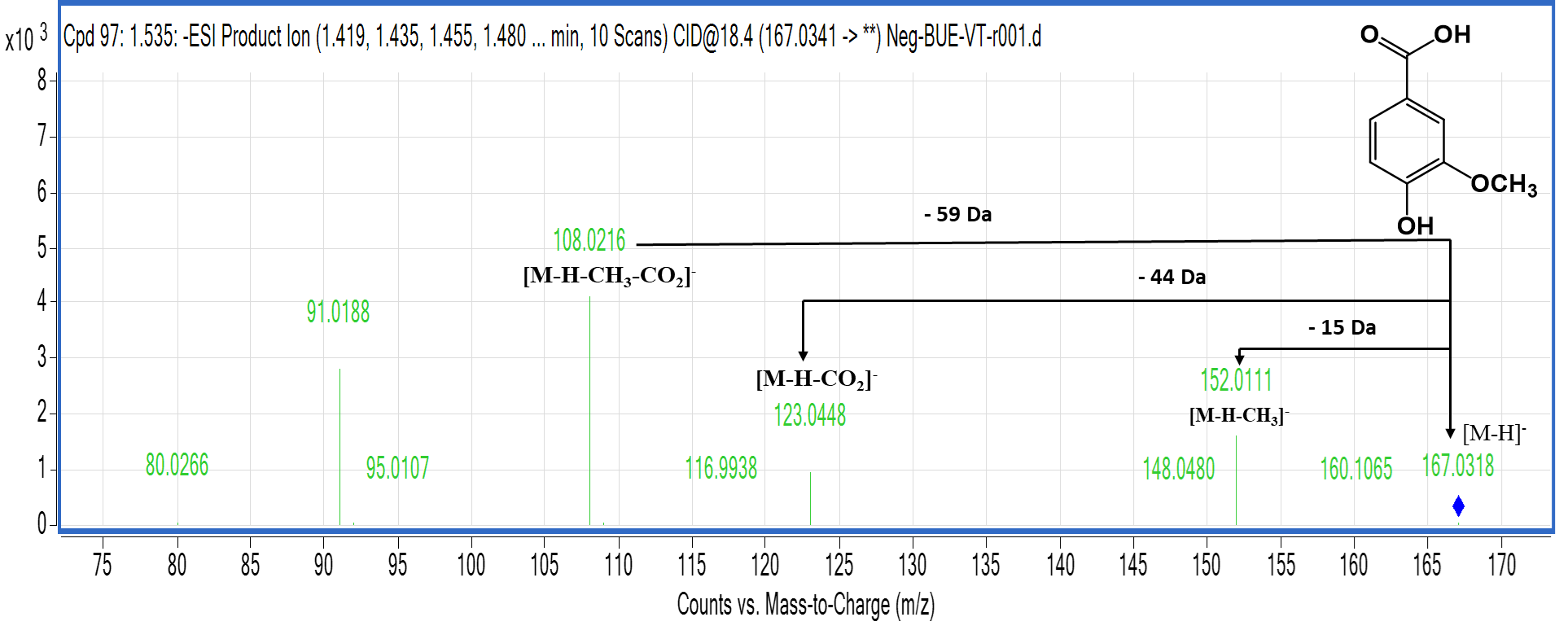 |
| ESI-MS/MS spectrum of peak (25) *via* negative ionization mode showing luteolin-di-*C*-hexoside. |
| 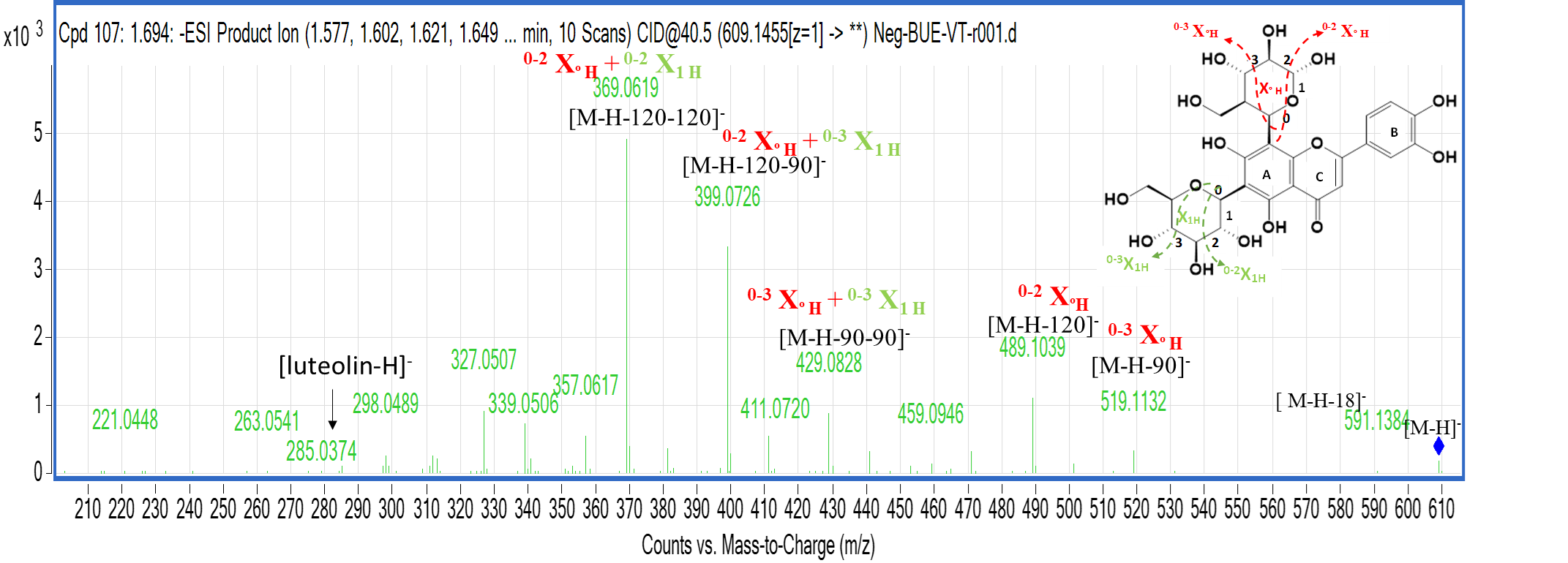 |
| ESI-MS/MS spectrum of peak (26) *via* negative ionization mode showing syringic acid. |
| 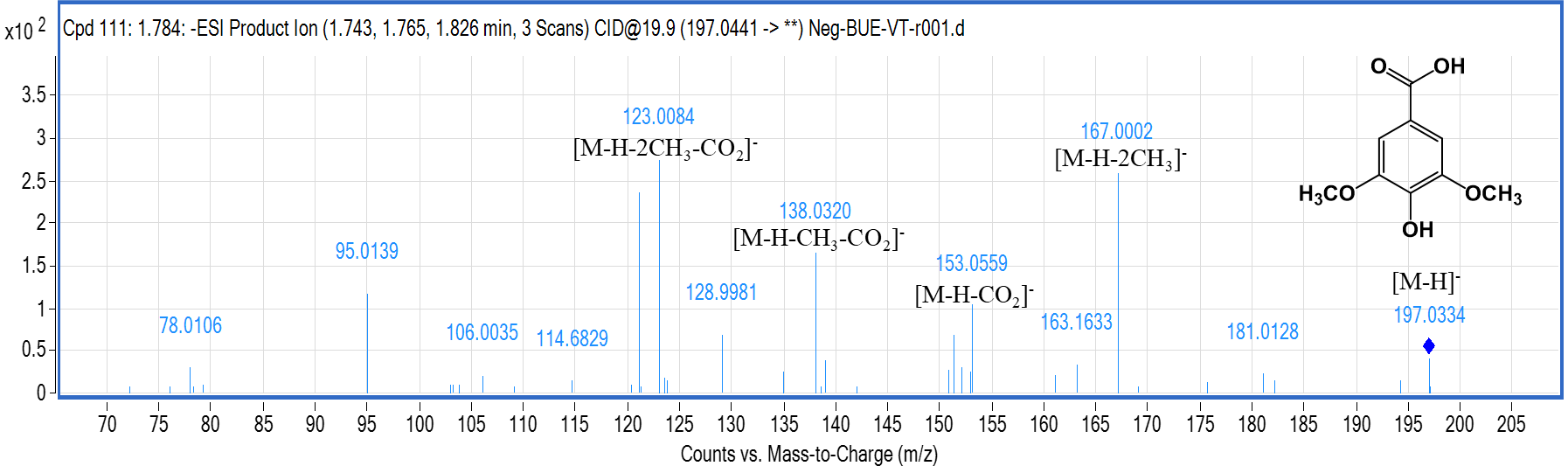 |
| ESI-MS/MS spectrum of peak (27) *via* negative ionization mode showing apigenin-di-*C*-hexoside. |
| 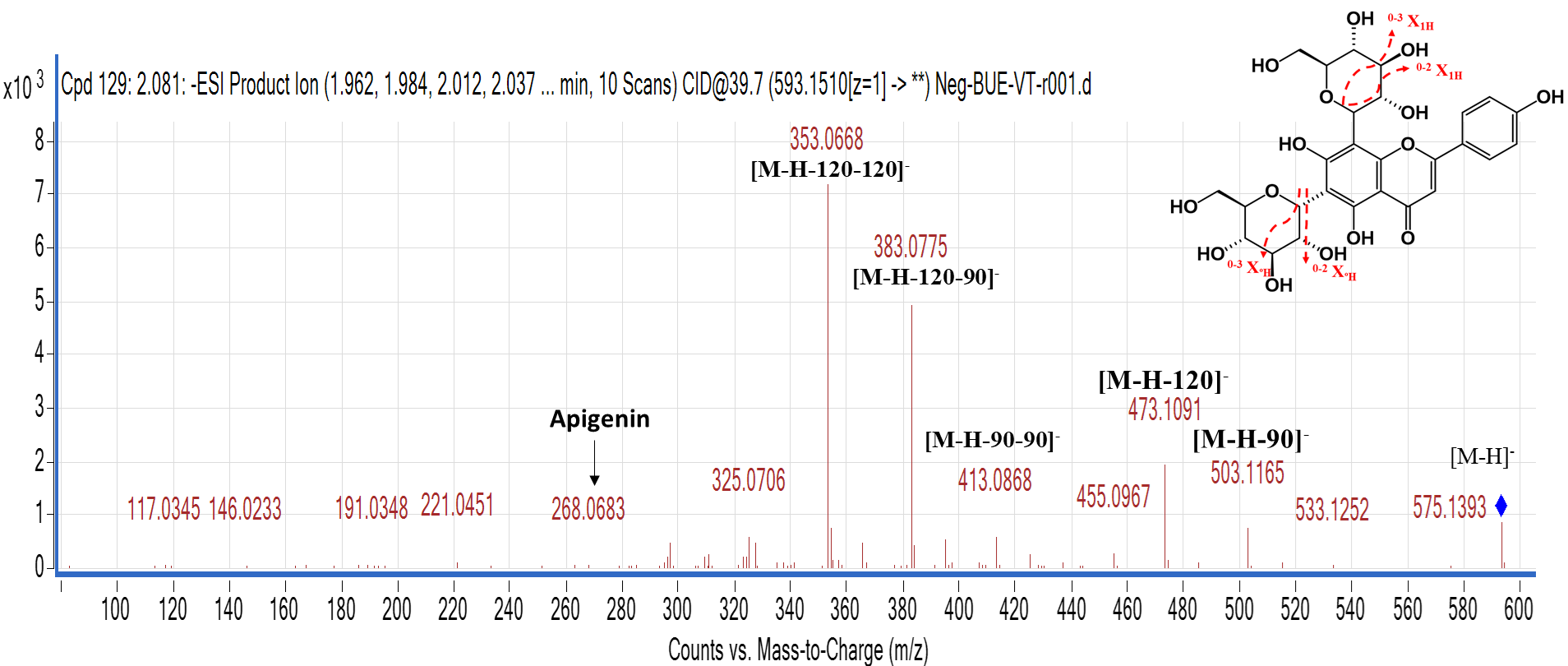 |
| ESI-MS/MS spectrum of peak (28) *via* negative ionization mode showing luteolin-*C*-hexoside-*O*-hexoside-*O*-deoxyhexoside. |
| 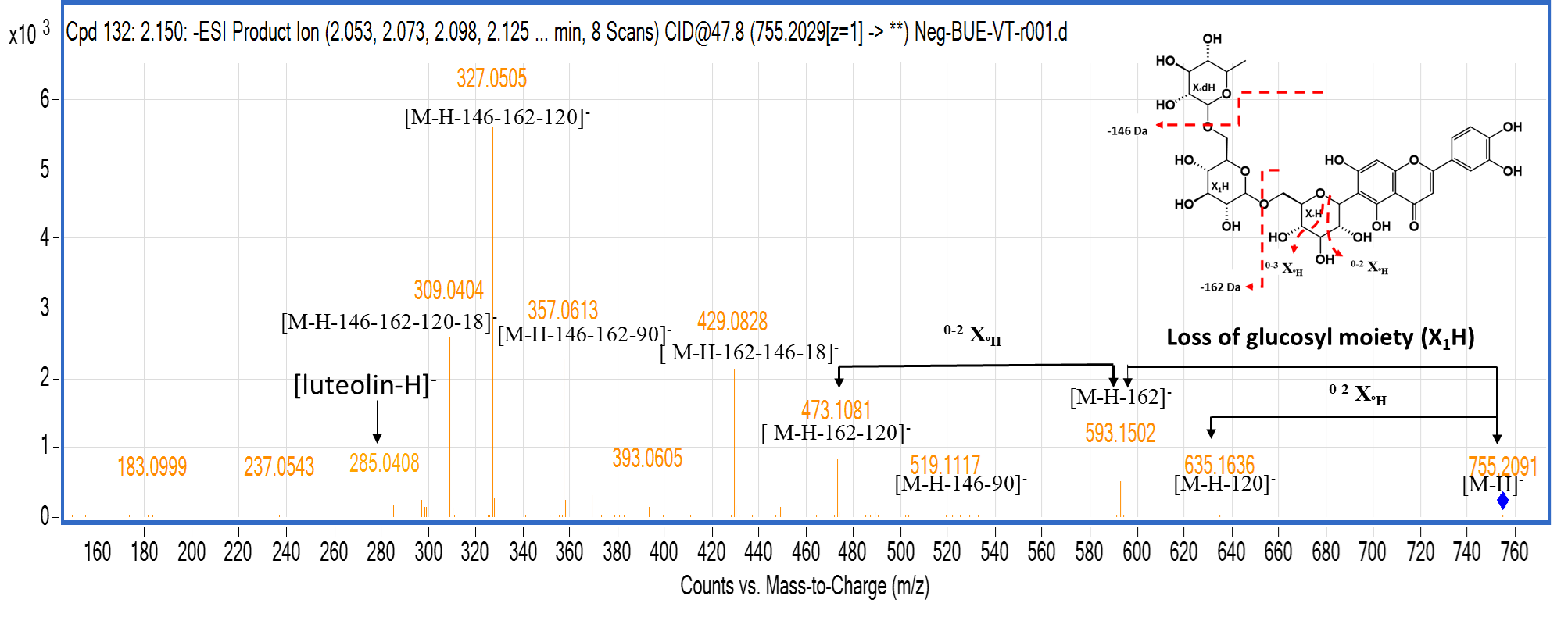 |
| ESI-MS/MS spectrum of peak (29) *via* negative ionization mode showing apigenin-*C*-hexoside-*C*-pentoside. |
| 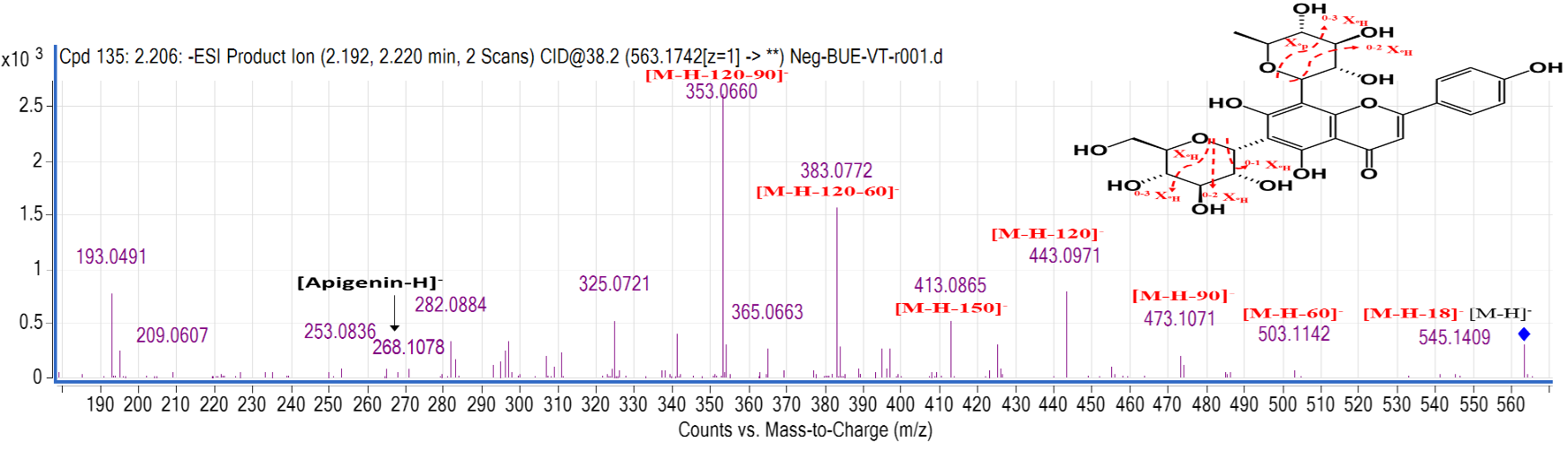 |
| ESI-MS/MS spectrum of peak (30) *via* negative ionization mode showing luteolin-*C*-hexoside-*O*-hexoside. |
| 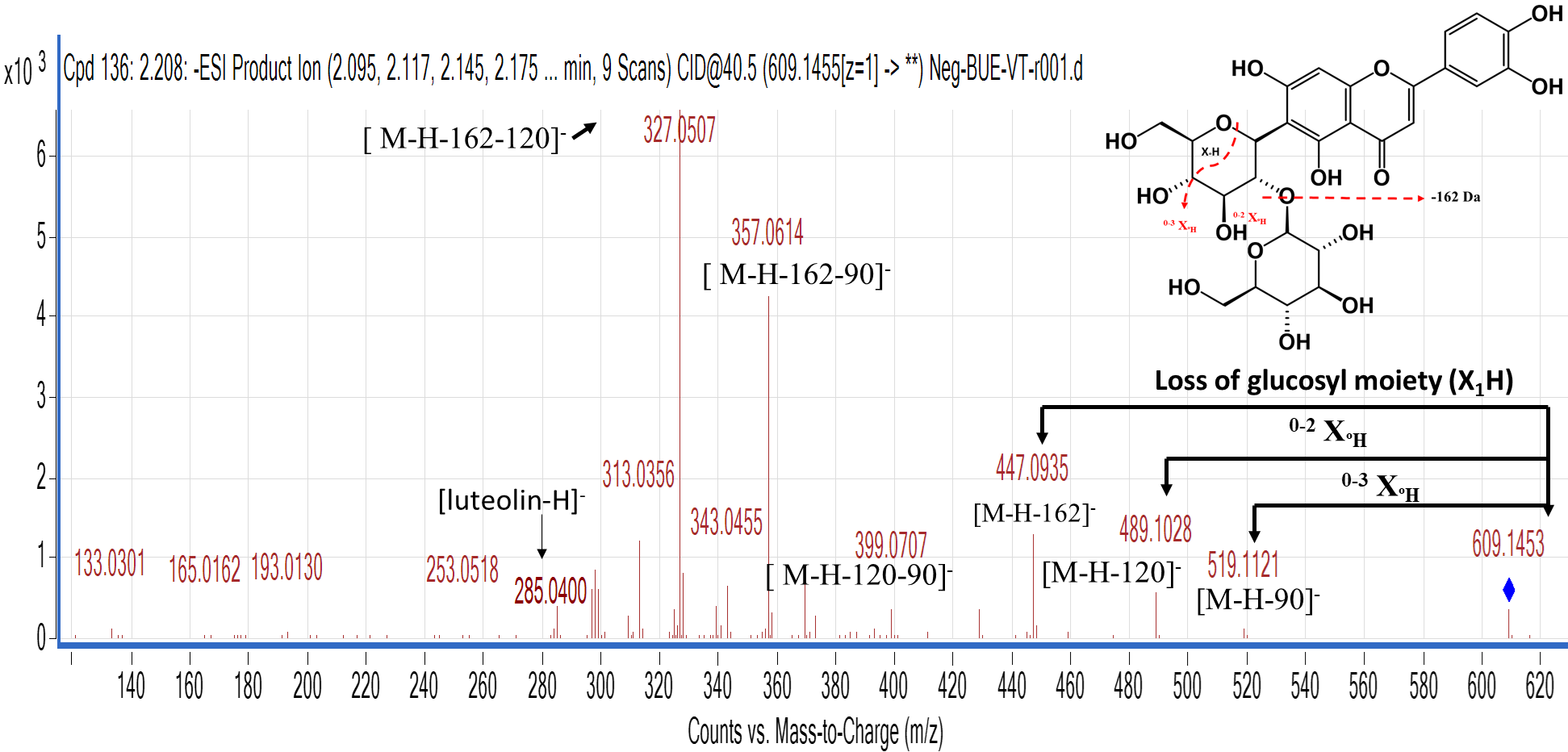 |
| ESI-MS/MS spectrum of peak (31) *via* negative ionization mode showing luteolin-*C*-pentoside-*C*-hexoside. |
| 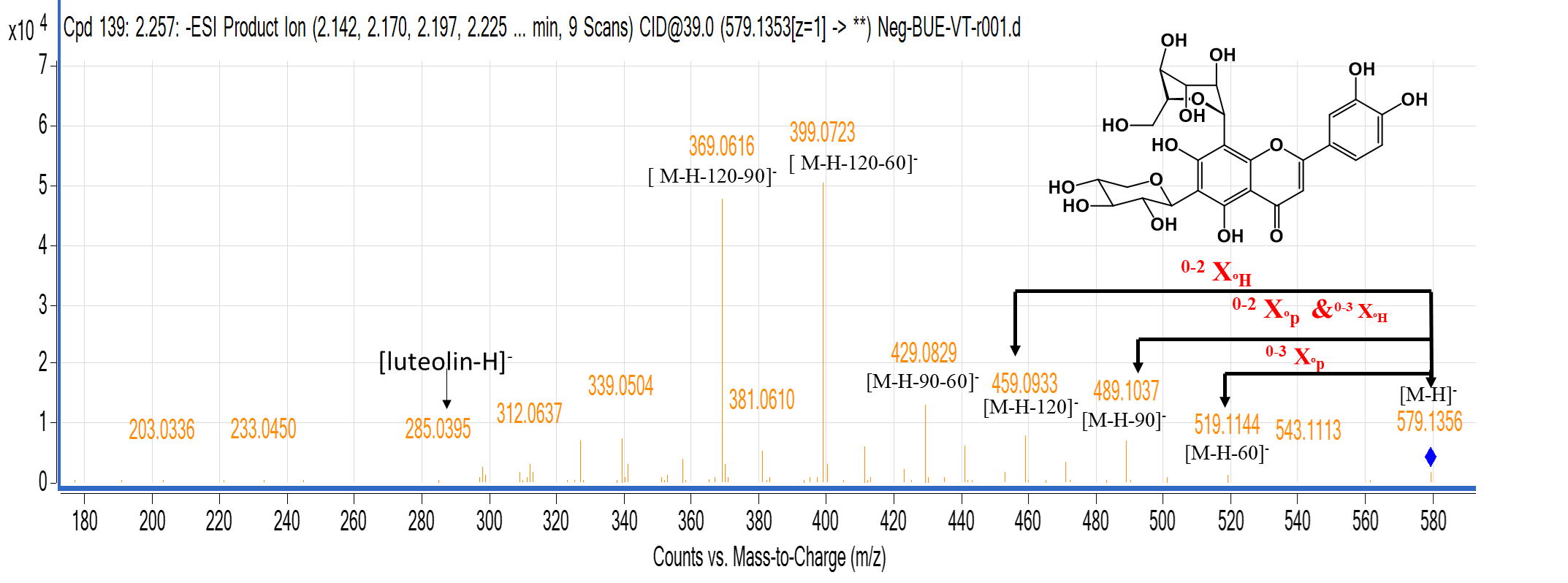 |
| ESI-MS/MS spectrum of peak (32) *via* negative ionization mode showing luteolin-*C*-(*O*-deoxyhexosyl) hexoside. |
| 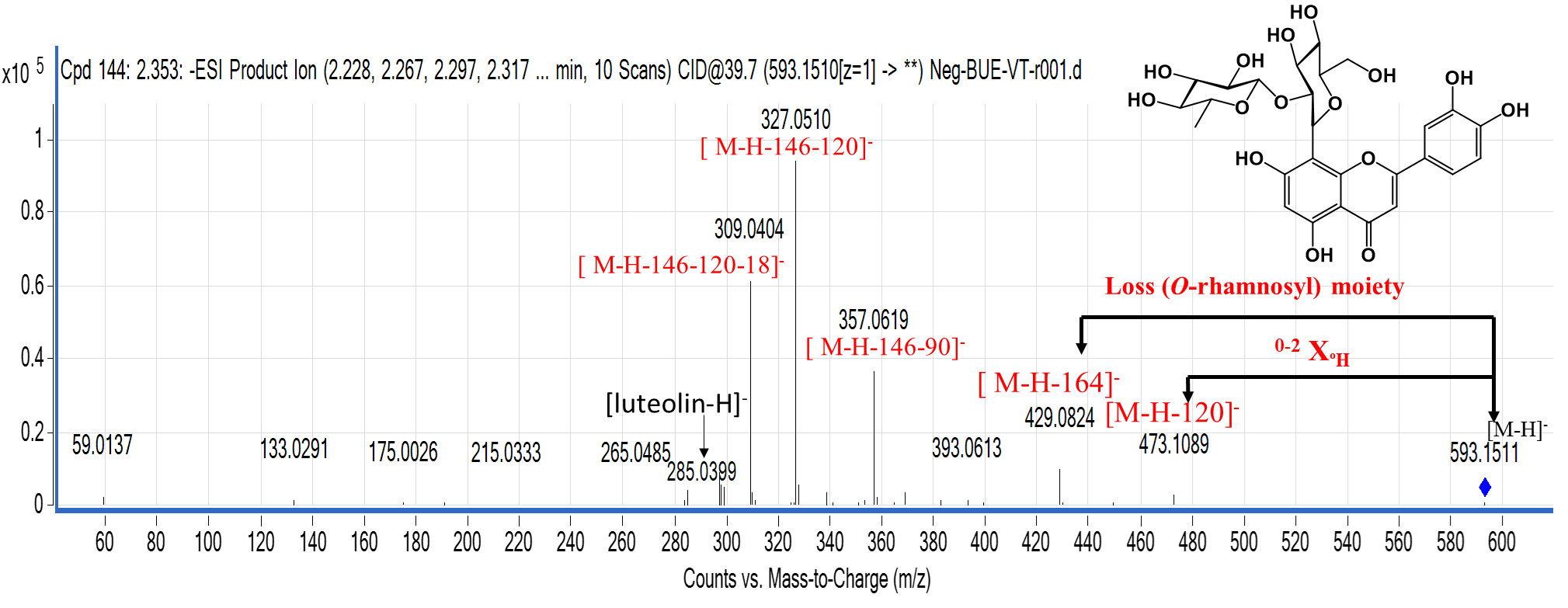 |
| ESI-MS/MS spectrum of peak (33) *via* negative ionization mode showing luteolin-*C*-(*O*-pentosyl)hexoside. |
| 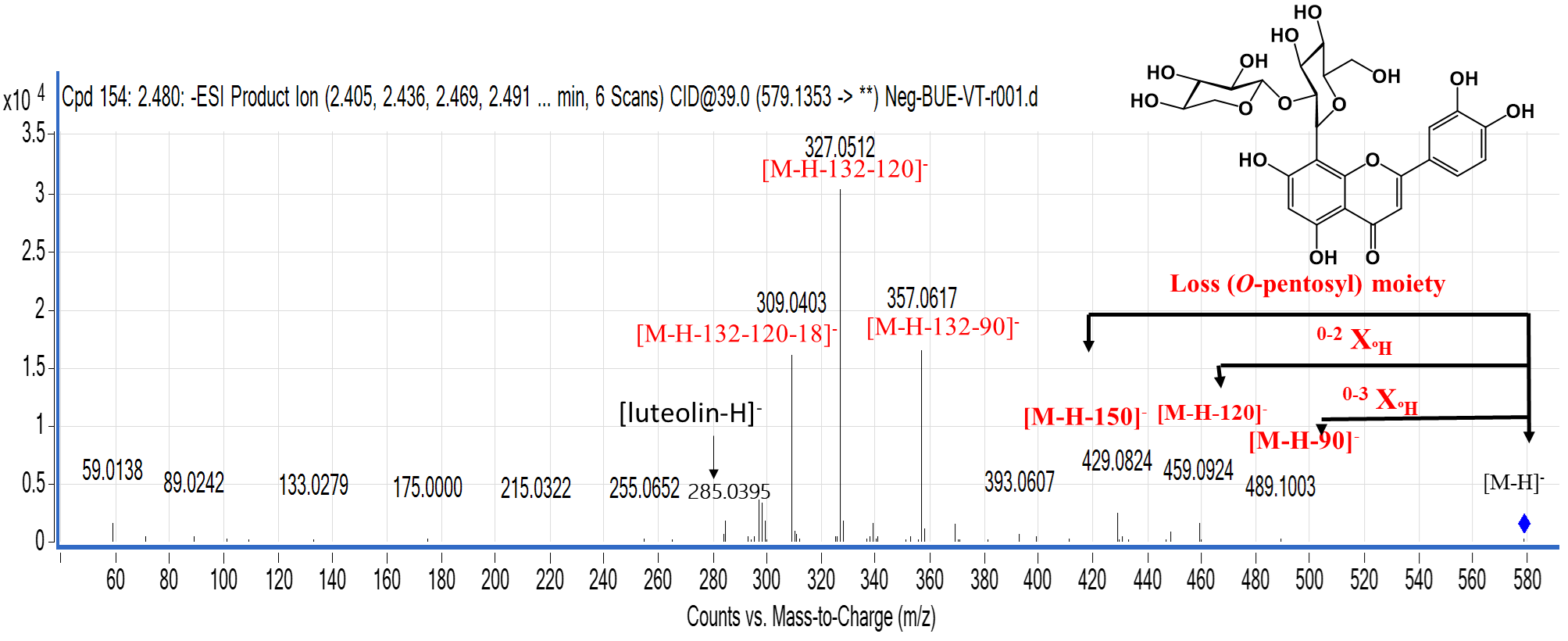 |
| ESI-MS/MS spectrum of peak (34) *via* negative ionization mode showing luteolin-*C*-hexoside dimer. |
| 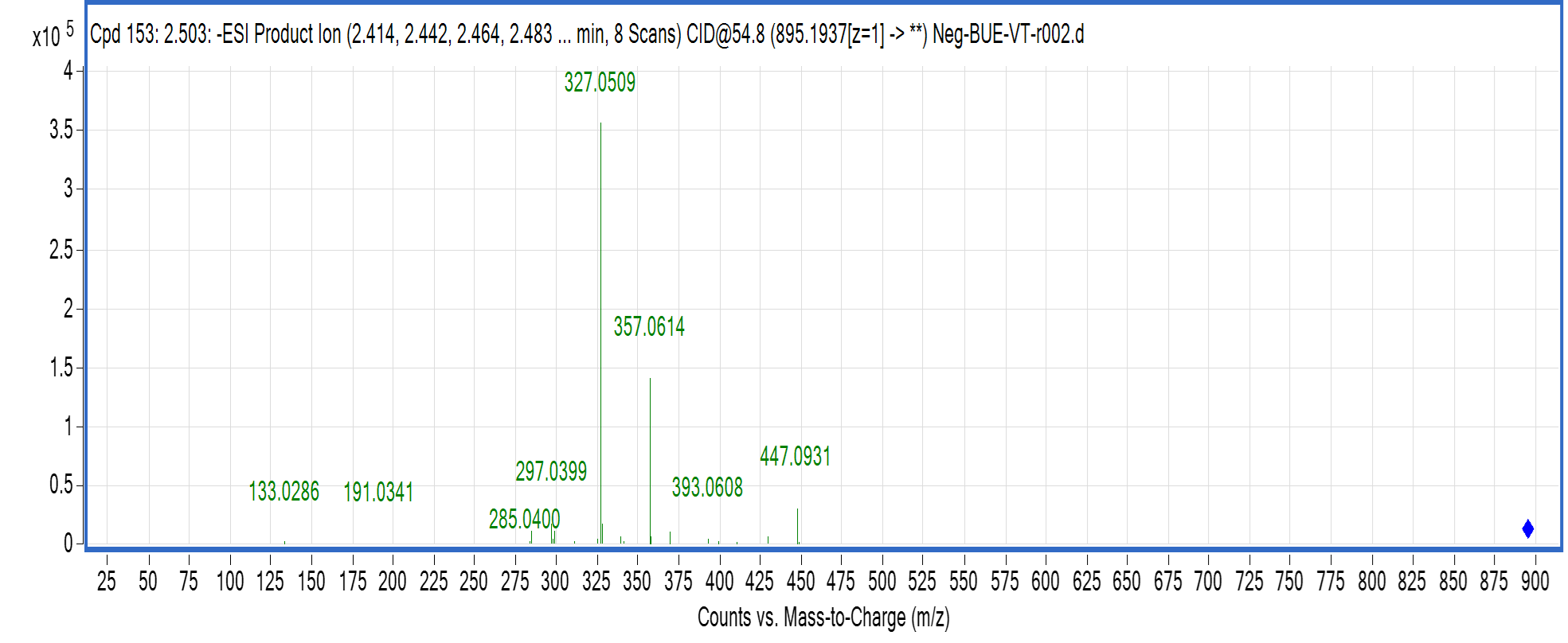 |
| 1. ESI-MS/MS spectrum of peak (**35**) *via* negative ionization mode showing luteolin-*C*-hexoside (luteolin-8-*C*-glucoside, orientin). |
| 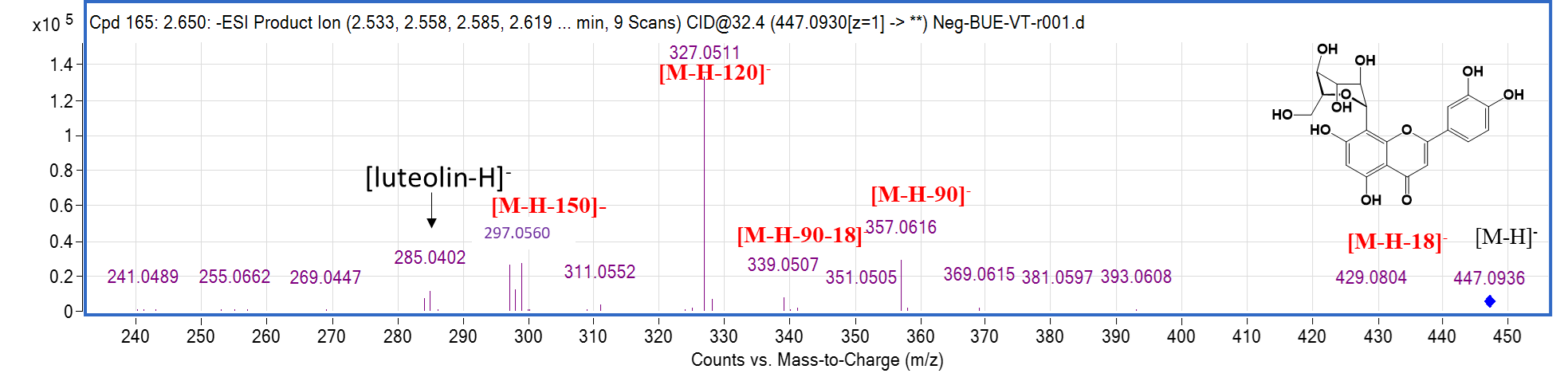 |
| ESI-MS/MS spectrum of peak (36) *via* negative ionization mode showing apigenin-*C-*(*O*-deoxyhexosyl)hexoside. |
| 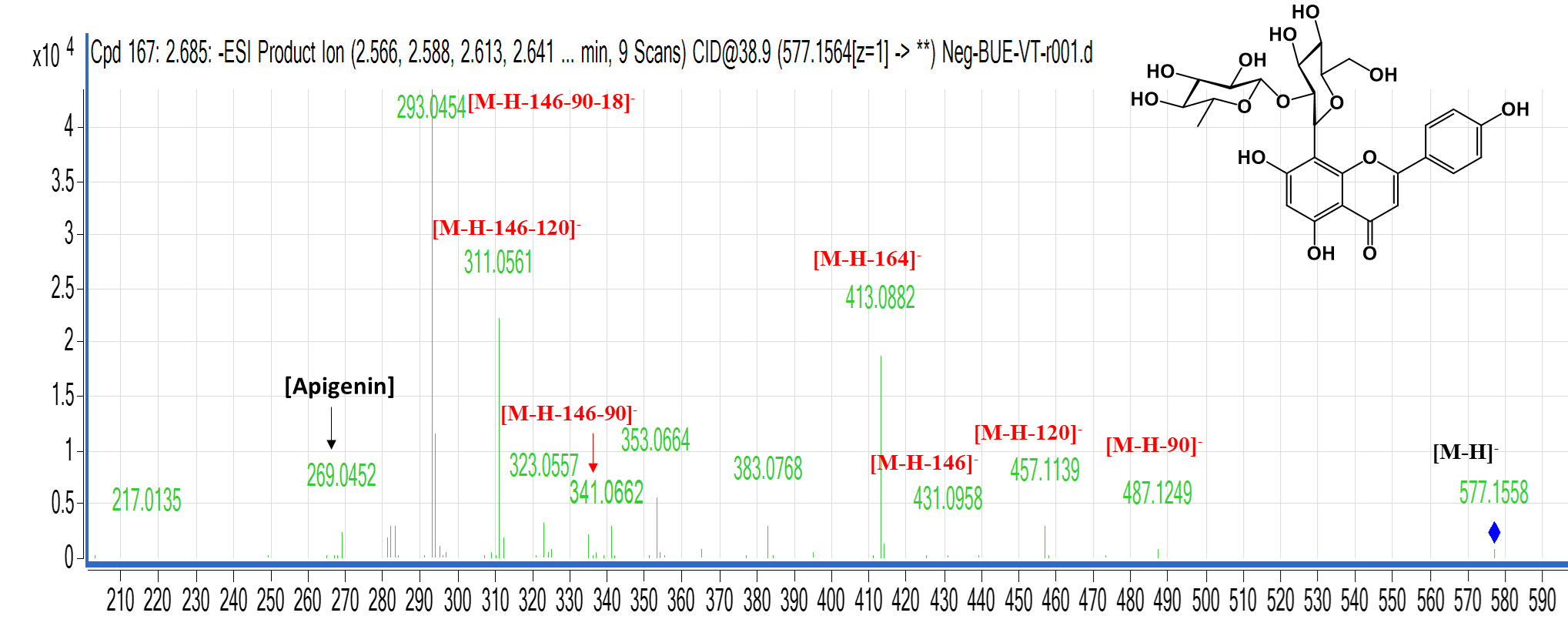 |
| ESI-MS/MS spectrum of peak (37) *via* negative ionization mode showing chrysoeriol-*C-* (*O*-deoxyhexosyl)hexoside. |
| 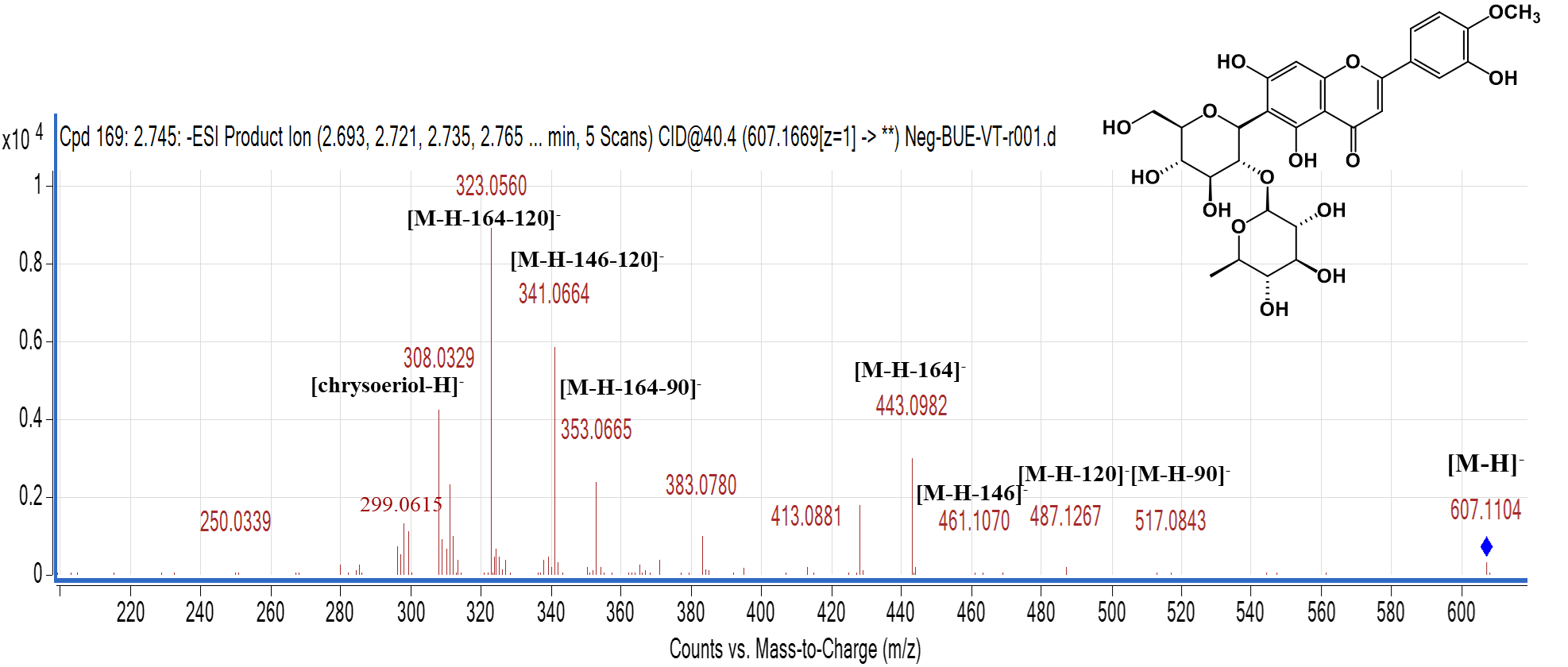 |
| 1. ESI-MS/MS spectrum of peak (**38**) *via* negative ionization mode showing apigenin-*C*-hexoside (apigenin-8-*C*-glucoside, vitexin). |
| 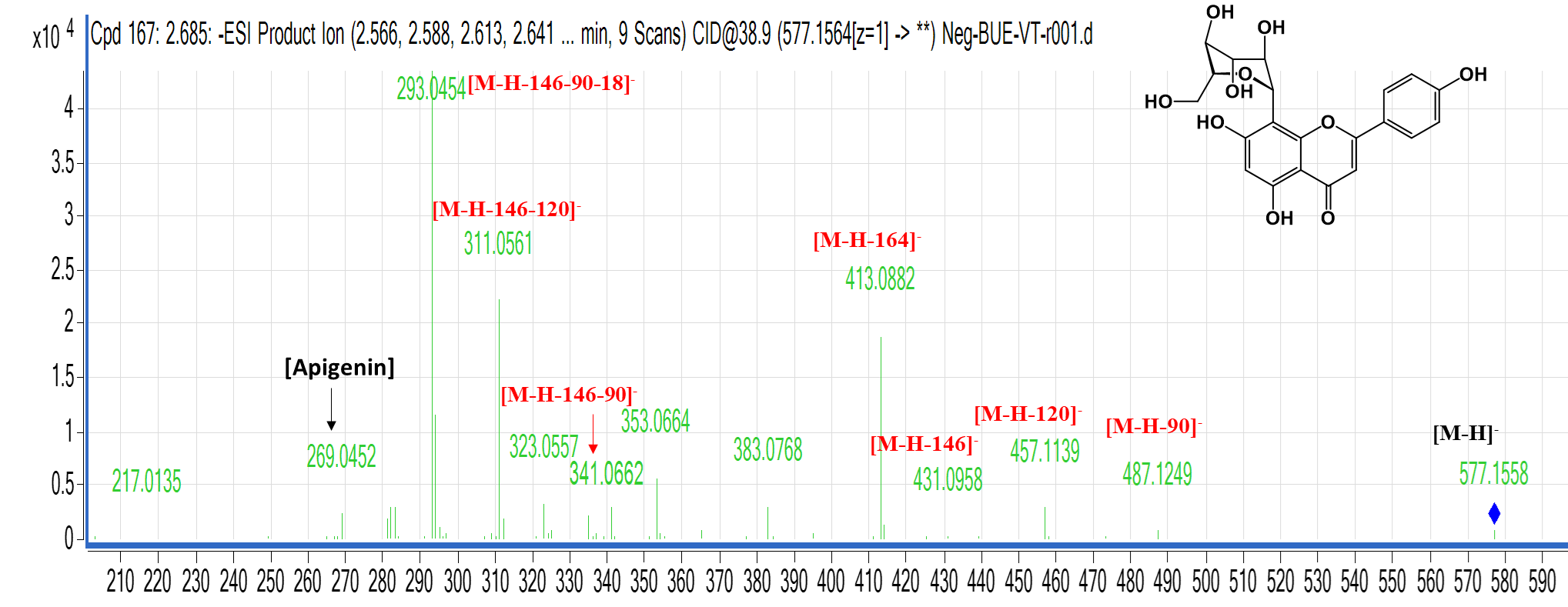 |
| ESI-MS/MS spectrum of peak (39) *via* negative ionization mode showing vanilloyl-*C-(O*-pentosyl)hexosyl-luteolin. |
| 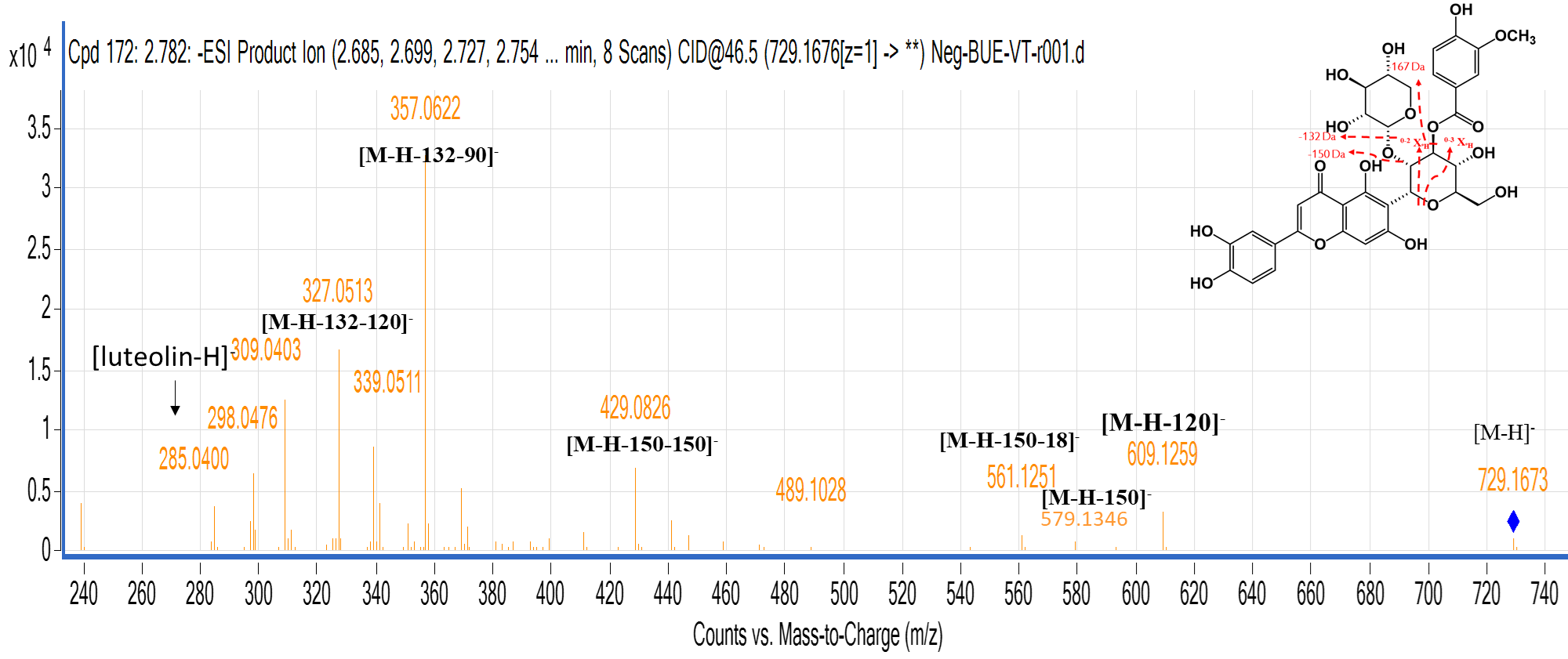 |
| ESI-MS/MS spectrum of peak (40) *via* negative ionization mode showing methoxy luteolin-*C*-hexoside. |
| 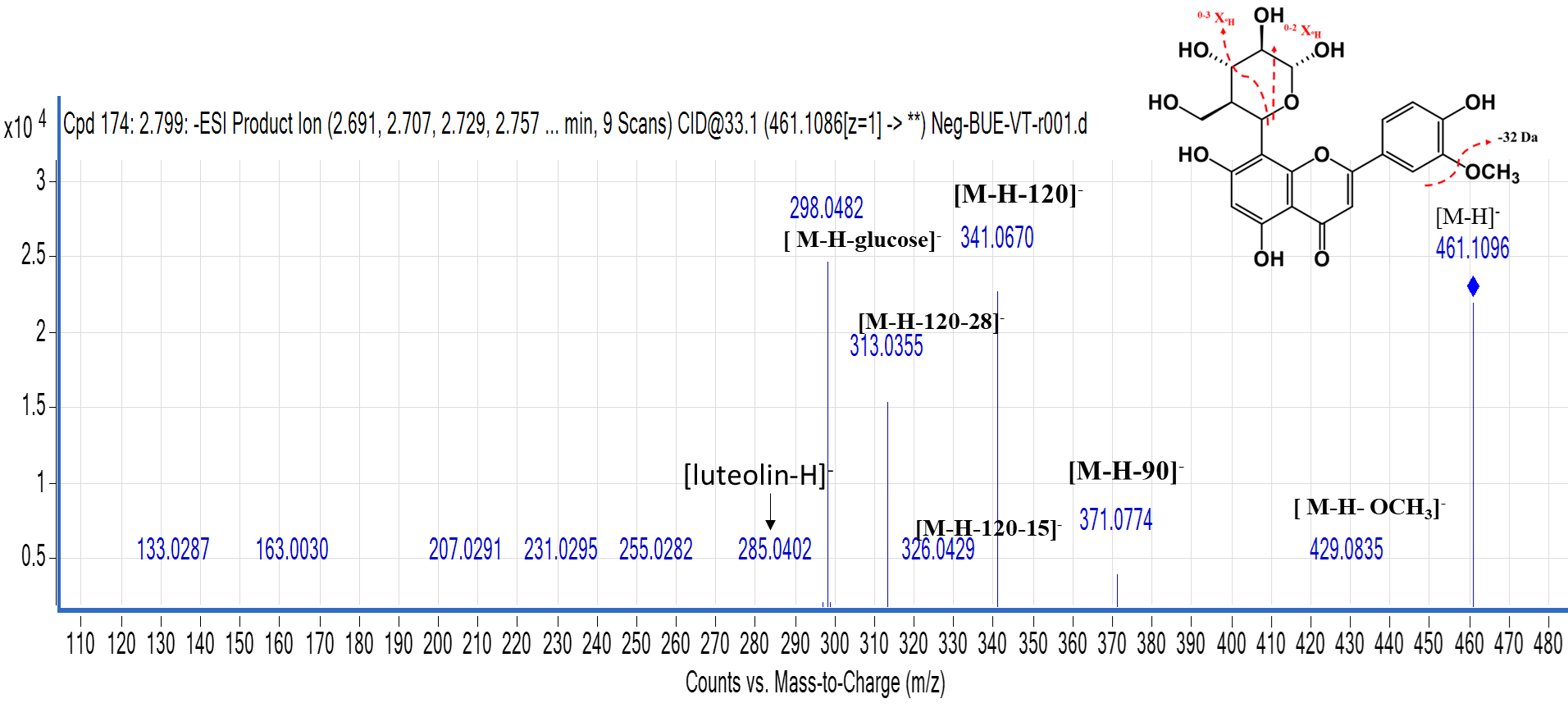 |
| ESI-MS/MS spectrum of peak (41) *via* negative ionization mode showing luteolin-*O*-(*O*-deoxyhexosyl)hexoside. |
| 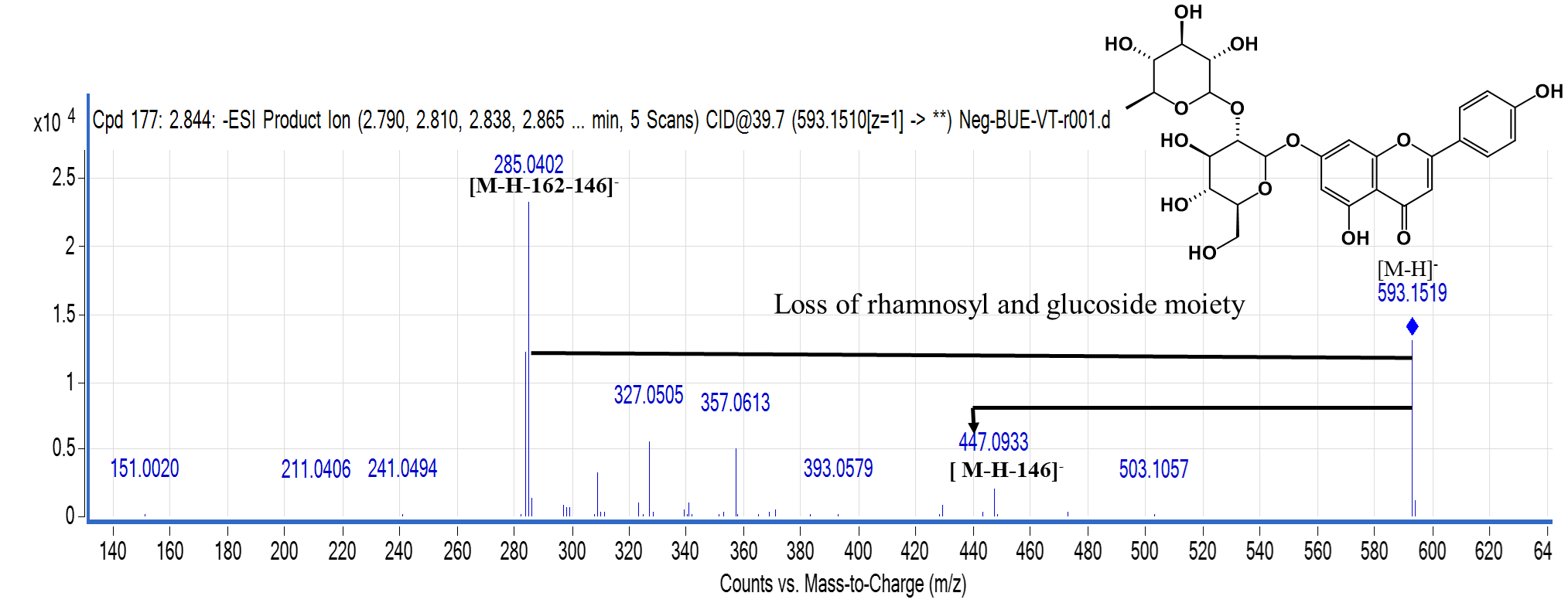 |
| ESI-MS/MS spectrum of peak (42) *via* negative ionization mode showing Azelaic acid. |
| 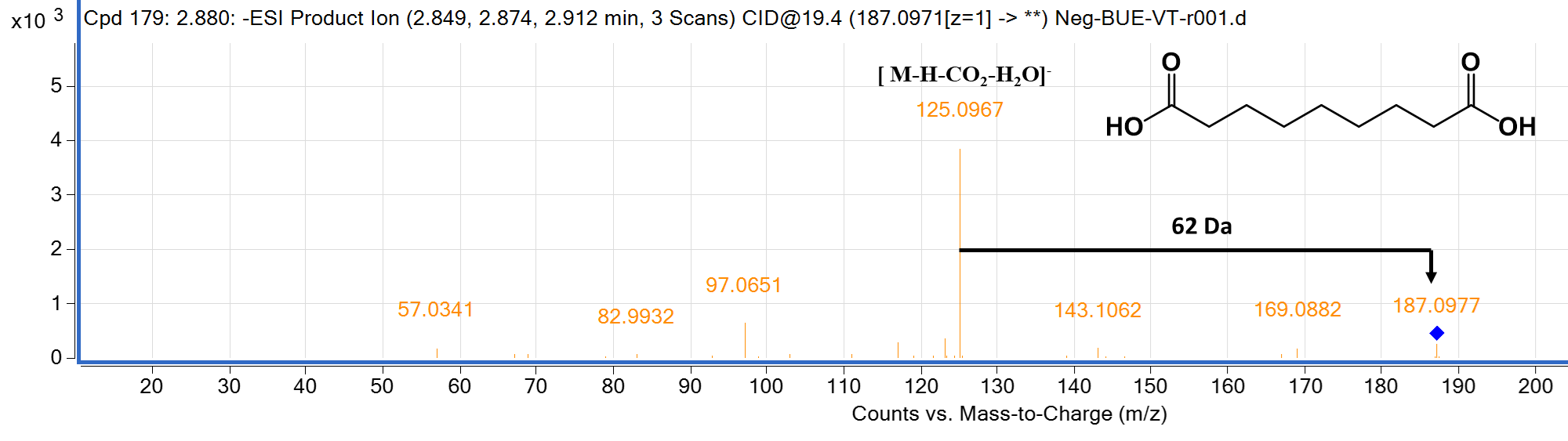 |
| ESI-MS/MS spectrum of peak (43) *via* negative ionization mode showing *O*-Caffeoyl-*O*-syringoylquinic acid. |
| 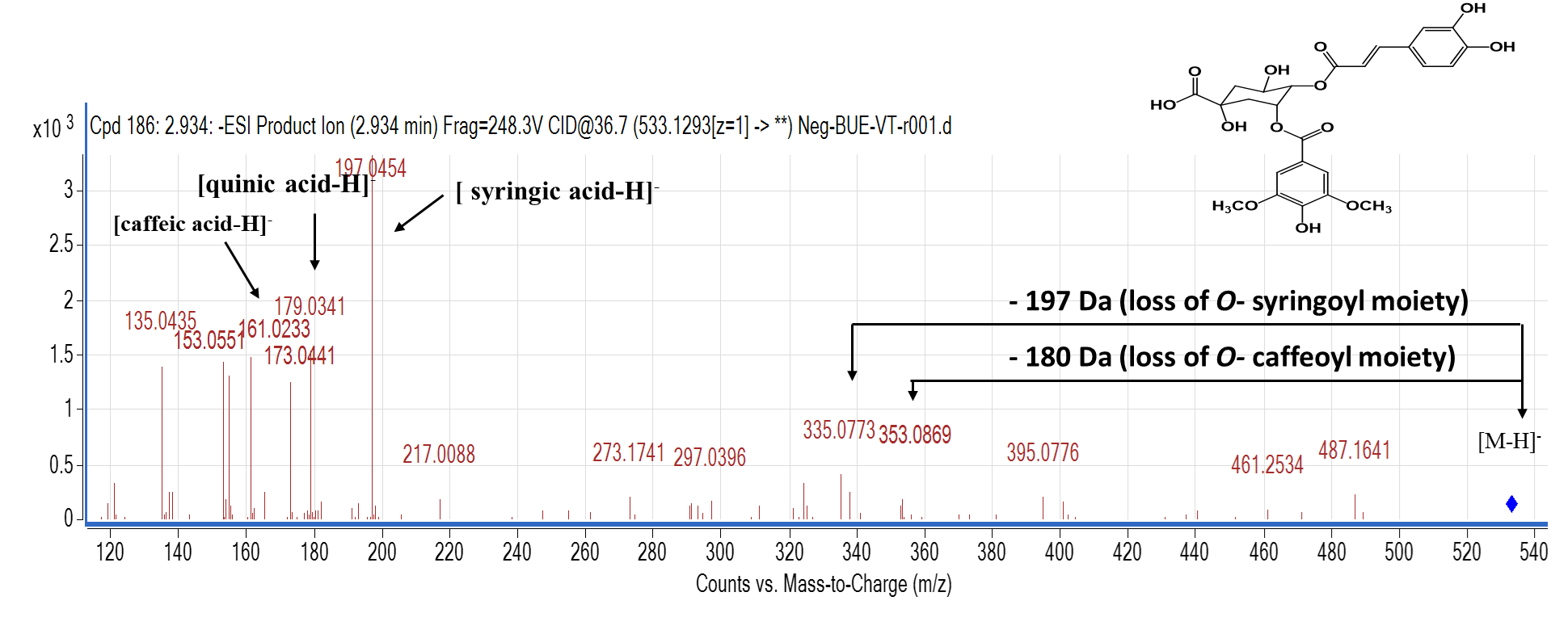 |
| ESI-MS/MS spectrum of peak (44) *via* negative ionization mode showing vanilloyl-*C*-(*O*-pentosyl) hexosyl- chrysoeriol. |
| 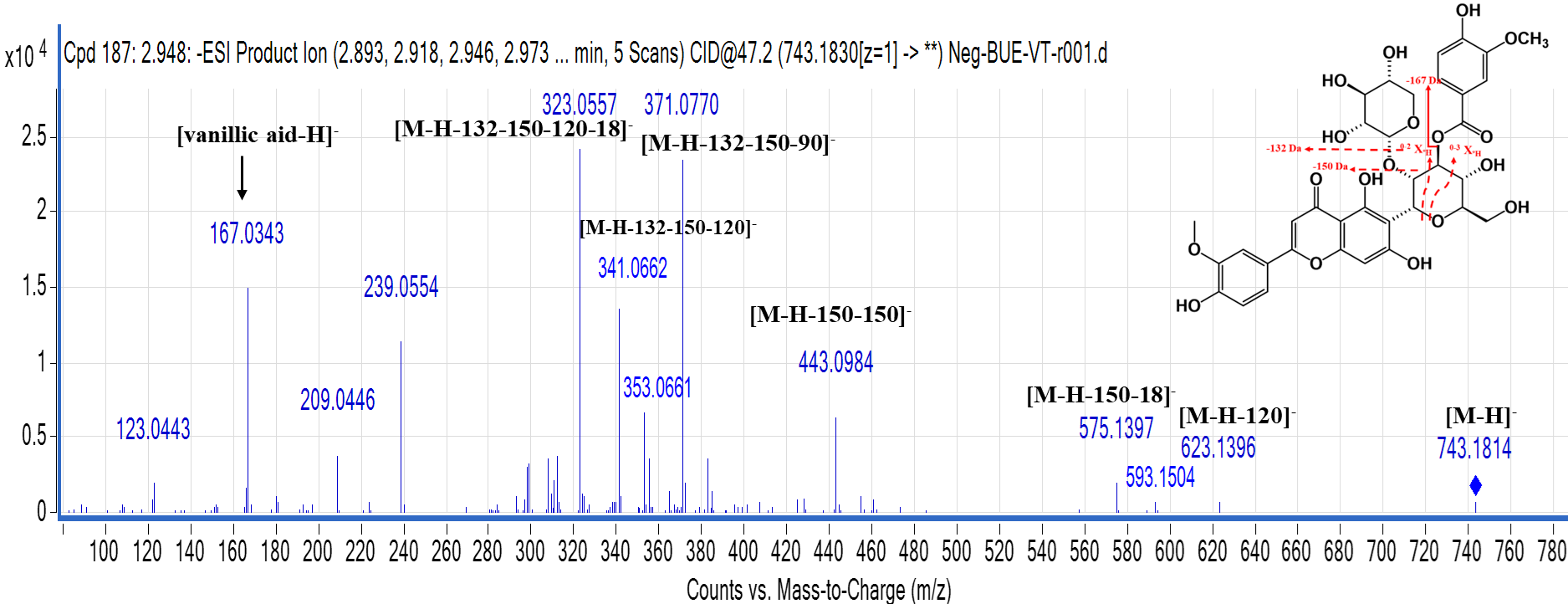 |
| ESI-MS/MS spectrum of peak (45) *via* negative ionization mode showing vanilloyl-*C*-(*O*-pentosyl) hexosyl-apigenin. |
| 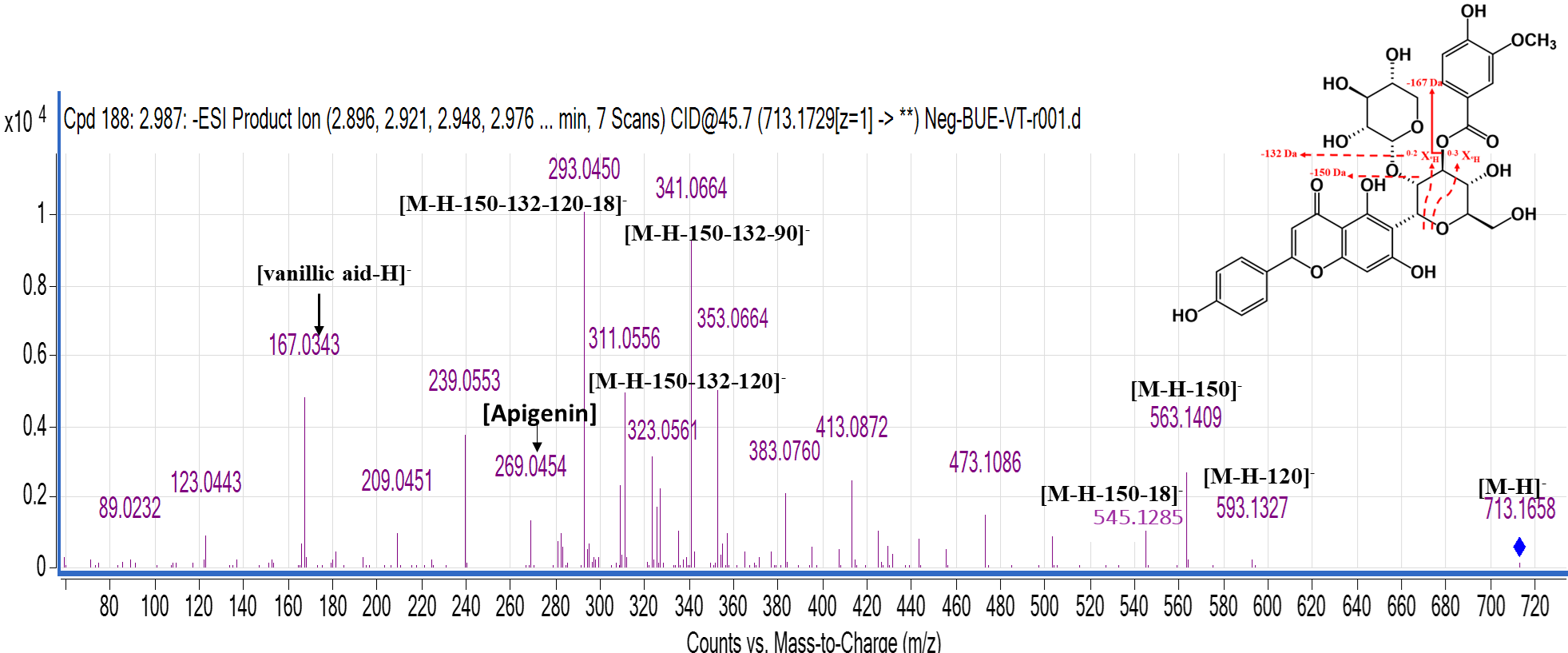 |
| ESI-MS/MS spectrum of peak (46) *via* negative ionization mode showing *O*-Caffeoyl-*O*-vanilloylquinic acid. |
| 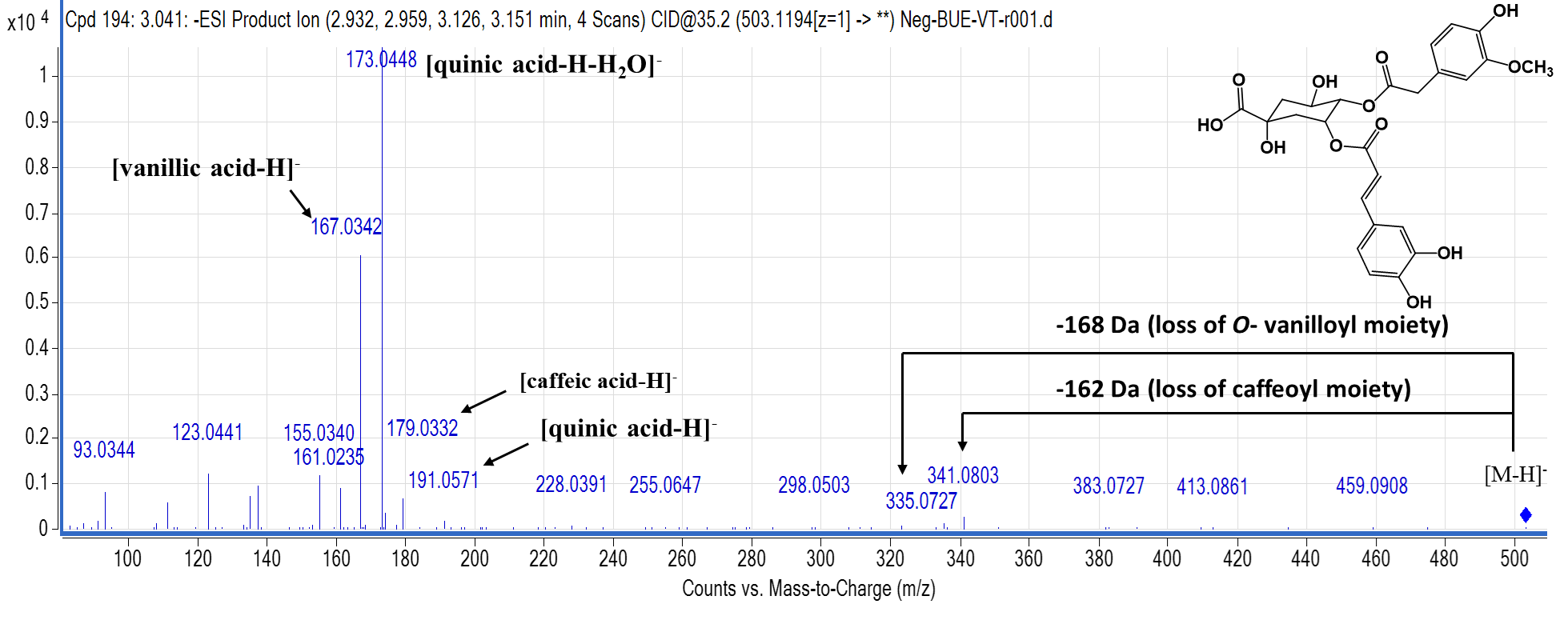 |
| ESI-MS/MS spectrum of peak (47) *via* negative ionization mode showing di-*O*-caffeoylquinic acid. |
| 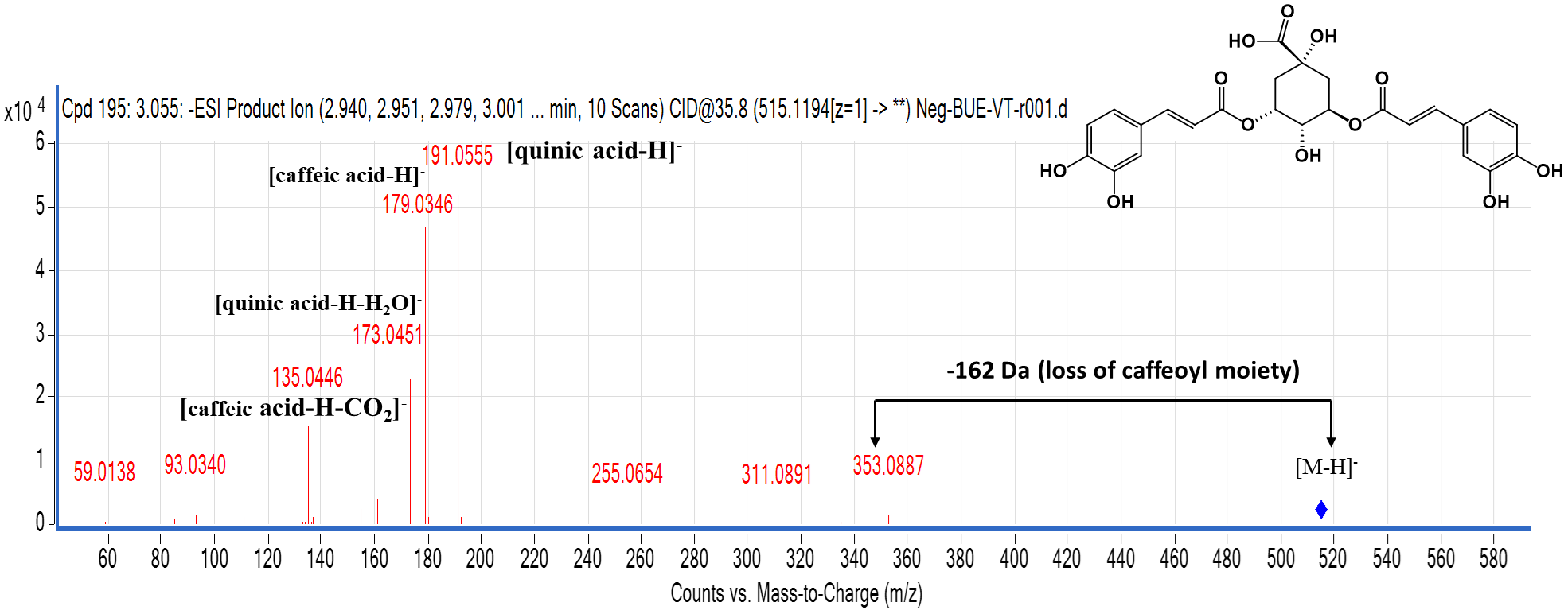 |
| ESI-MS/MS spectrum of peak (48) *via* negative ionization mode showing caffeic acid. |
| 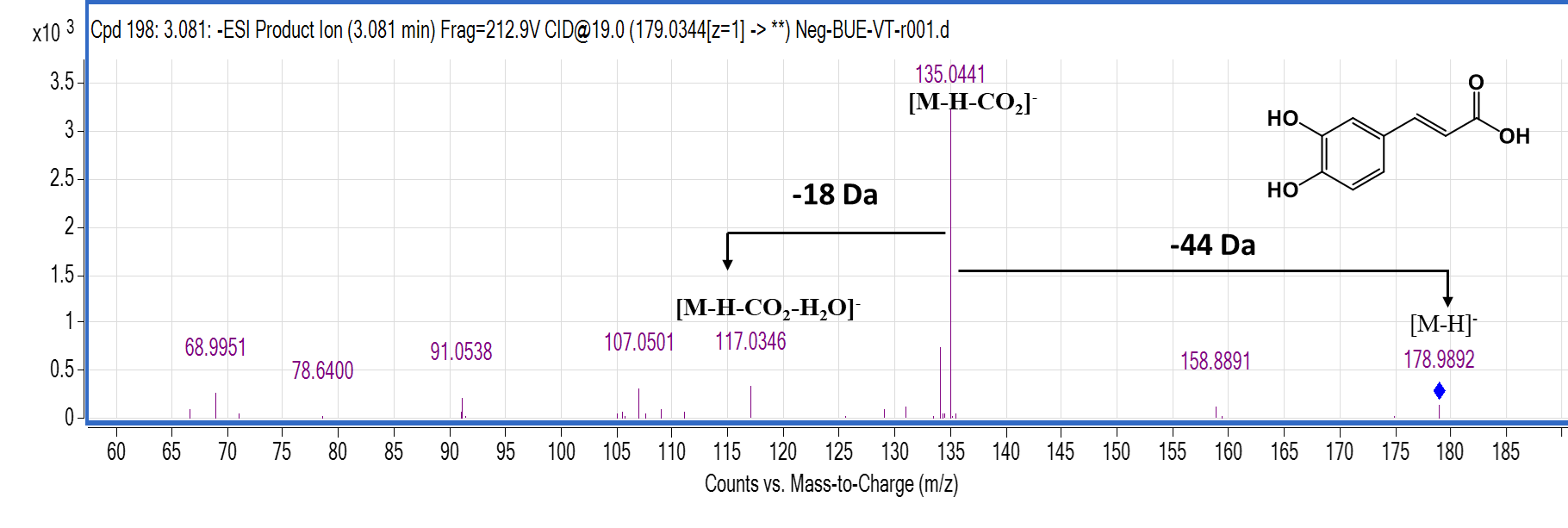 |
| ESI-MS/MS spectrum of peak (49) *via* negative ionization mode showing shikimic acid. |
| 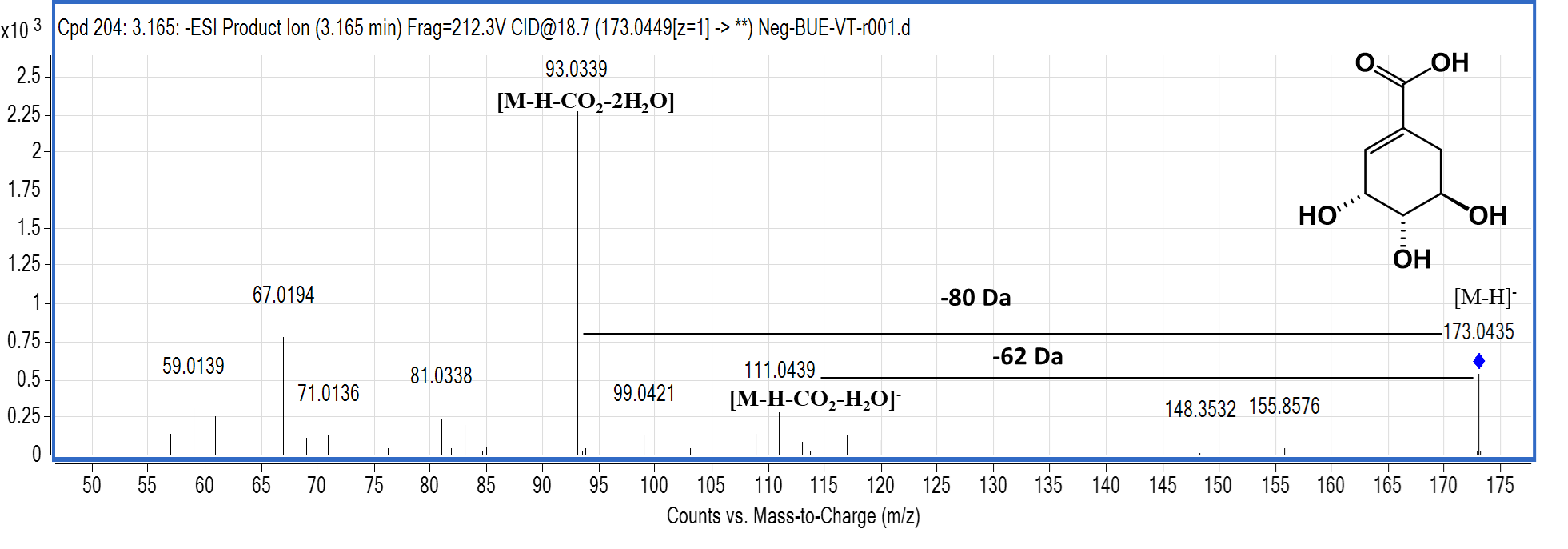 |
| 1. ESI-MS/MS spectrum of peak (**50**) *via* negative ionization mode showing *O*-*p*-hydroxybenzoyl-*O*-caffeoylquinic acid. |
| .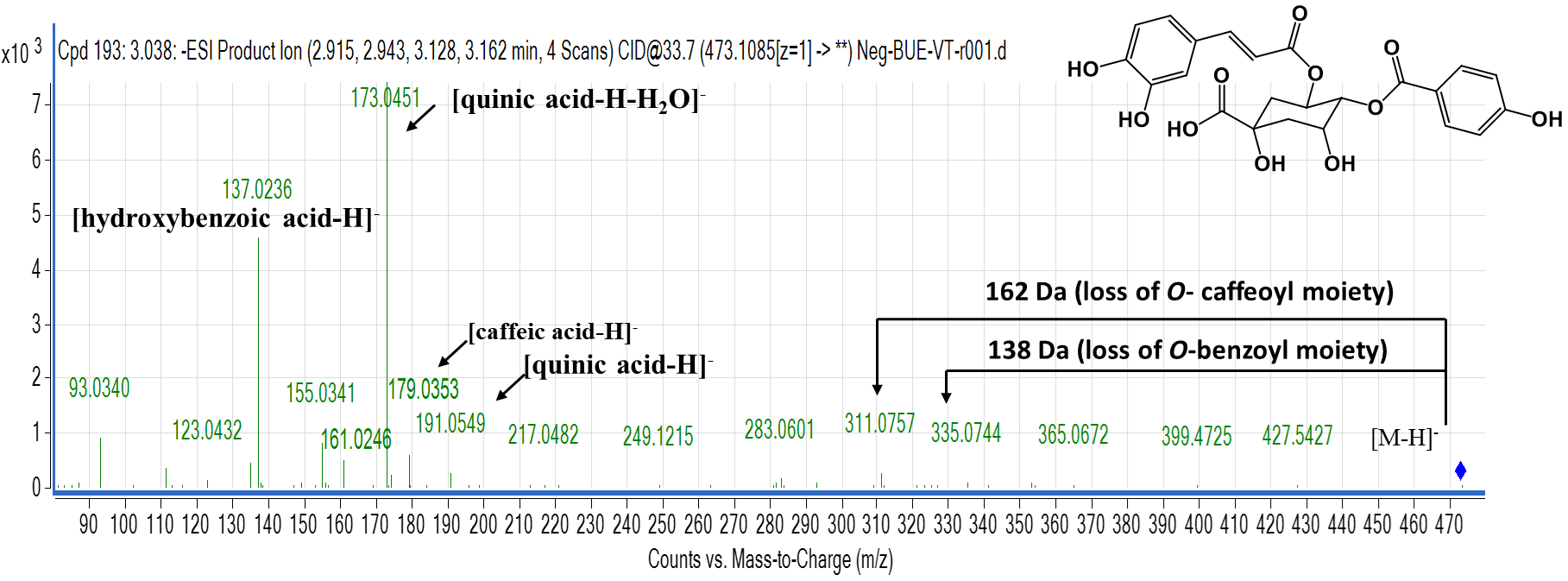 |
| ESI-MS/MS spectrum of peak (51) *via* negative ionization mode showing di-*O*-caffeoylquinic acid isomer. |
| 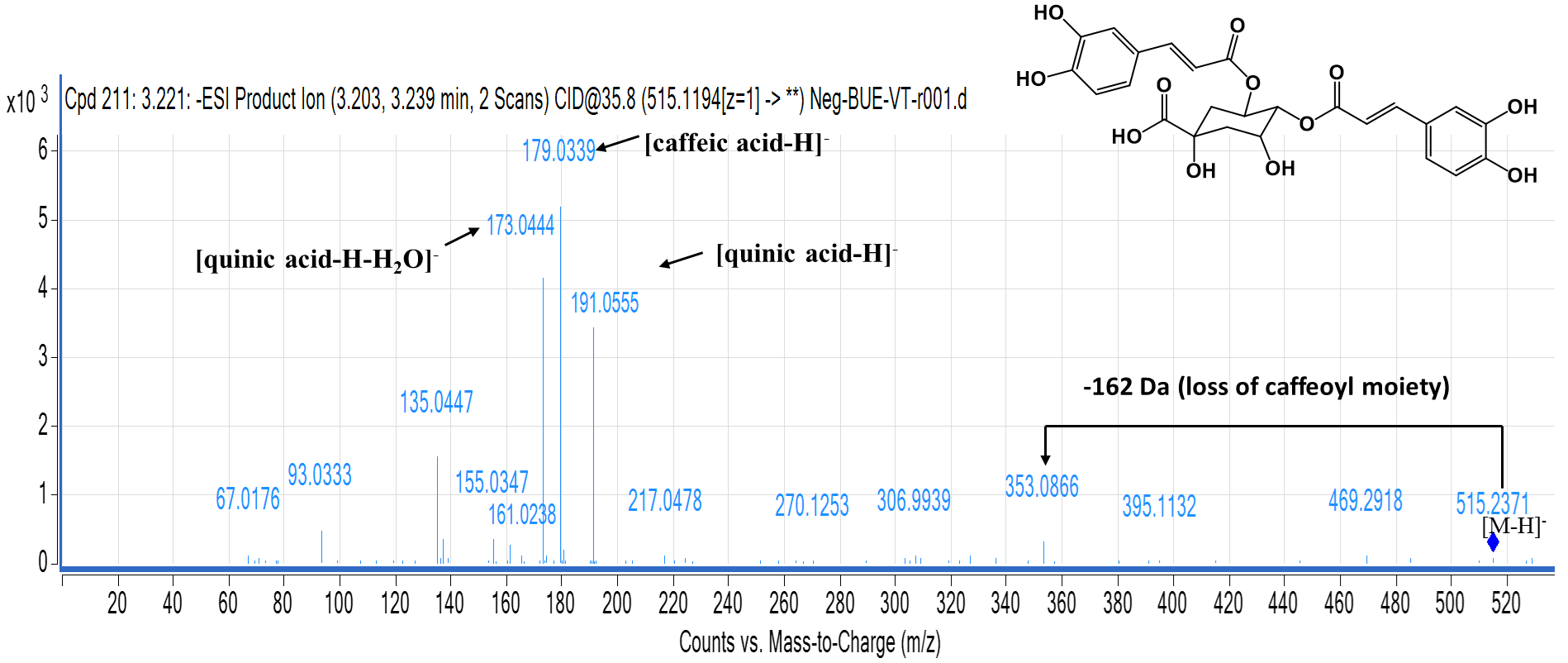 |
| 1. ESI-MS/MS spectrum of peak (**52**) *via* negative ionization mode showing apigenin-*C*-hexoside(apigenin-6-*C*-glucoside, isoovitexin). |
| 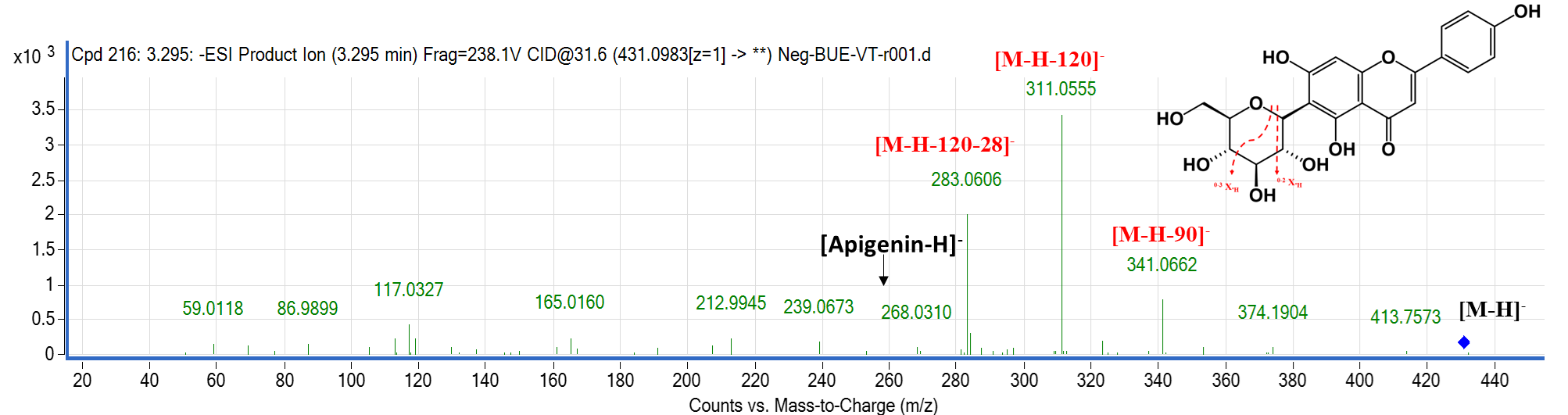 |
| ESI-MS/MS spectrum of peak (53) *via* negative ionization mode showing *O*‐*p*‐coumaroyl‐*O*‐caffeoylquinic acid. |
| 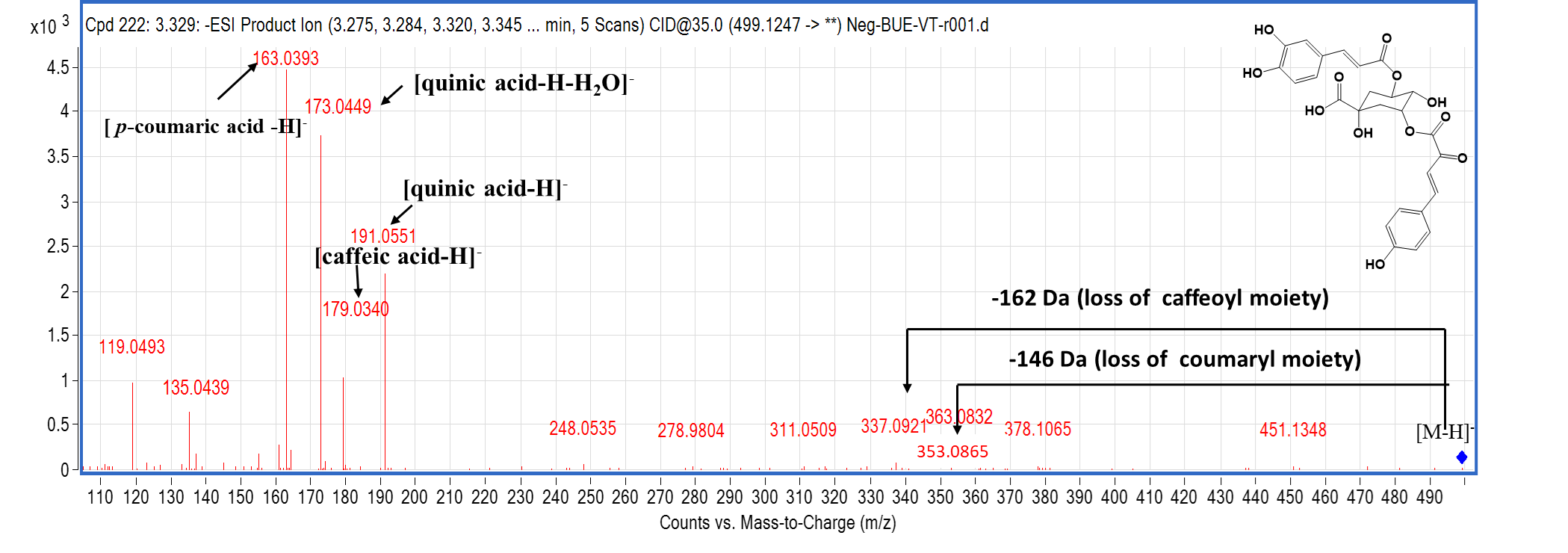 |
| 1. ESI-MS/MS spectrum of peak (**54**) *via* negative ionization mode showing methyl dicaffeoyl quinate. |
| 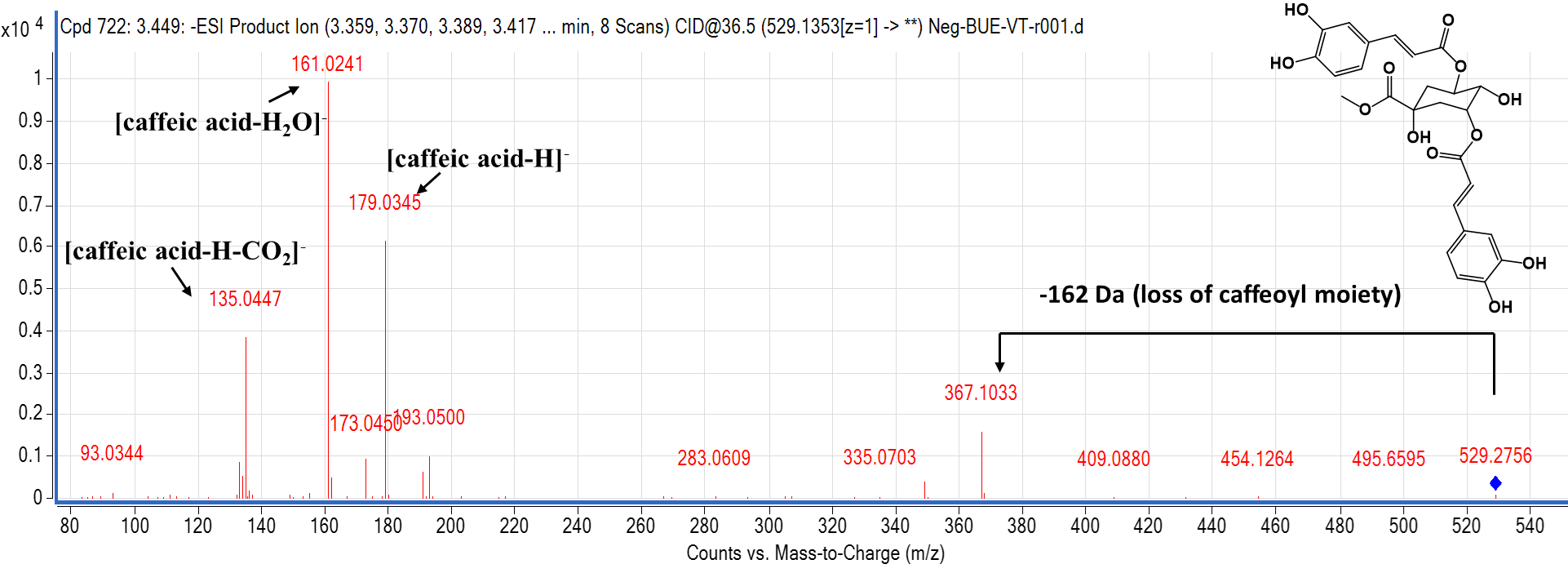 |
| ESI-MS/MS spectrum of peak (55) *via* negative ionization mode showing dicaffeoylshikimic acid. |
| 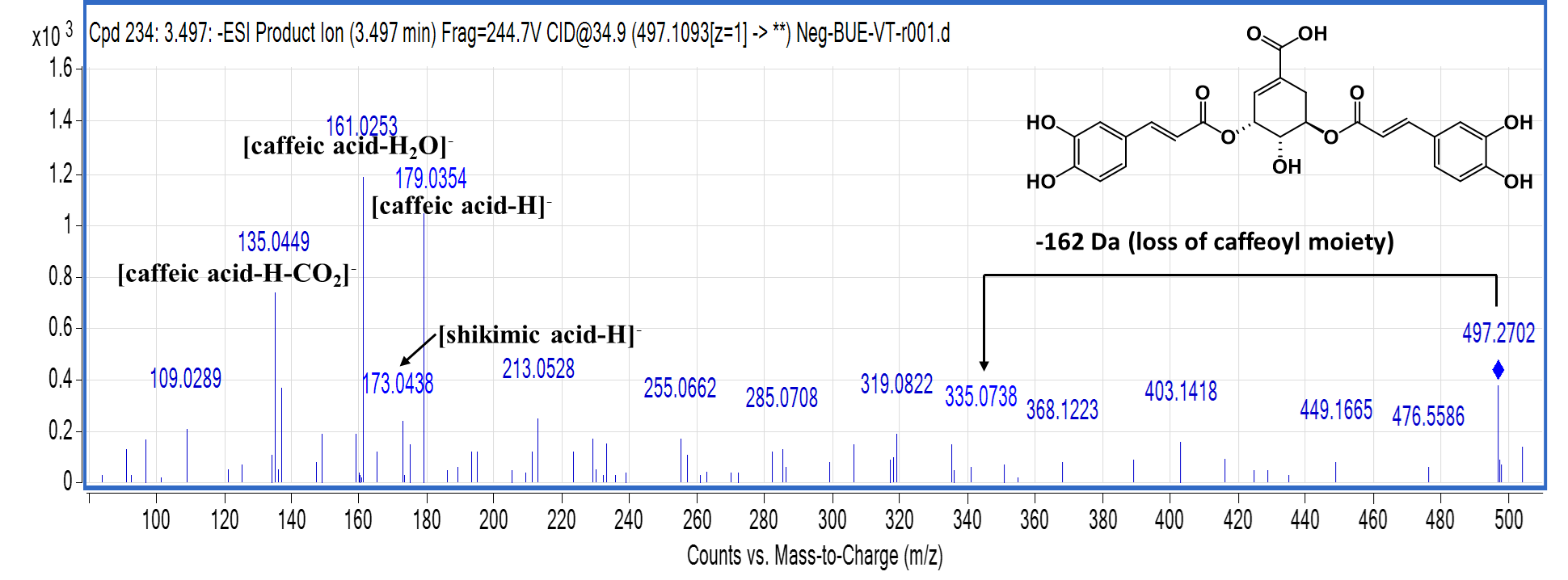 |
| ESI-MS/MS spectrum of peak (56) *via* negative ionization mode showing pomaceic acid. |
| 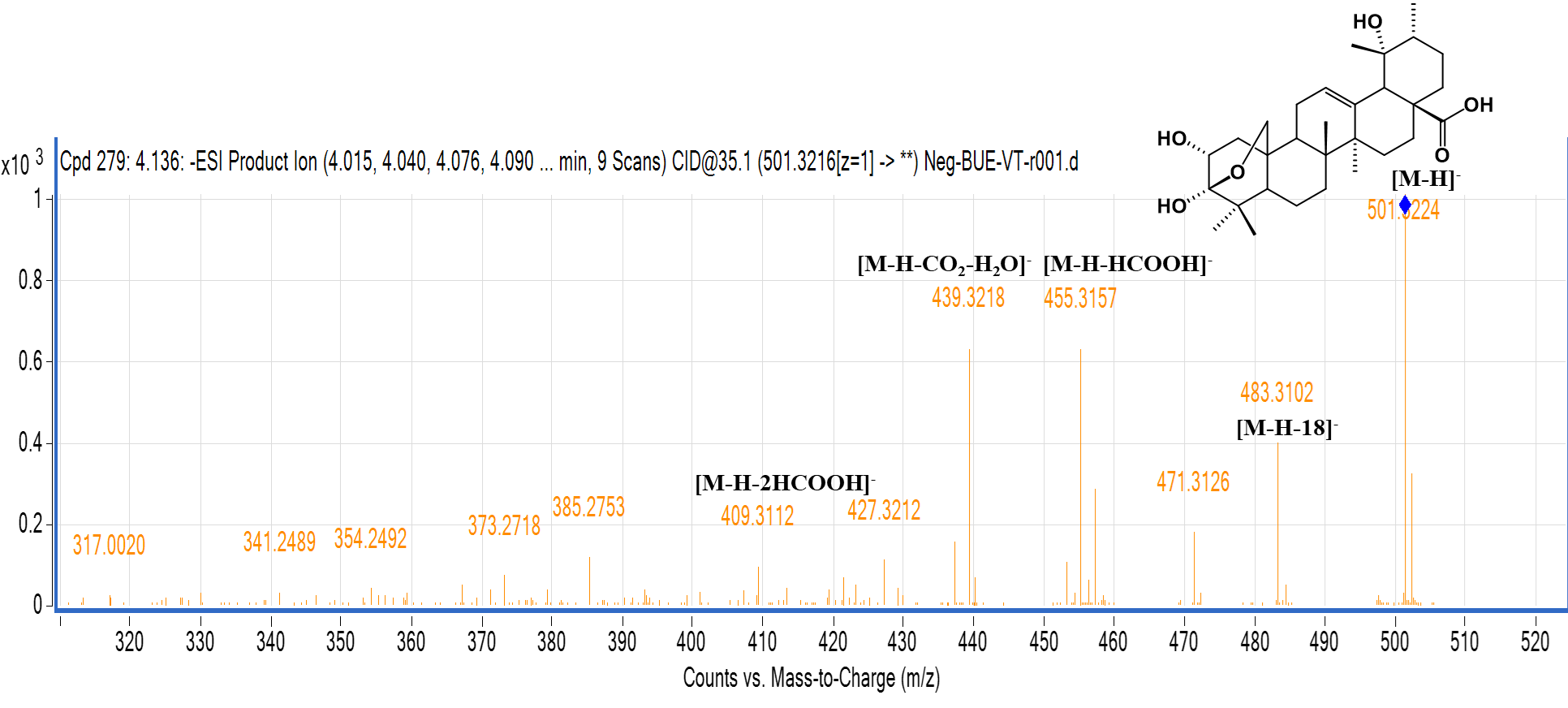 |
| ESI-MS/MS spectrum of peak (57) *via* negative ionization mode showing luteolin. |
| 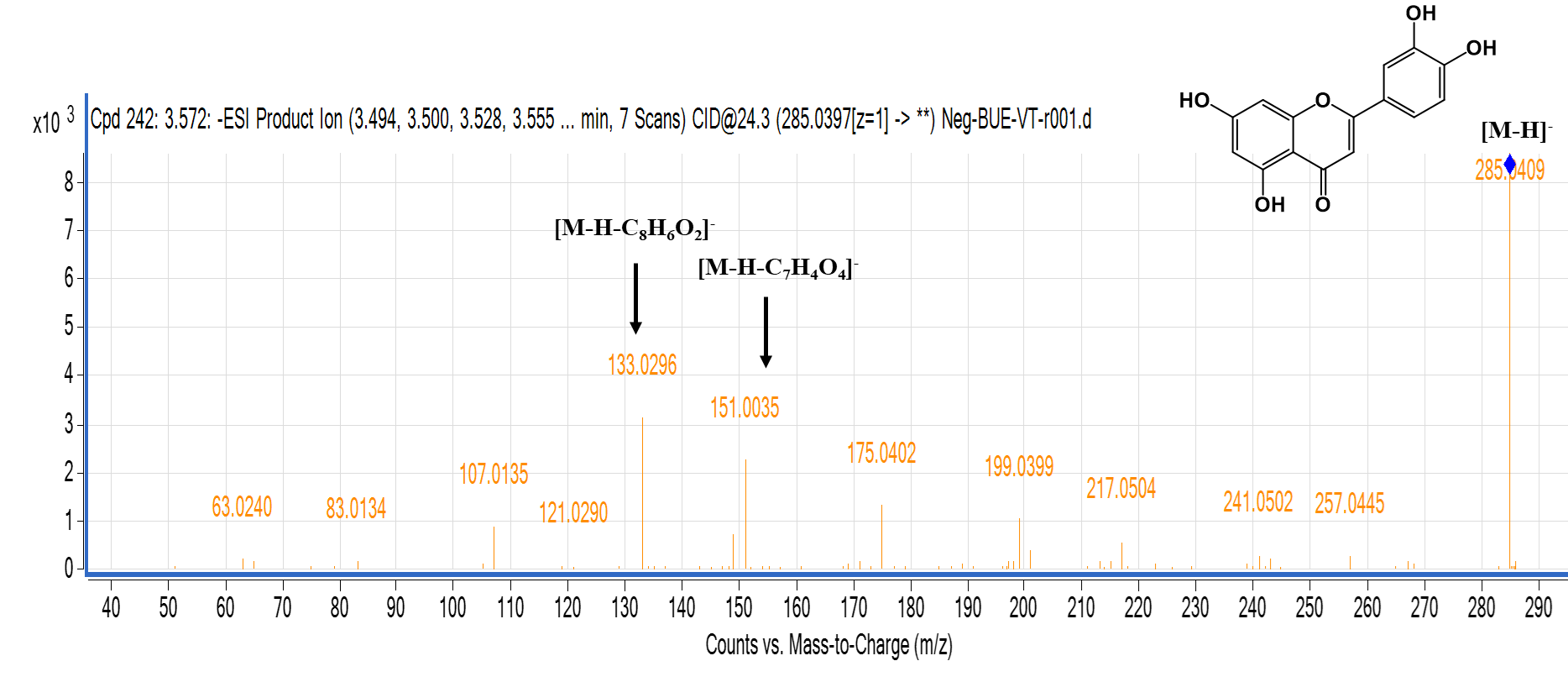 |
| ESI-MS/MS spectrum of peak (58) *via* negative ionization mode showing 9,12,13-trihydroxyoctadeca-10(E),15(Z)- dienoic acid |
| 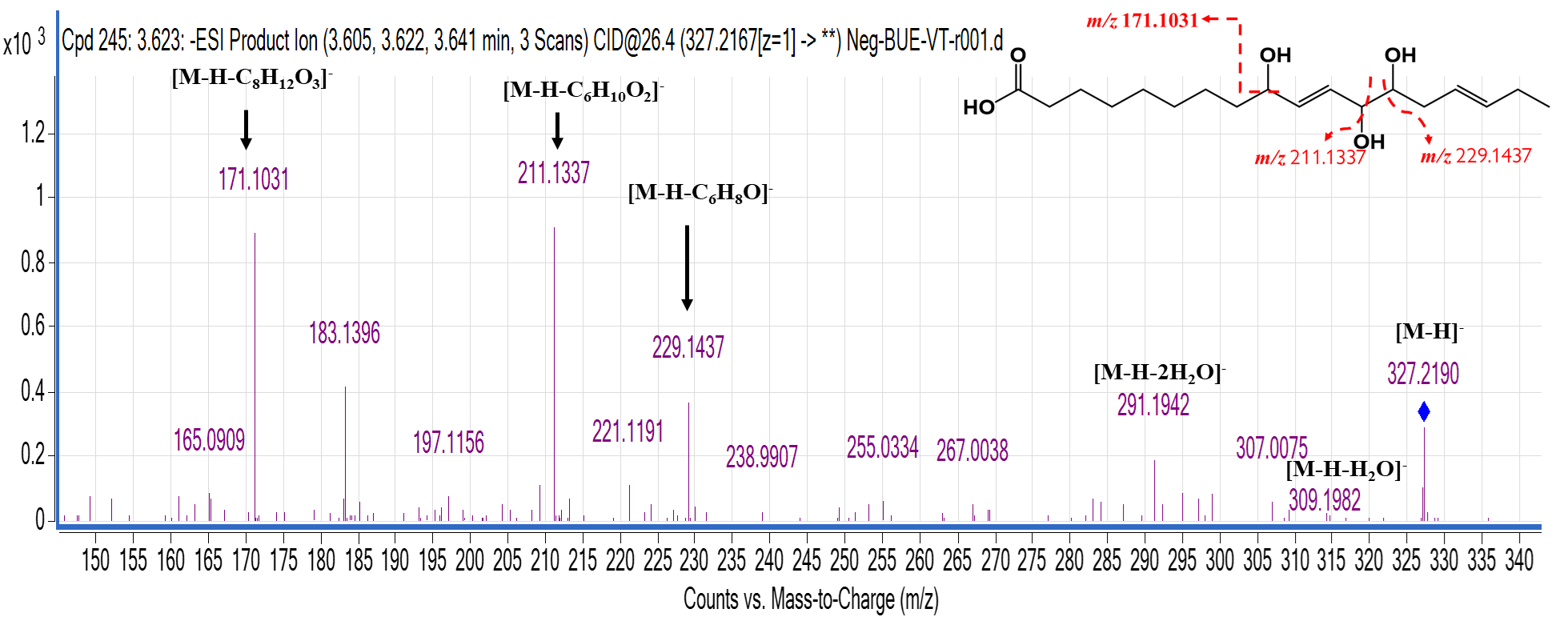 |
| ESI-MS/MS spectrum of peak (59) *via* negative ionization mode showing *O*-caffeoylquinic acid dimer. |
| 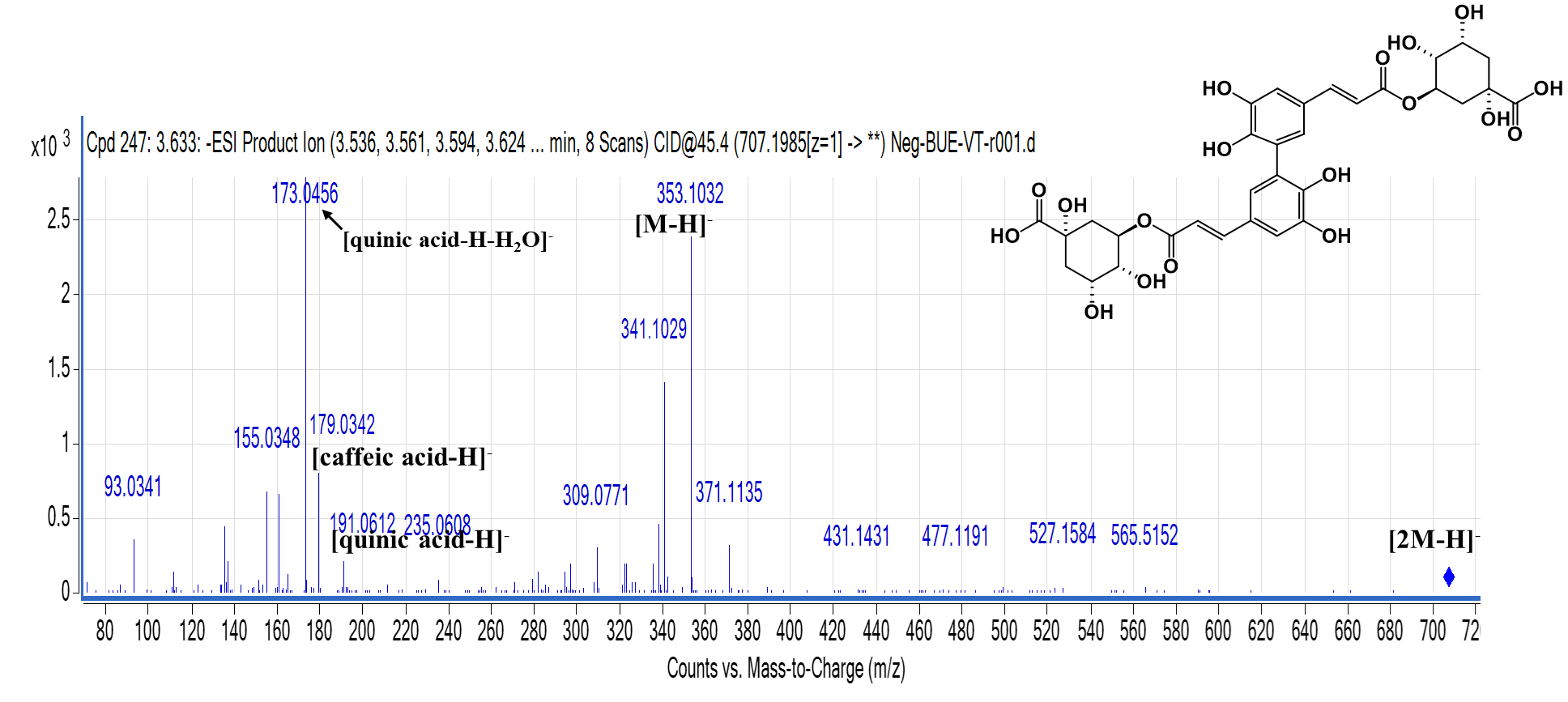 |
| ESI-MS/MS spectrum of peak (60) via negative ionization mode showing ethyl-di caffeoyl quinate. |
| 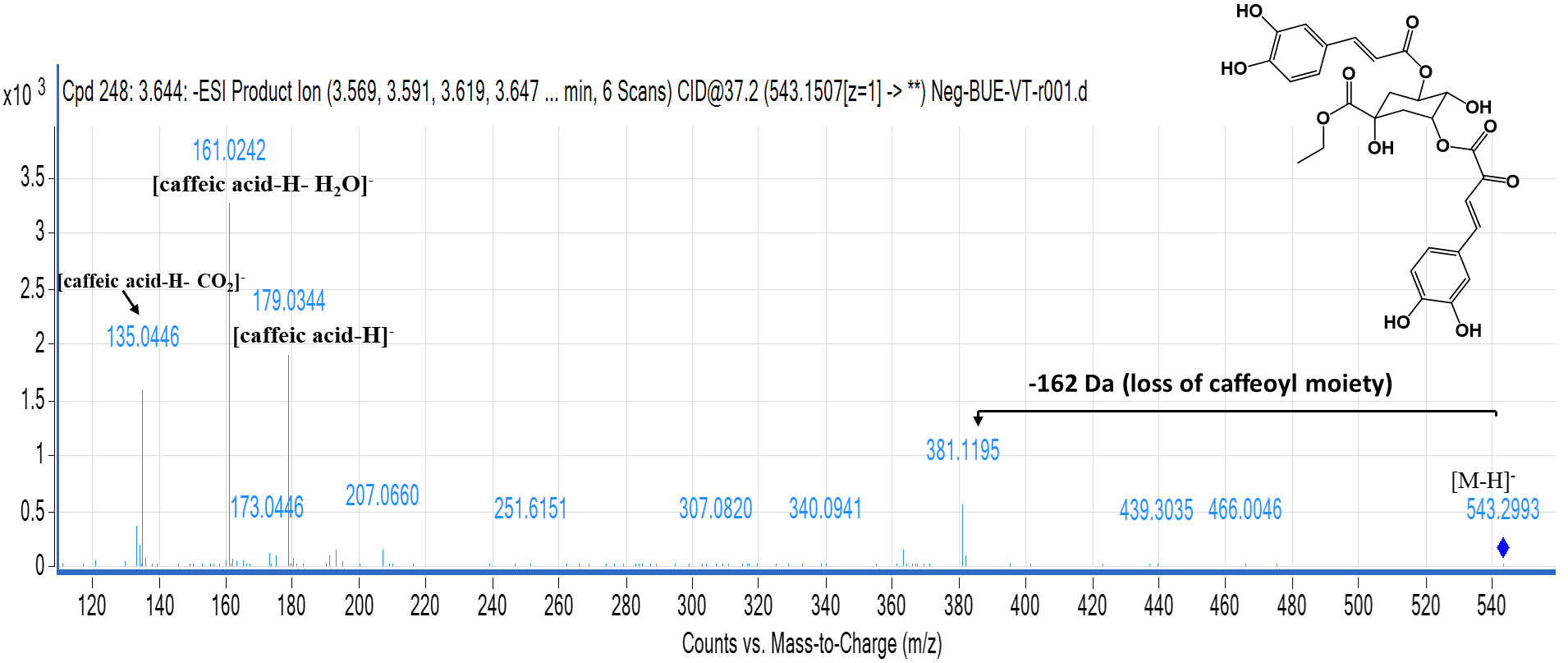 |
| ESI-MS/MS spectrum of peak (61) *via* negative ionization mode showing 9,12,13-trihydroxyoctadec-10-enoic acid. |
| 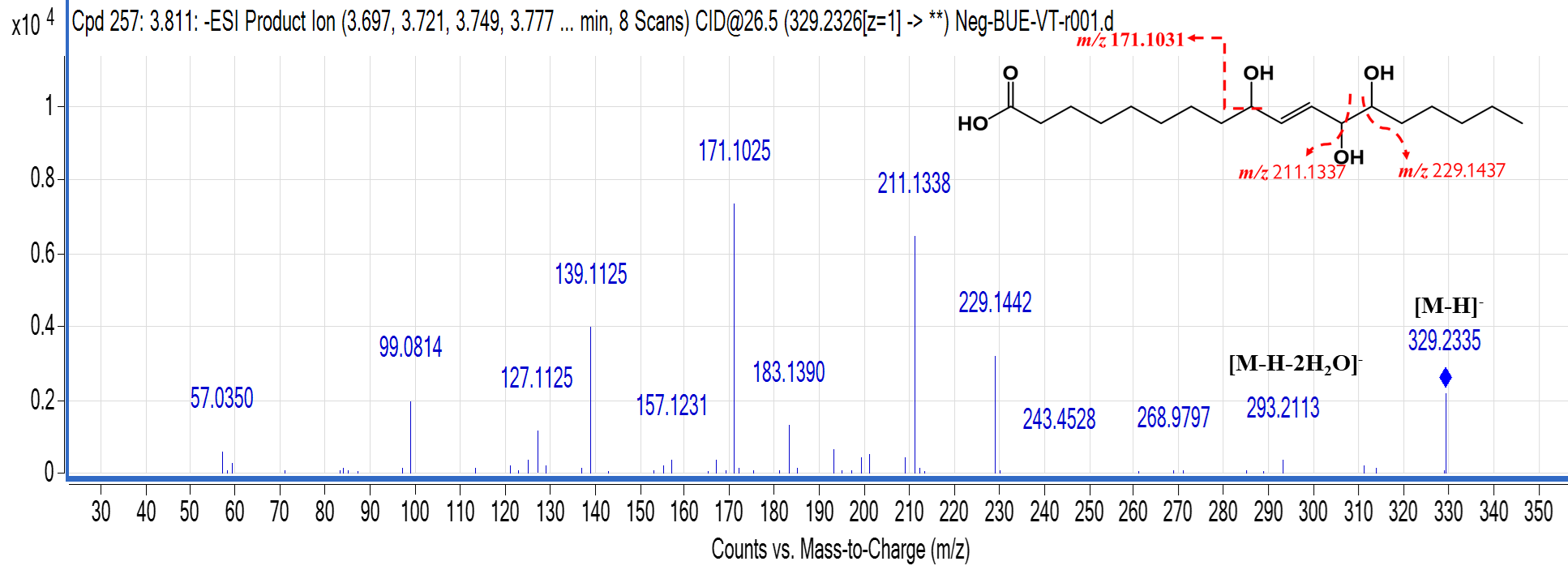 |
| 1. ESI-MS/MS spectrum of peak (**62**) *via* negative ionization mode showing luteolin-*C*-hexoside (luteolin-6-*C*-glucoside, isoorientin). |
| 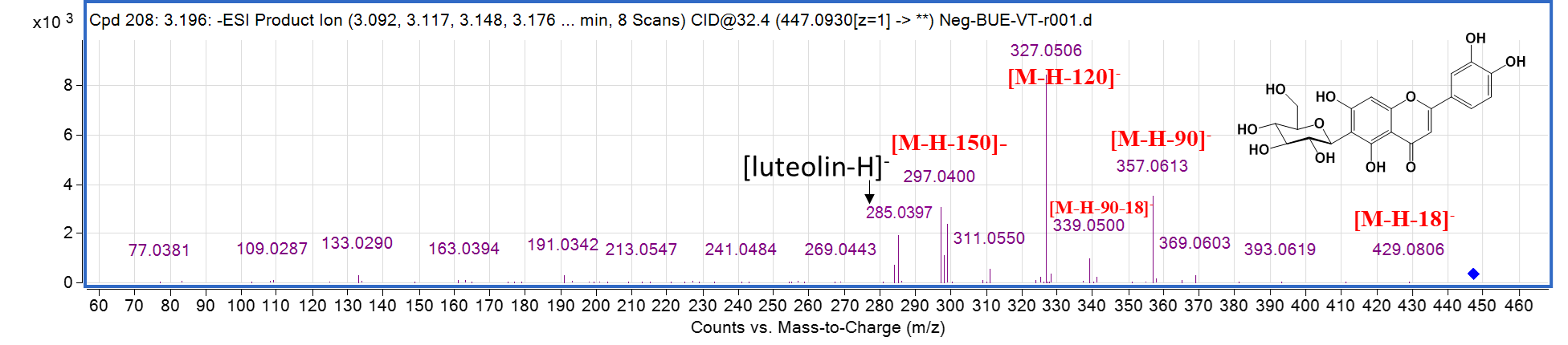 |
| ESI-MS/MS spectrum of peak (63) *via* positive ionization mode showing maslinic acid. |
| 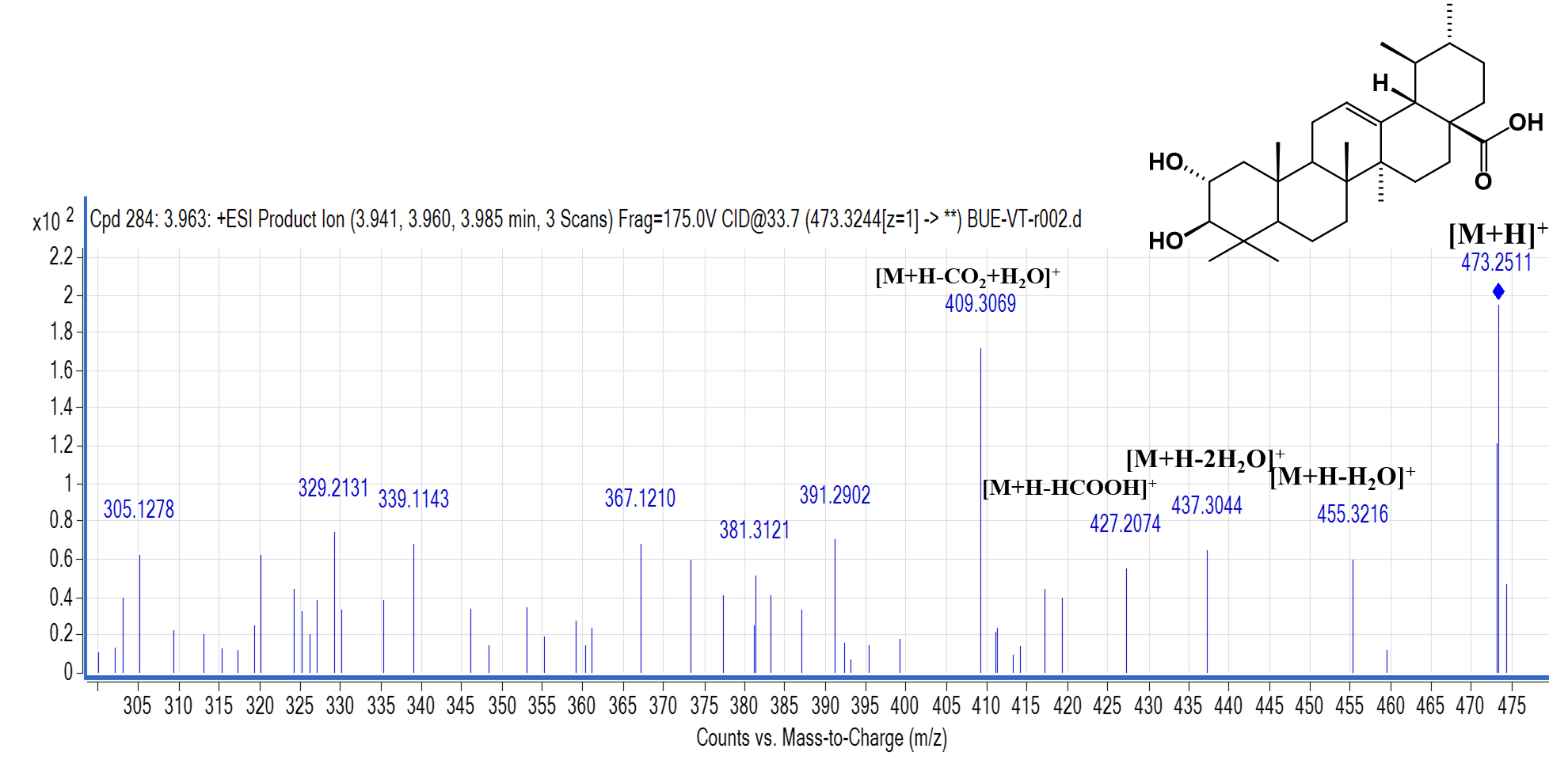 |
| ESI-MS/MS spectrum of peak (64) *via* negative ionization mode showing euscaphic acid. |
| 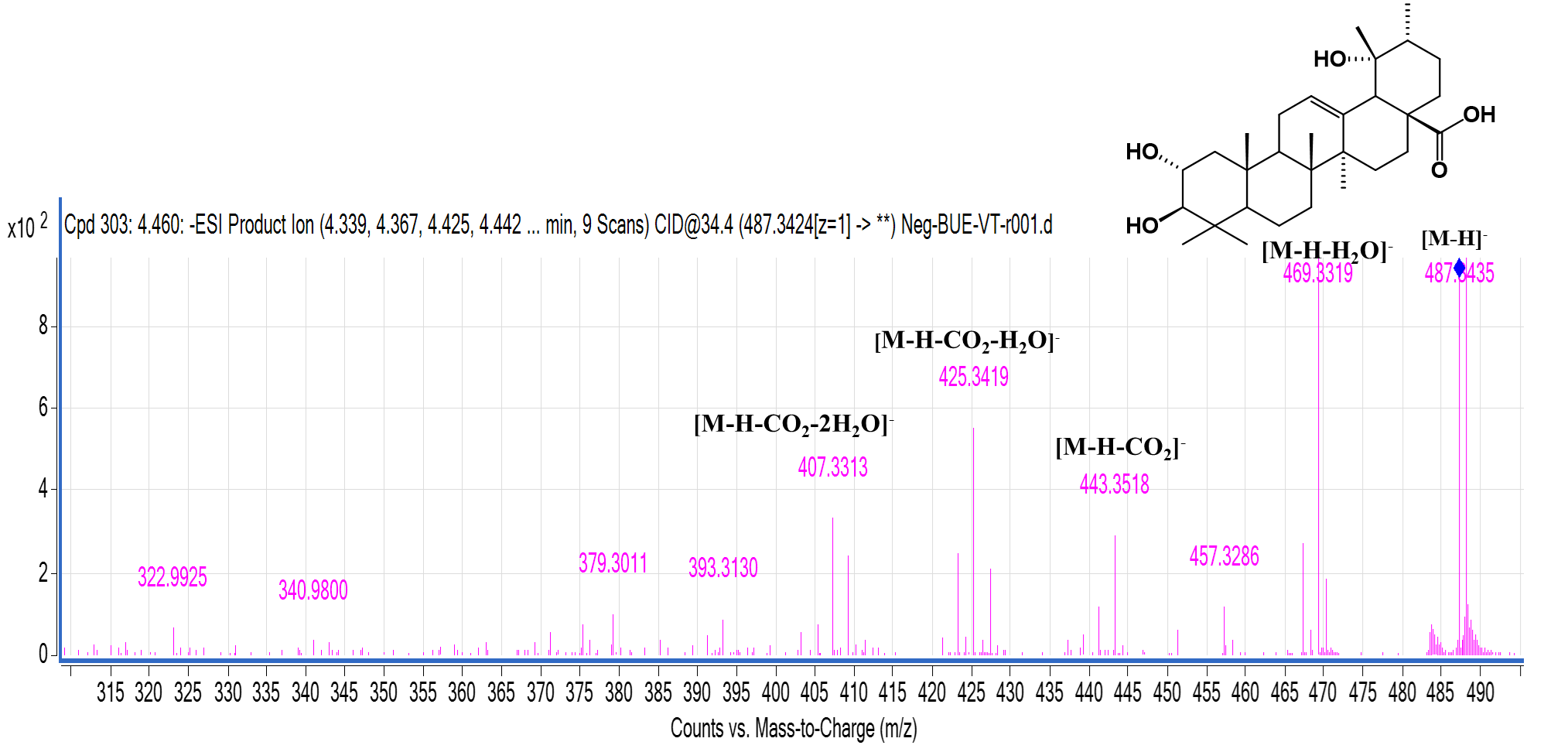 |
| ESI-MS/MS spectrum of peak (65) *via* negative ionization mode showing dihydroxy-Octadecenoic acid I. |
| 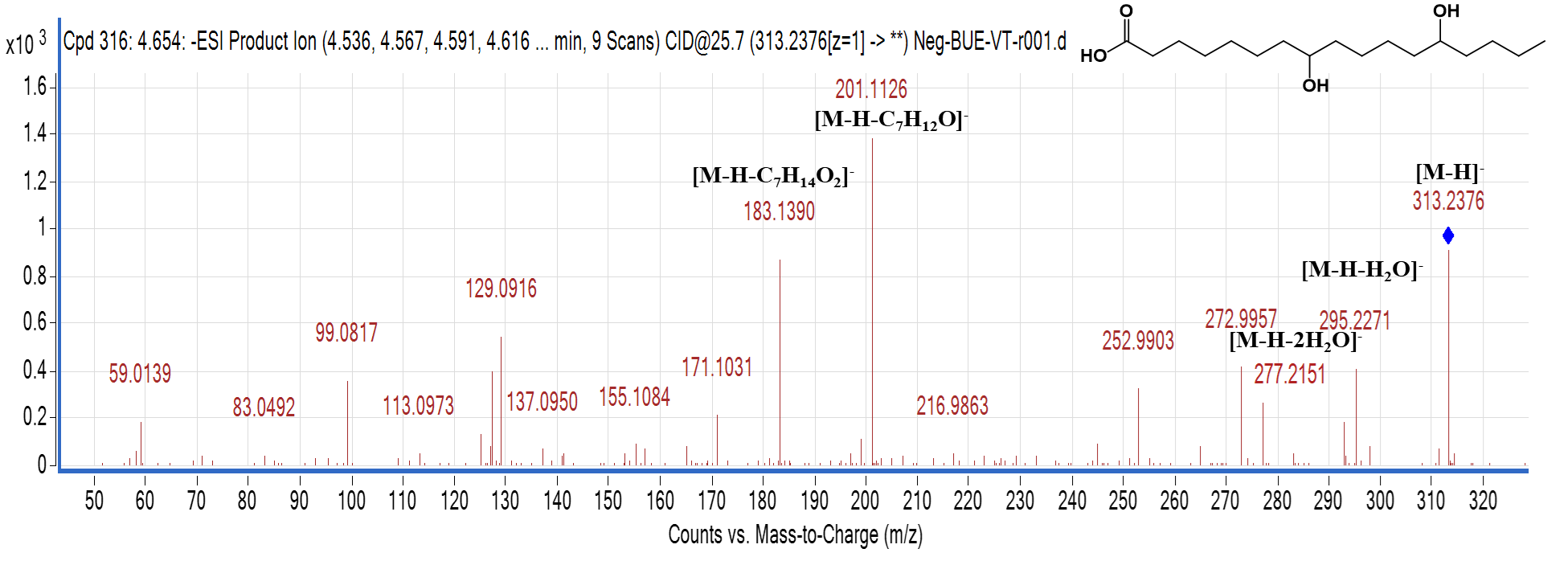 |
| ESI-MS/MS spectrum of peak (66) *via* negative ionization mode showing ursolic acid. |
| 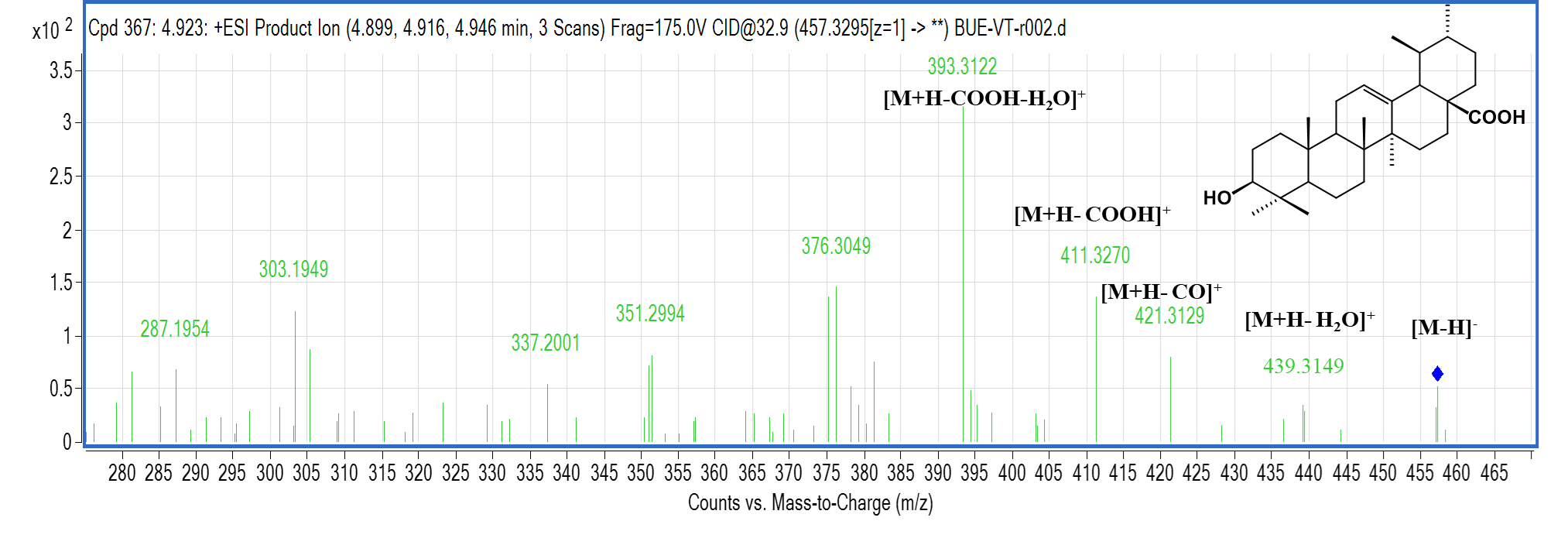 |
| ESI-MS/MS spectrum of peak (67) *via* negative ionization mode showing succinic acid. |
| 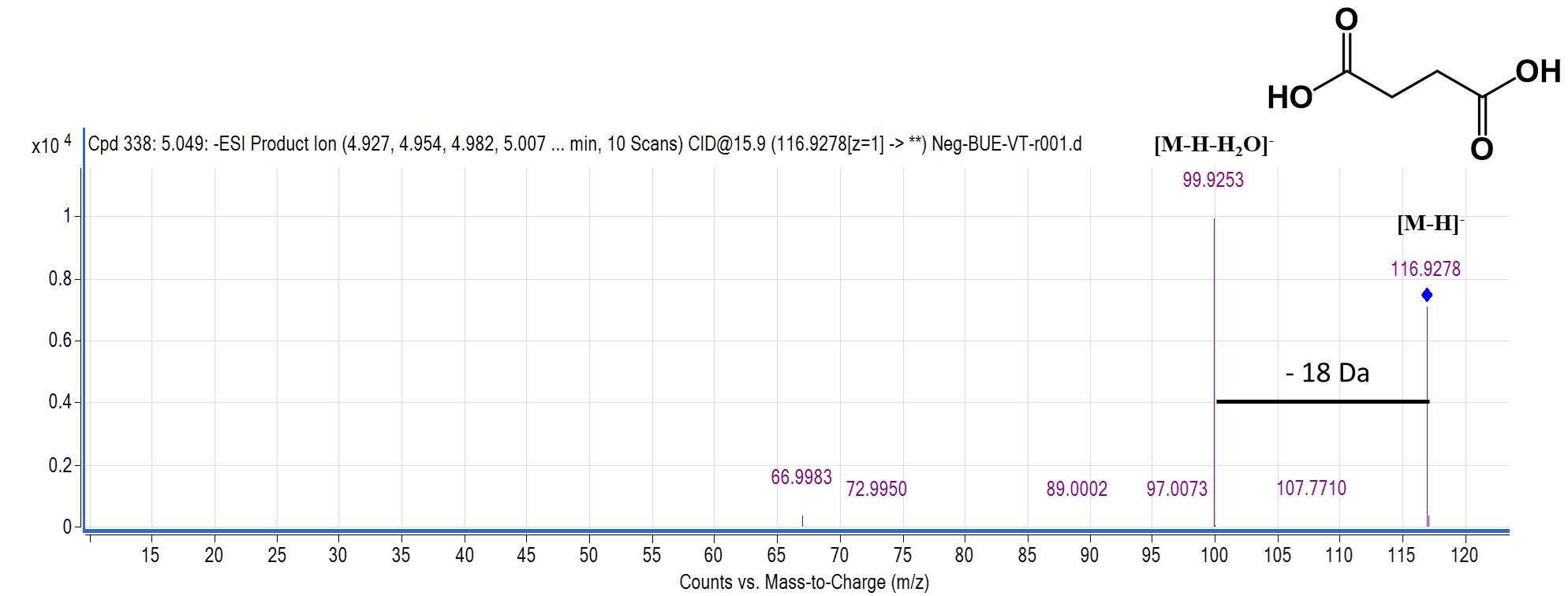 |
| ESI-MS/MS spectrum of peak (68) *via* negative ionization mode showing pomolic acid. |
| 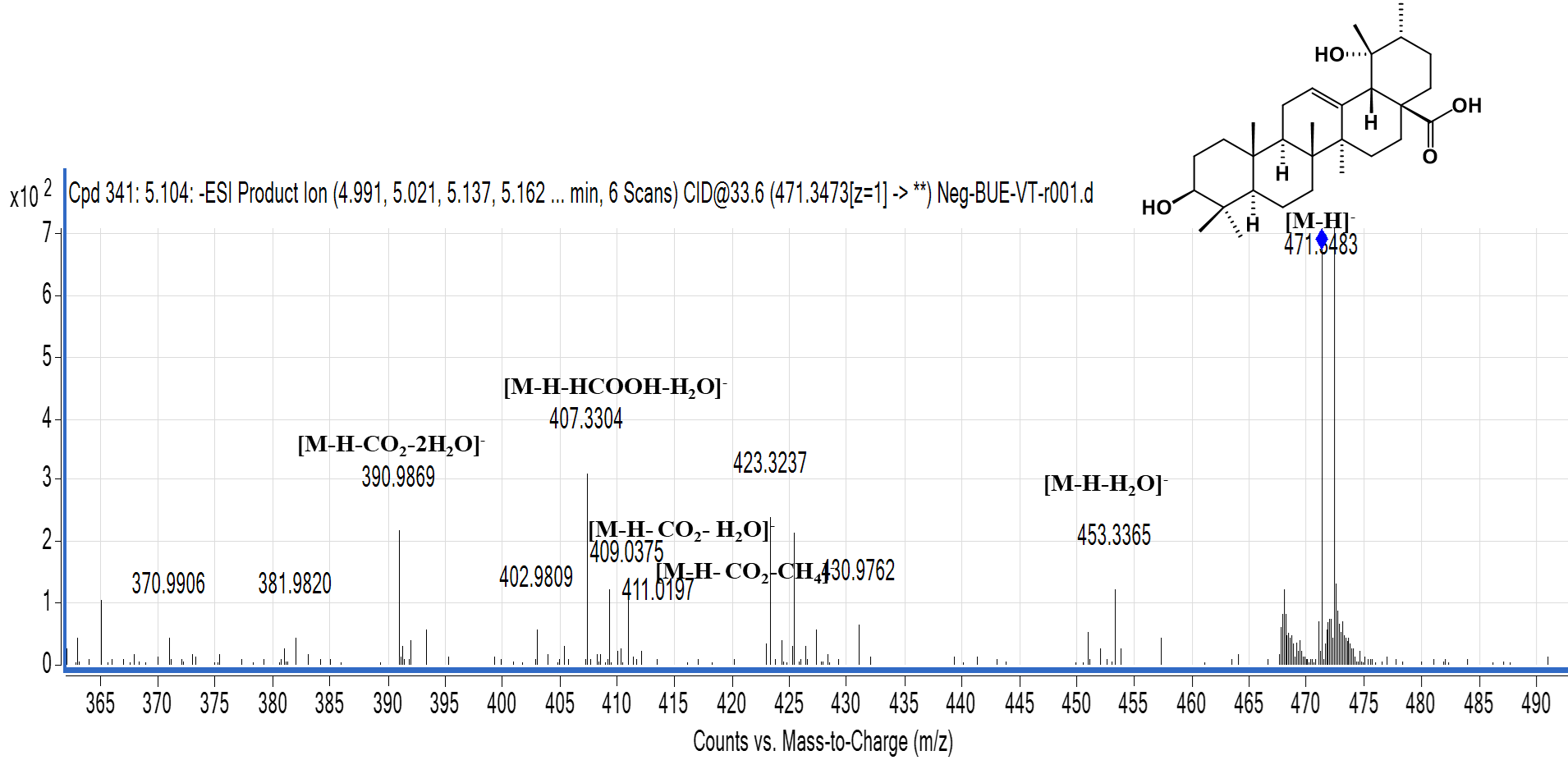 |
| ESI-MS/MS spectrum of peak (69) *via* negative ionization mode showing hydroxylinoleic acid. |
| 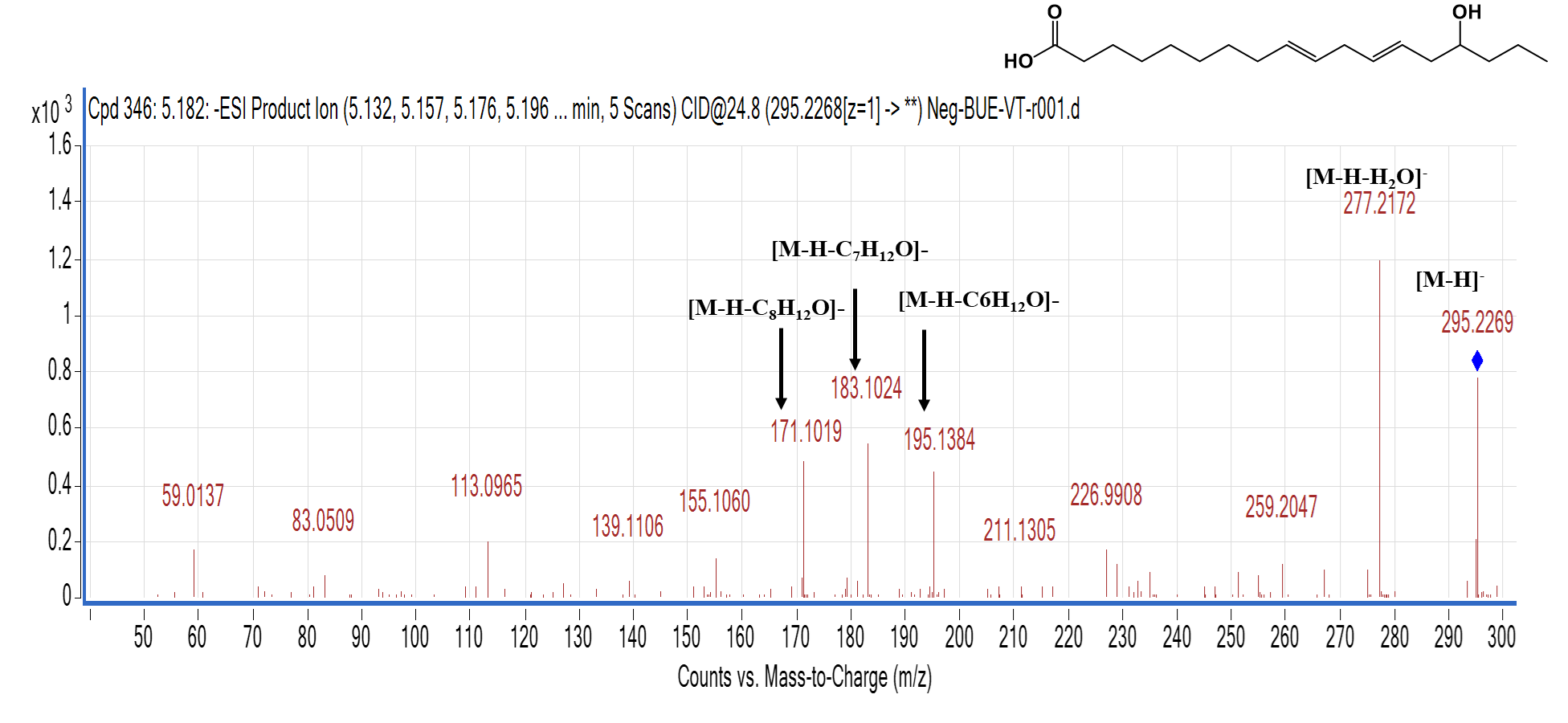 |
| ESI-MS/MS spectrum of peak (70) *via* negative ionization mode showing hydroxylinolenic acid. |
| 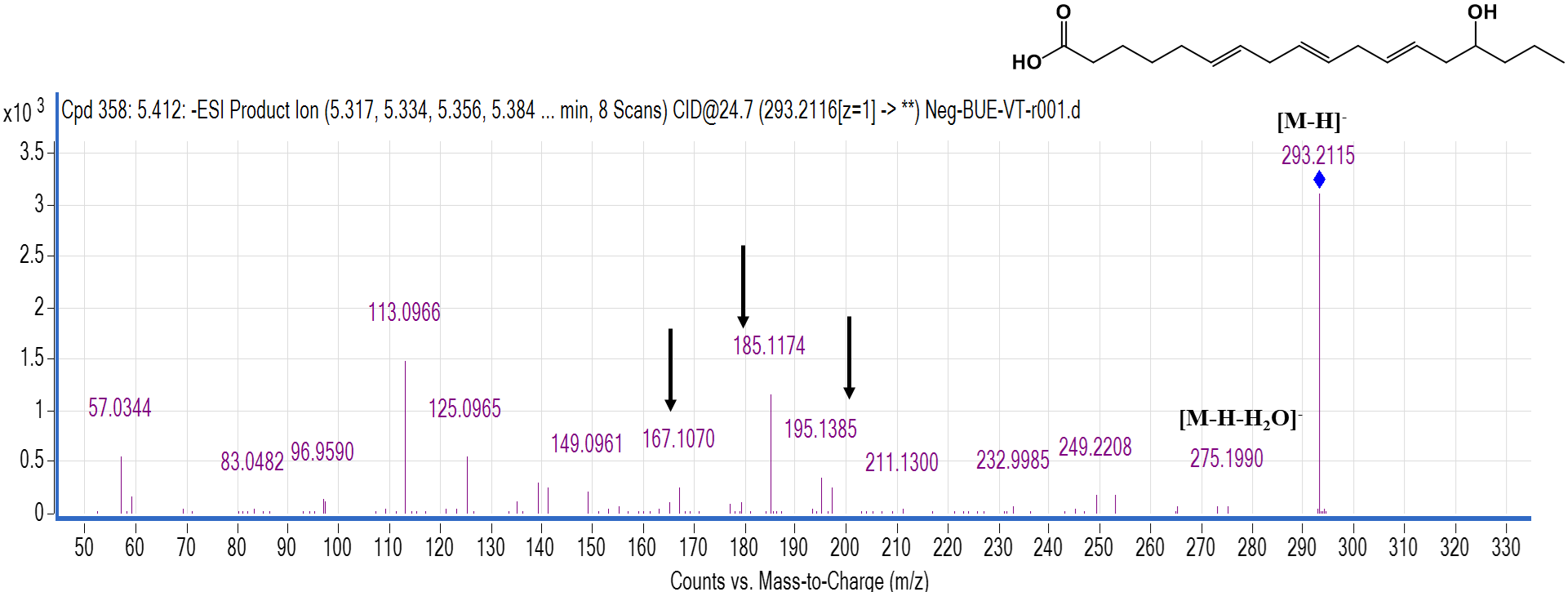 |
| ESI-MS/MS spectrum of peak (71) *via* positive ionization mode showing betulinic acid. |
| 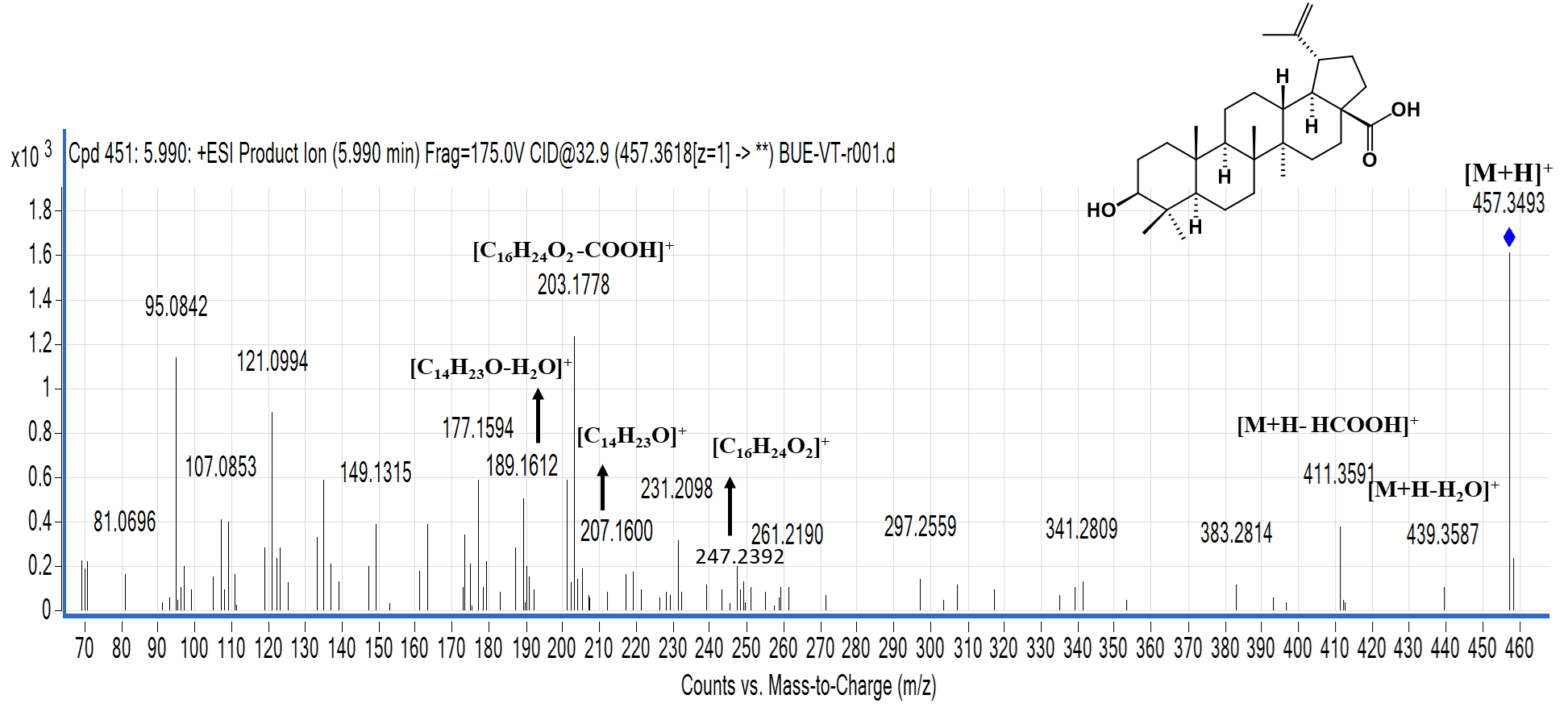 |

**Fig. S1** The detailed MS/MS spectrum of identified peaks in *V. pubescens* bark *via* positive and negative ionization modes.


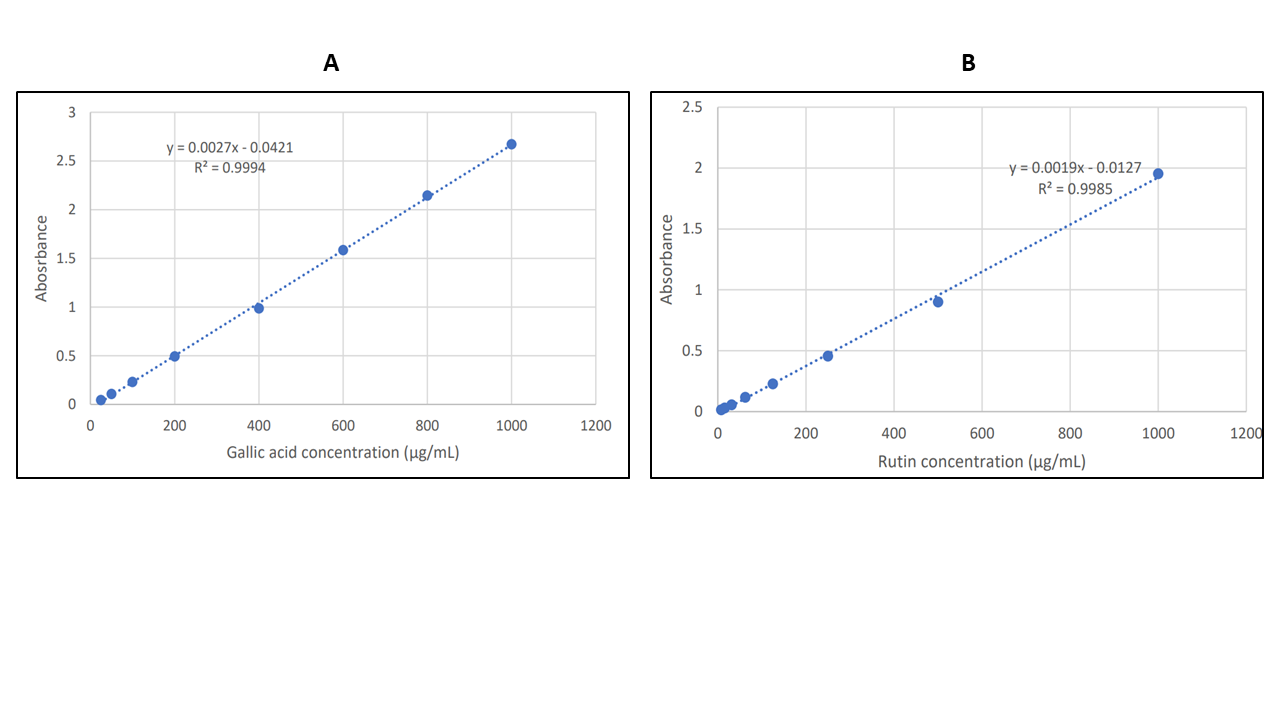
**Fig. S2** Standard calibration curves for the determination of total phenolics (**A**) and total flavonoids (**B**) concentrations.
